# Supplementary figures and images for: Therapeutic resistance in acute myeloid leukemia cells is mediated by a novel ATM/mTOR pathway regulating oxidative phosphorylation (part 1 of 2)
Source: eLife. 2022 Oct 19;11:e79940. doi: 10.7554/eLife.79940 (PMC9645811; doi:10.7554/eLife.79940)

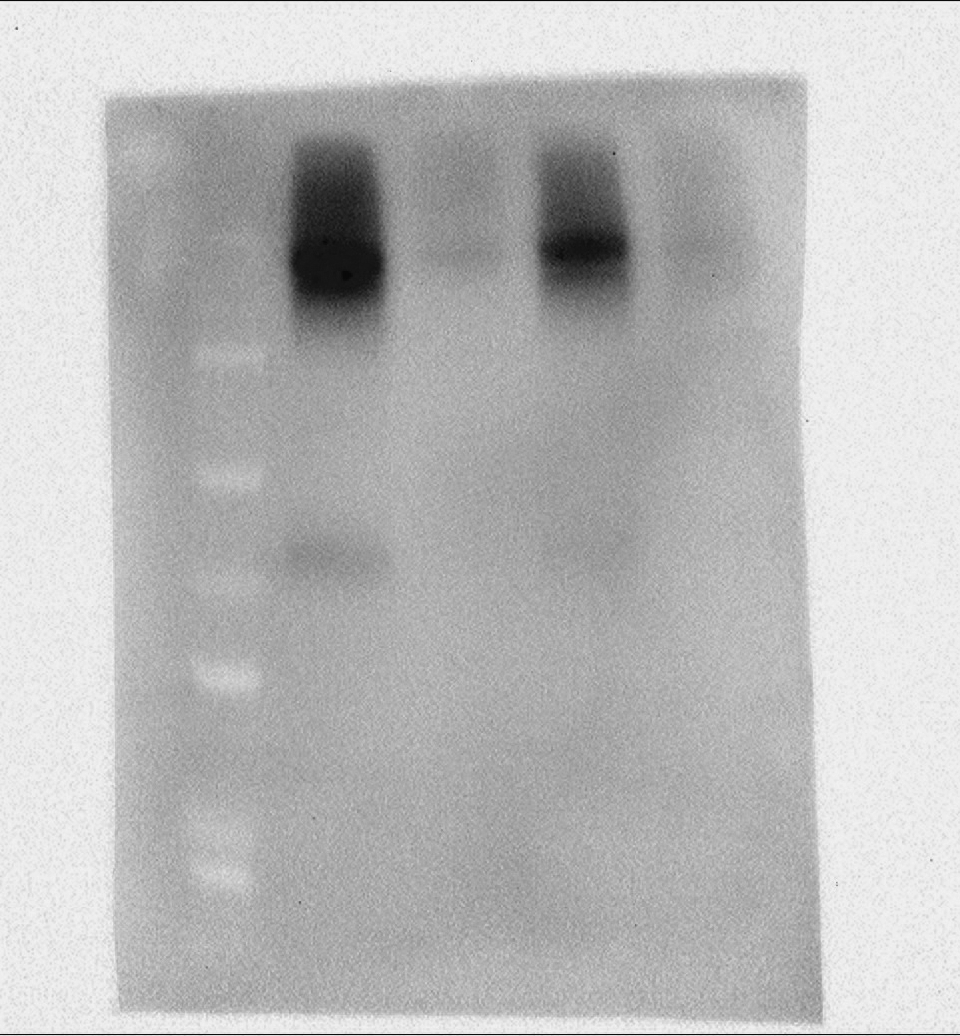

Supplement: Figure 1—source data 1. [file elife-79940-fig1-data1.zip › 1C-pFLT3(Y591) unlabeled.tif]

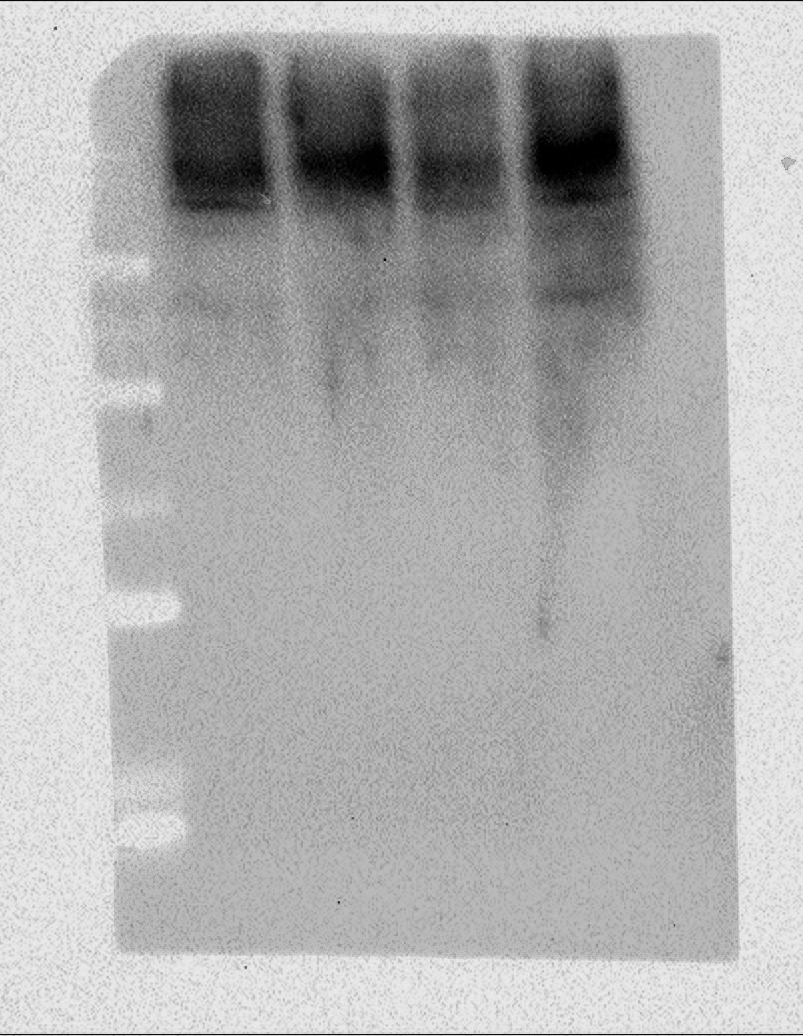

Supplement: Figure 1—source data 1. [file elife-79940-fig1-data1.zip › 1C-FLT3 unlabeled.tif]

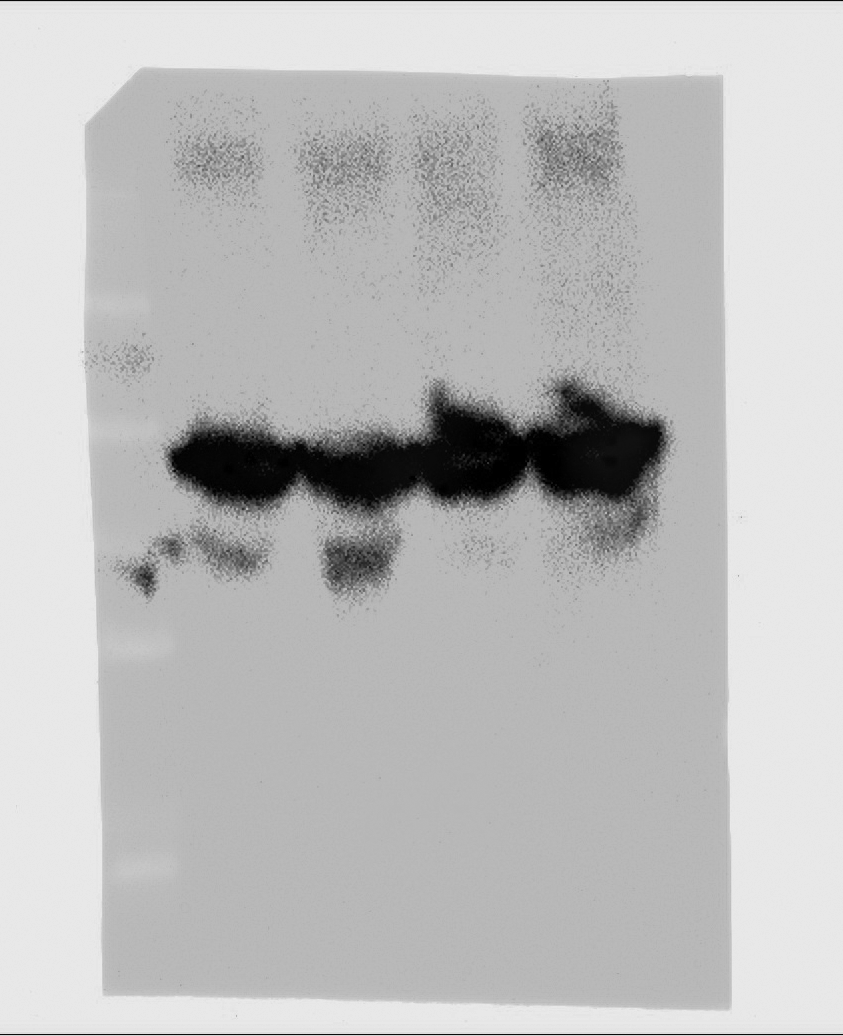

Supplement: Figure 1—source data 1. [file elife-79940-fig1-data1.zip › 1C-Tublin unlabeled.tif]

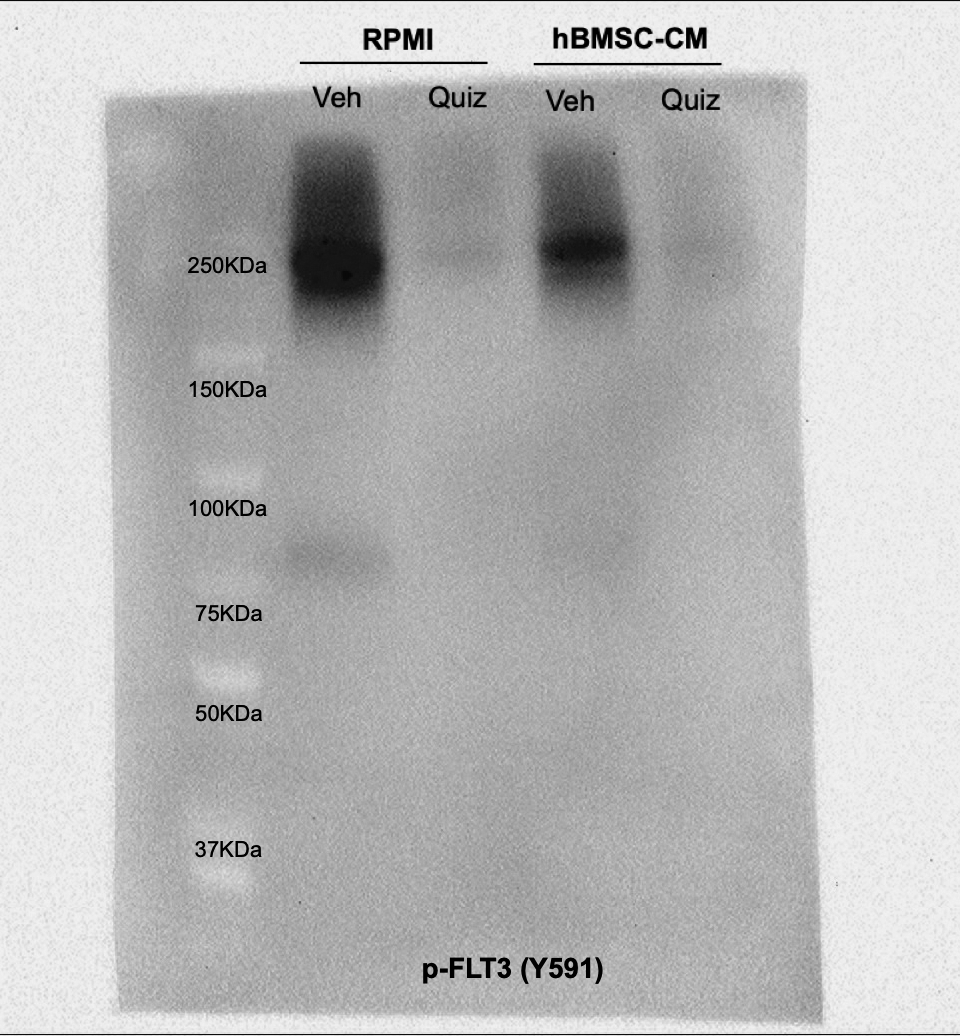

Supplement: Figure 1—source data 2. [file elife-79940-fig1-data2.zip › 1C-pFLT3(Y591) labeled .tiff]

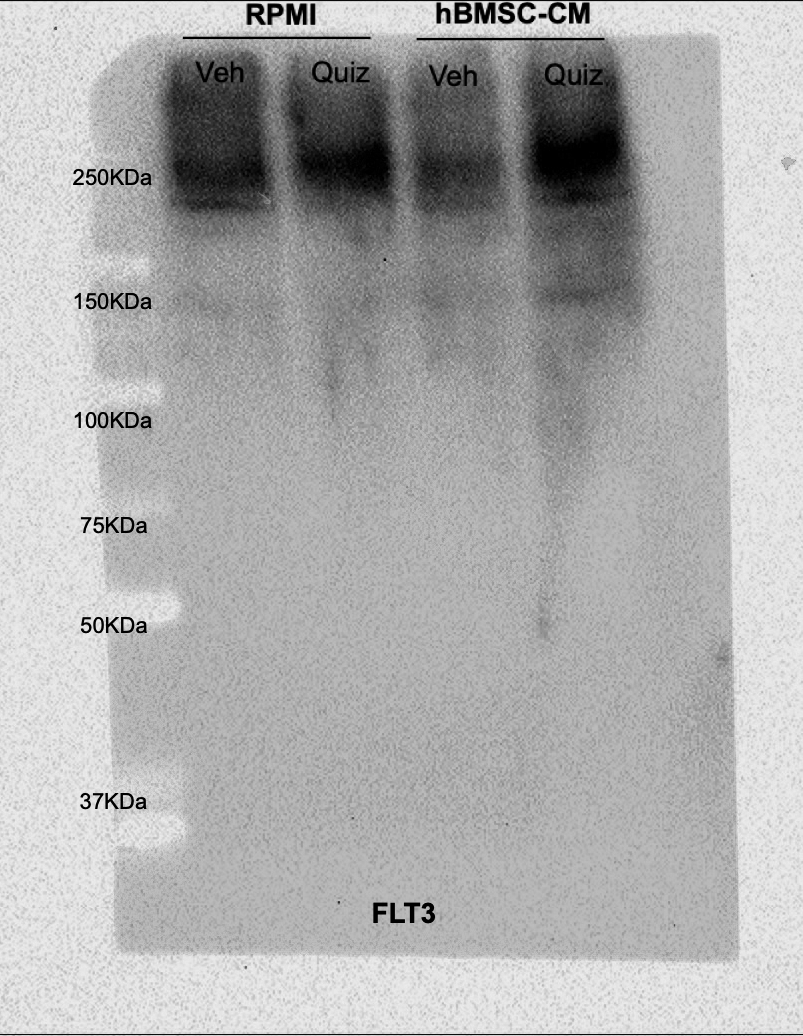

Supplement: Figure 1—source data 2. [file elife-79940-fig1-data2.zip › 1C-FLT3 labeled .tiff]

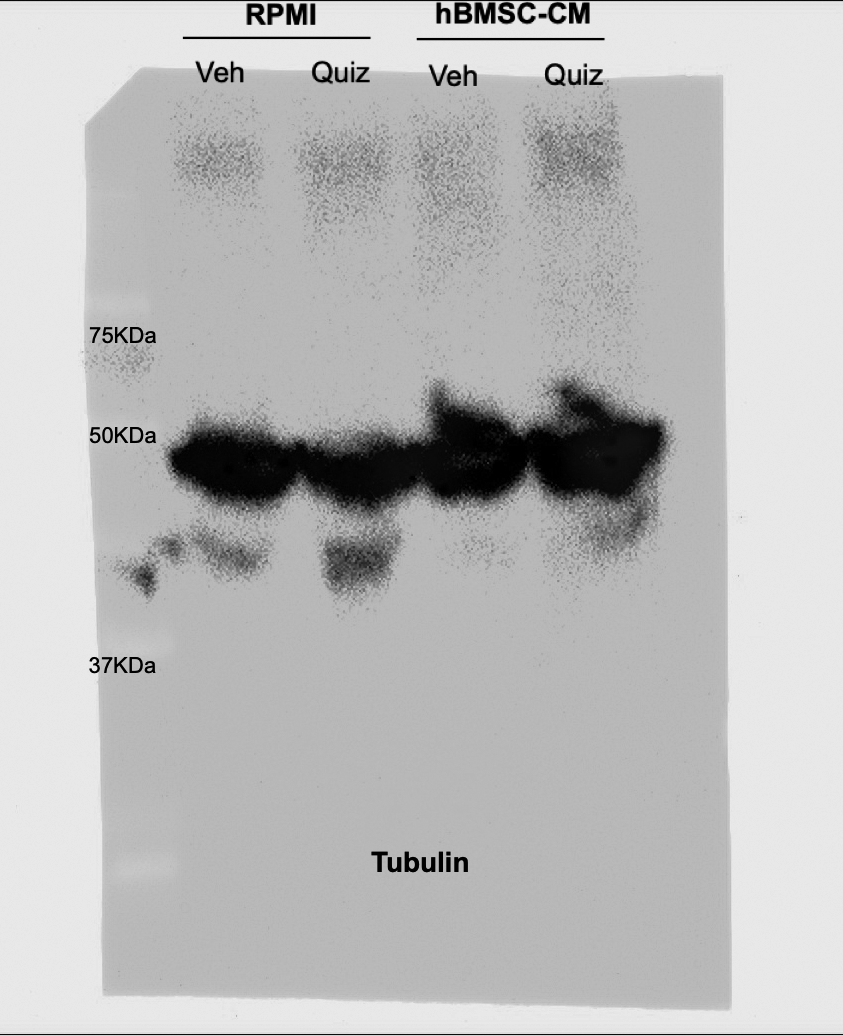

Supplement: Figure 1—source data 2. [file elife-79940-fig1-data2.zip › 1C-Tublin labeled.tiff]

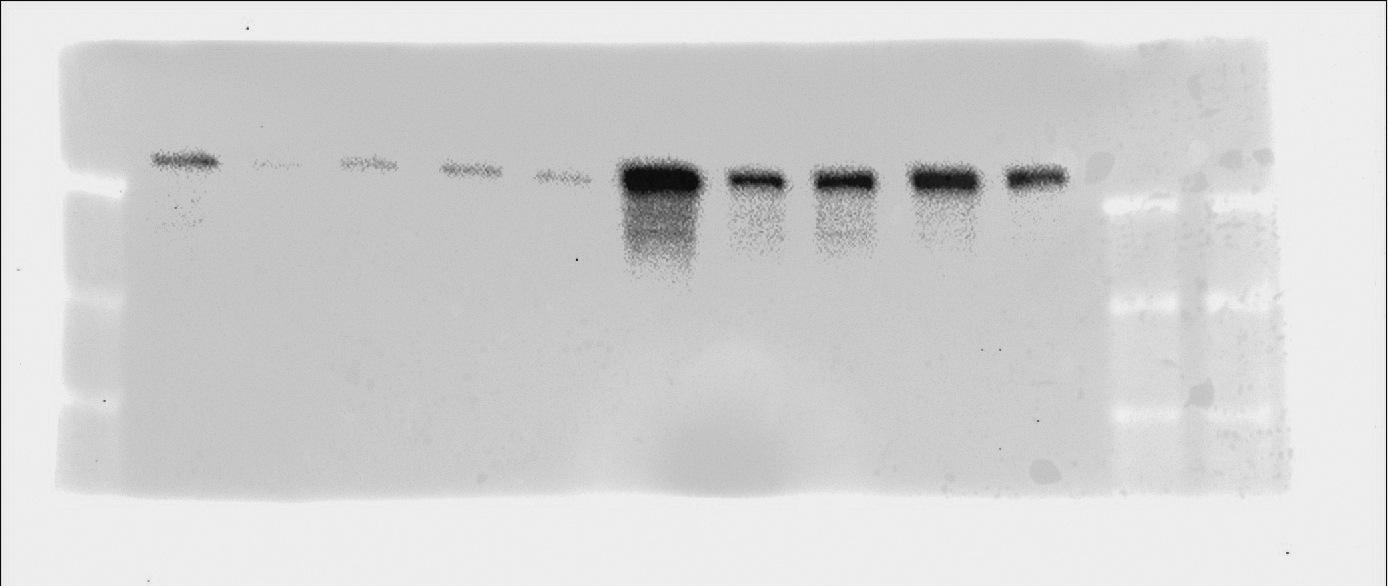

Supplement: Figure 2—source data 1. [file elife-79940-fig2-data1.zip › 2D-pMTORC1 (s2448) unlabeled.tif]

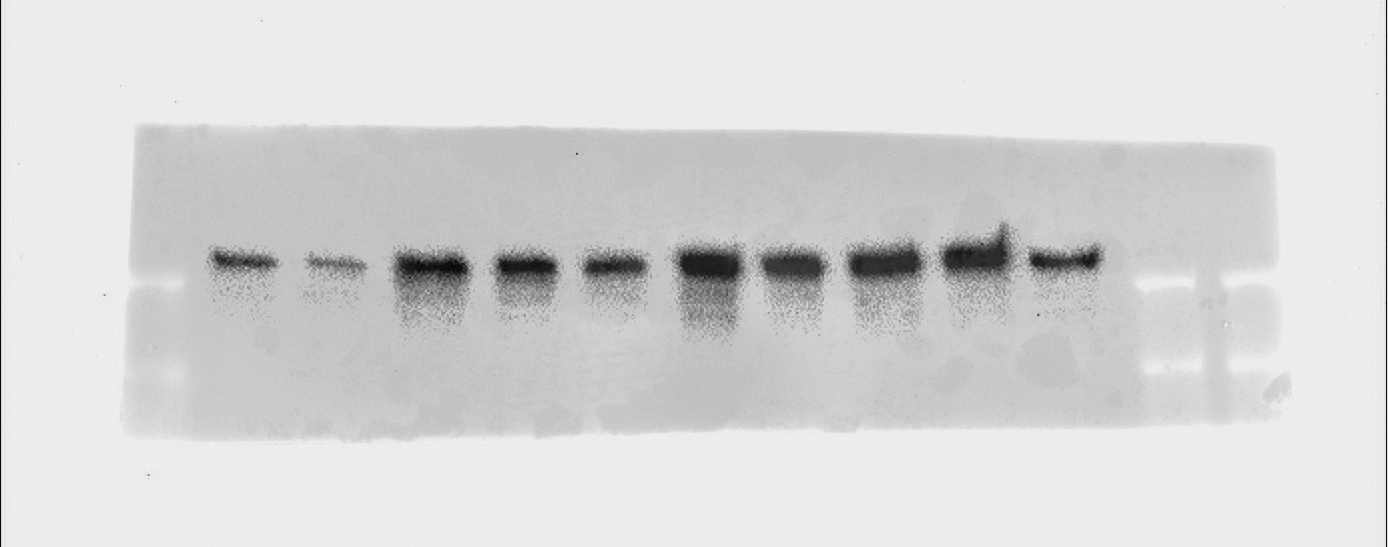

Supplement: Figure 2—source data 1. [file elife-79940-fig2-data1.zip › 2D-mTOR unlabeled.tif]

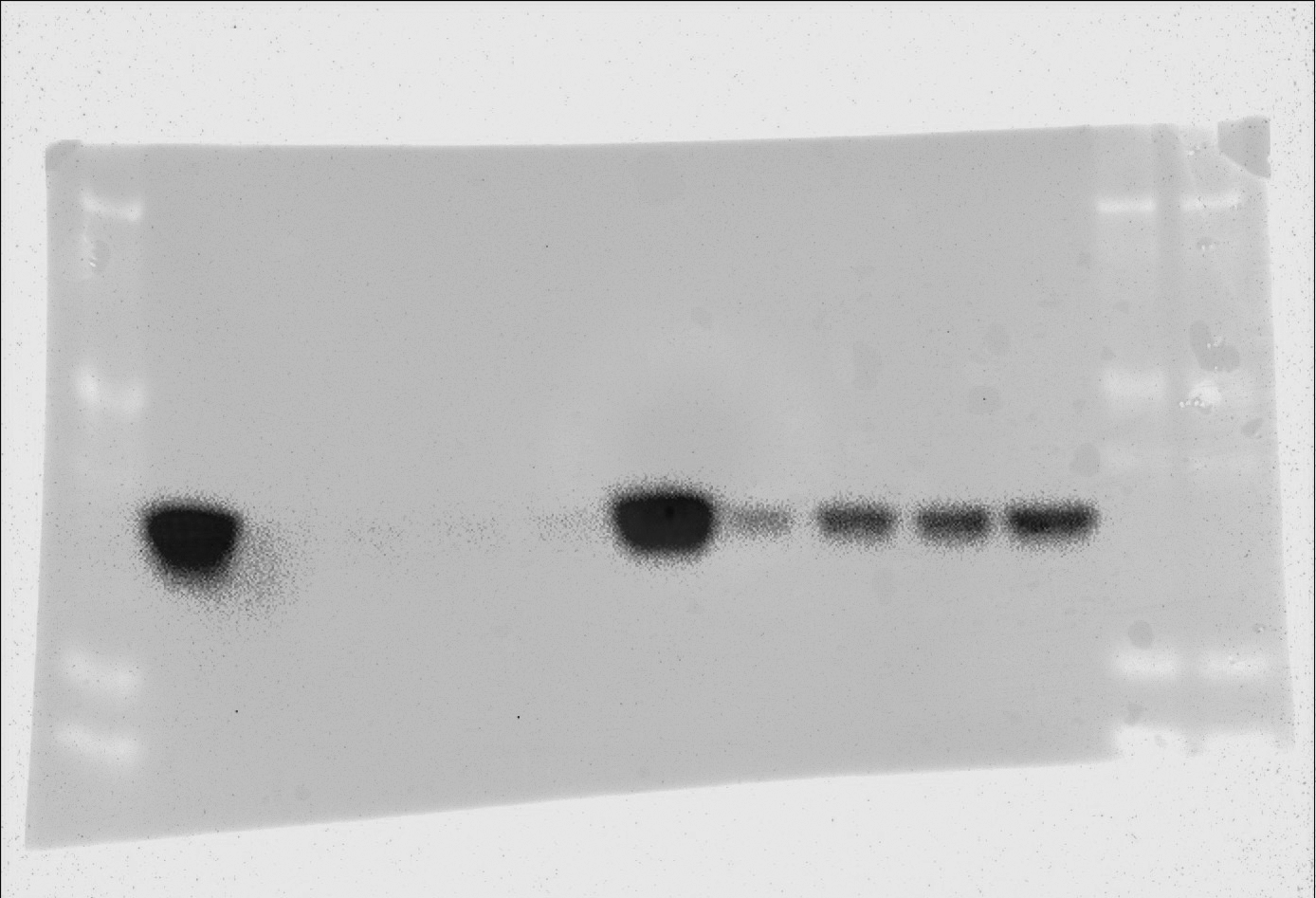

Supplement: Figure 2—source data 1. [file elife-79940-fig2-data1.zip › 2D-pS6 (s240:244) unlabeled.tif]

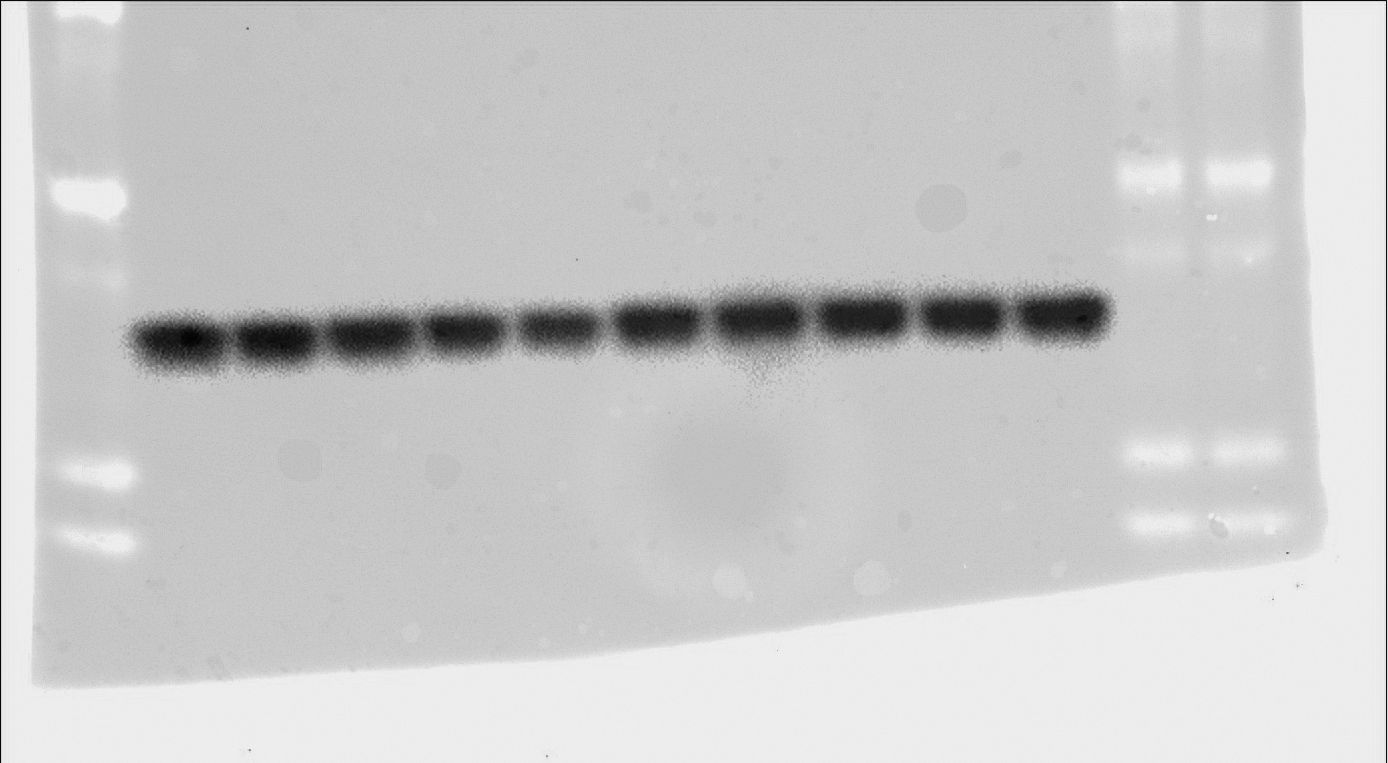

Supplement: Figure 2—source data 1. [file elife-79940-fig2-data1.zip › 2D-S6 unlabeled.tif]

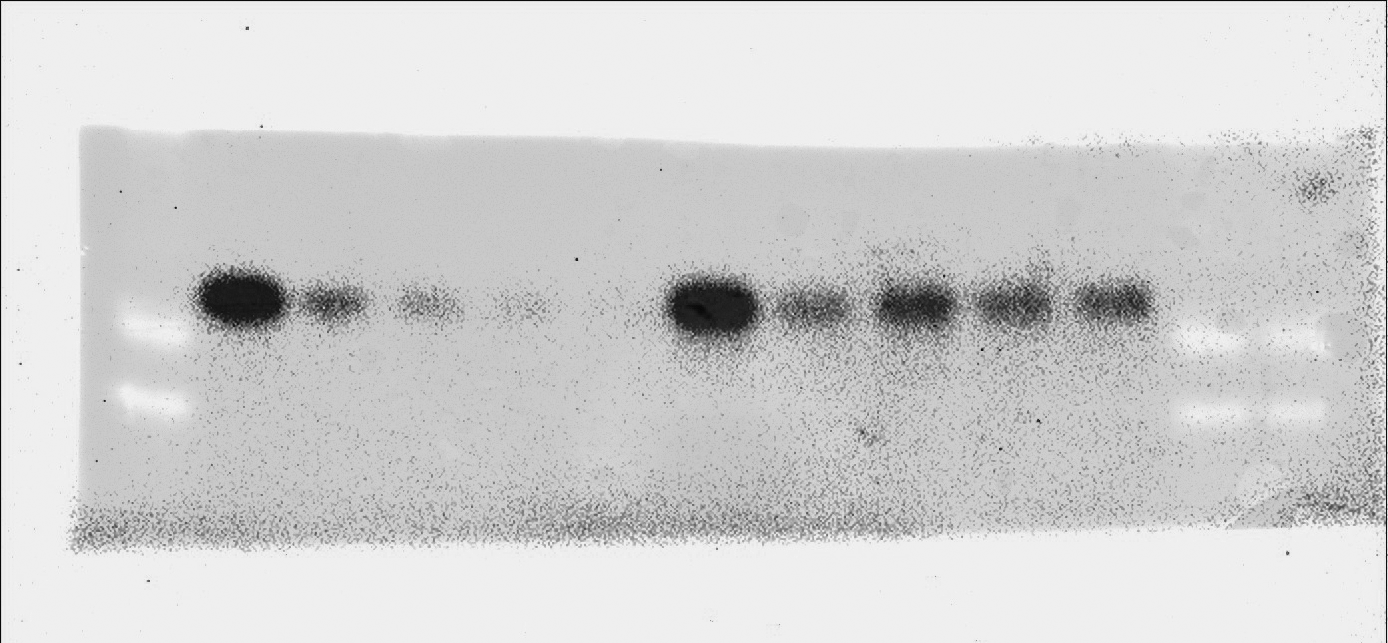

Supplement: Figure 2—source data 1. [file elife-79940-fig2-data1.zip › 2D-p4EBP1 (s235:236) unlabeled.tif]

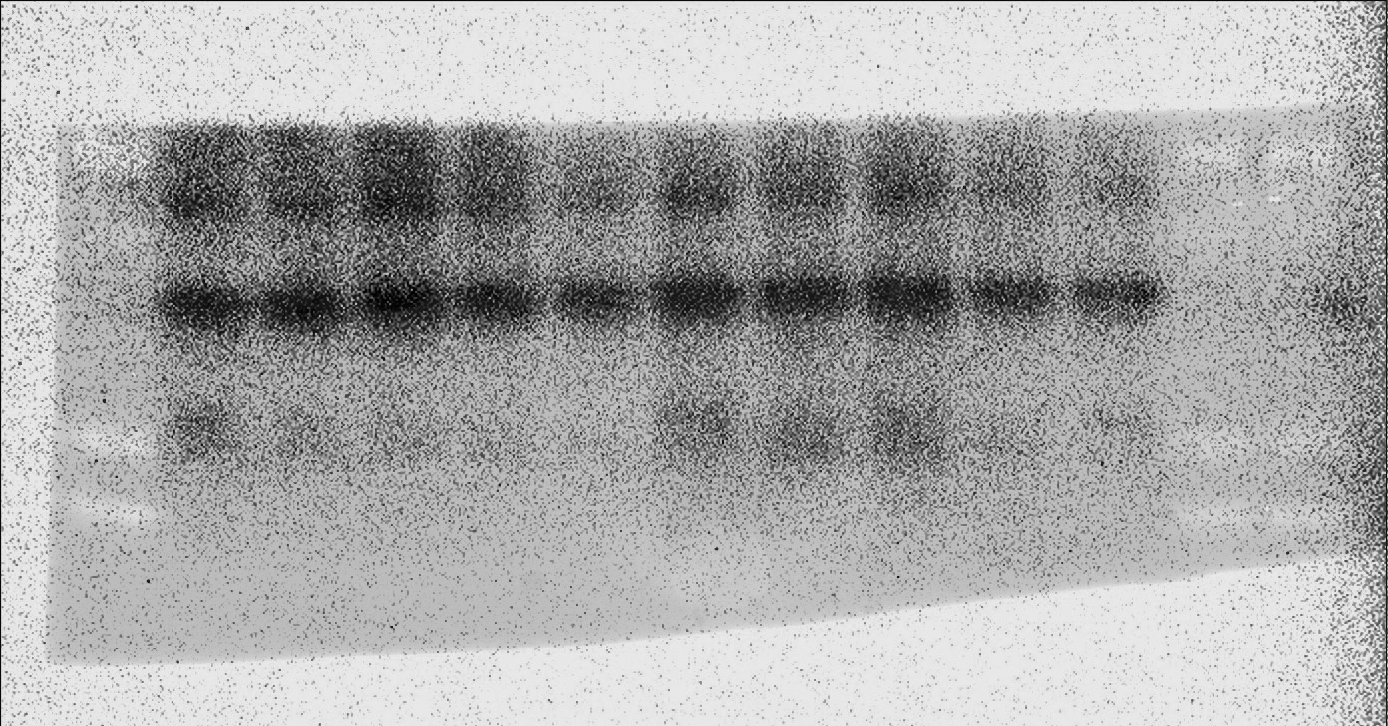

Supplement: Figure 2—source data 1. [file elife-79940-fig2-data1.zip › 2D-4EBP1 unlabeled.tif]

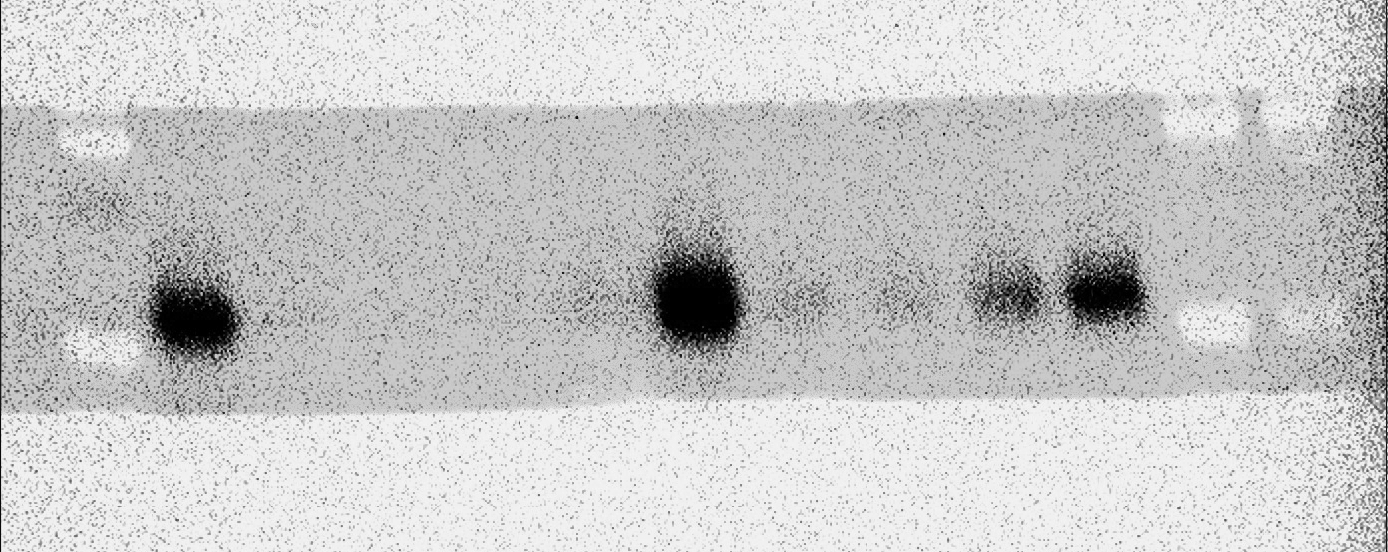

Supplement: Figure 2—source data 1. [file elife-79940-fig2-data1.zip › 2D-cMYC unlabeled.tif]

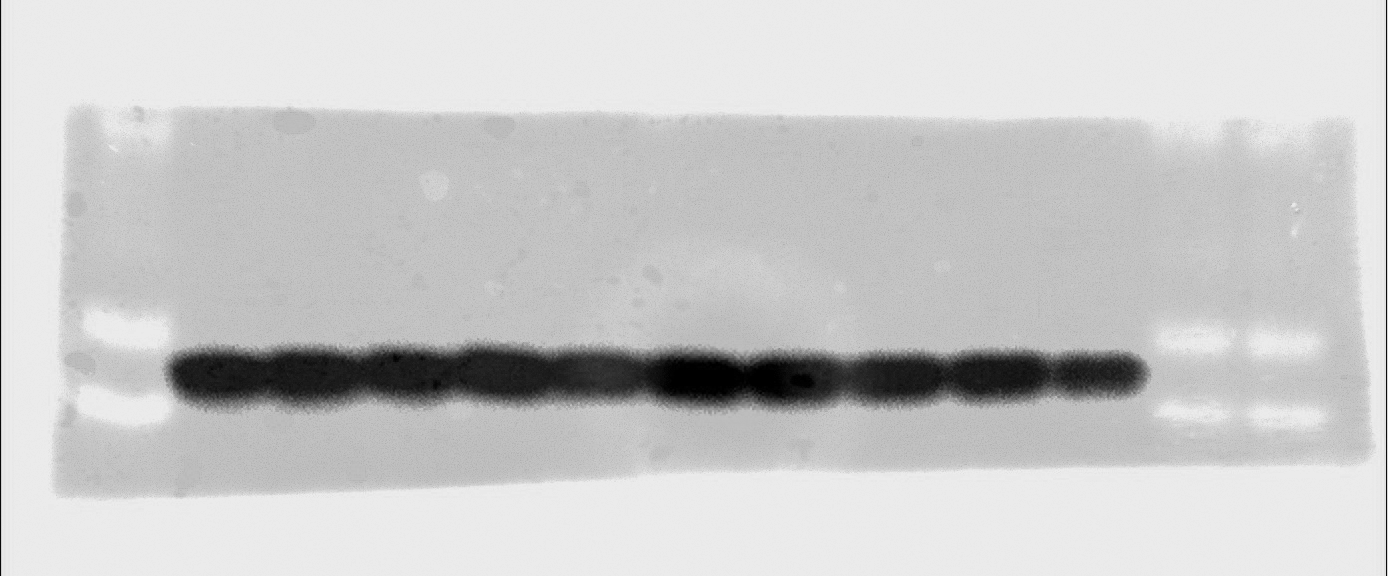

Supplement: Figure 2—source data 1. [file elife-79940-fig2-data1.zip › 2D-H3 unlabeled.tif]

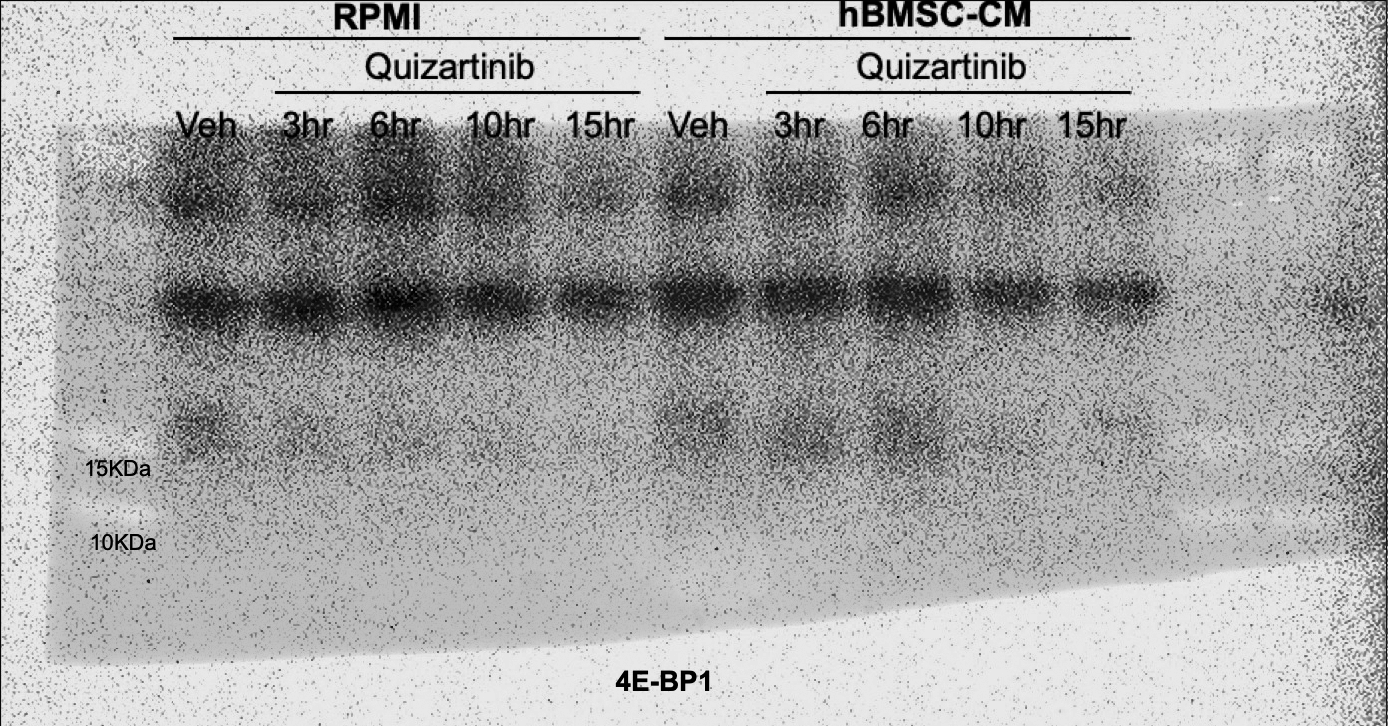

Supplement: Figure 2—source data 2. [file elife-79940-fig2-data2.zip › 2D-4EBP1 labeled.tiff]

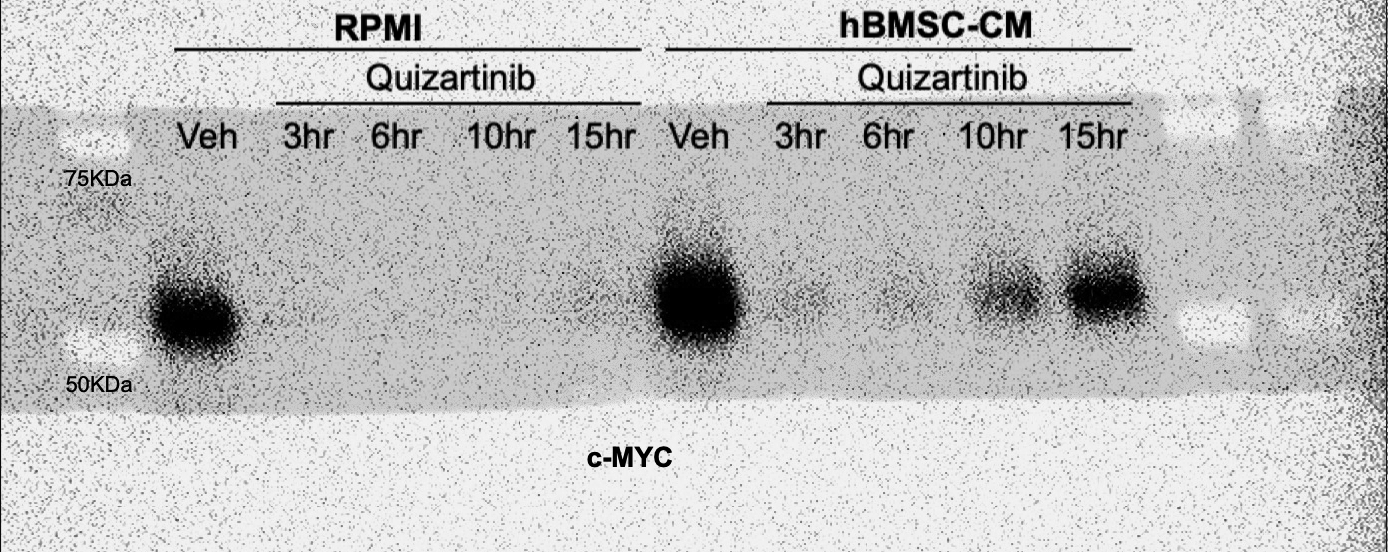

Supplement: Figure 2—source data 2. [file elife-79940-fig2-data2.zip › 2D-cMYC labeled .tiff]

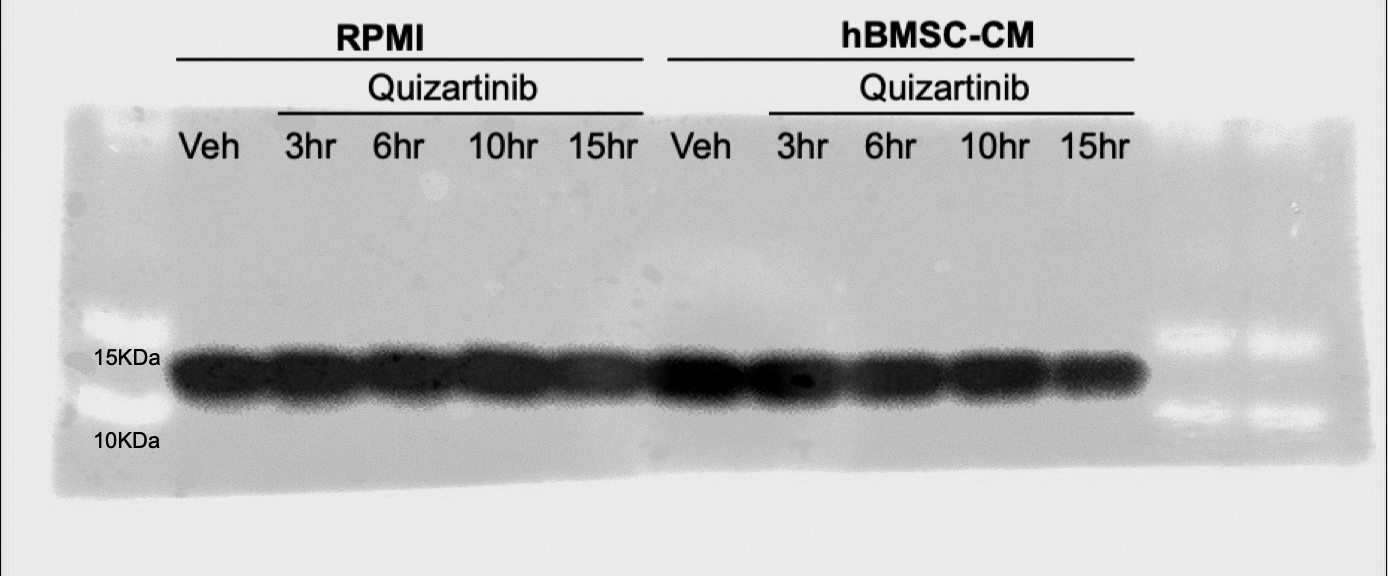

Supplement: Figure 2—source data 2. [file elife-79940-fig2-data2.zip › 2D-H3 labeled.tiff]

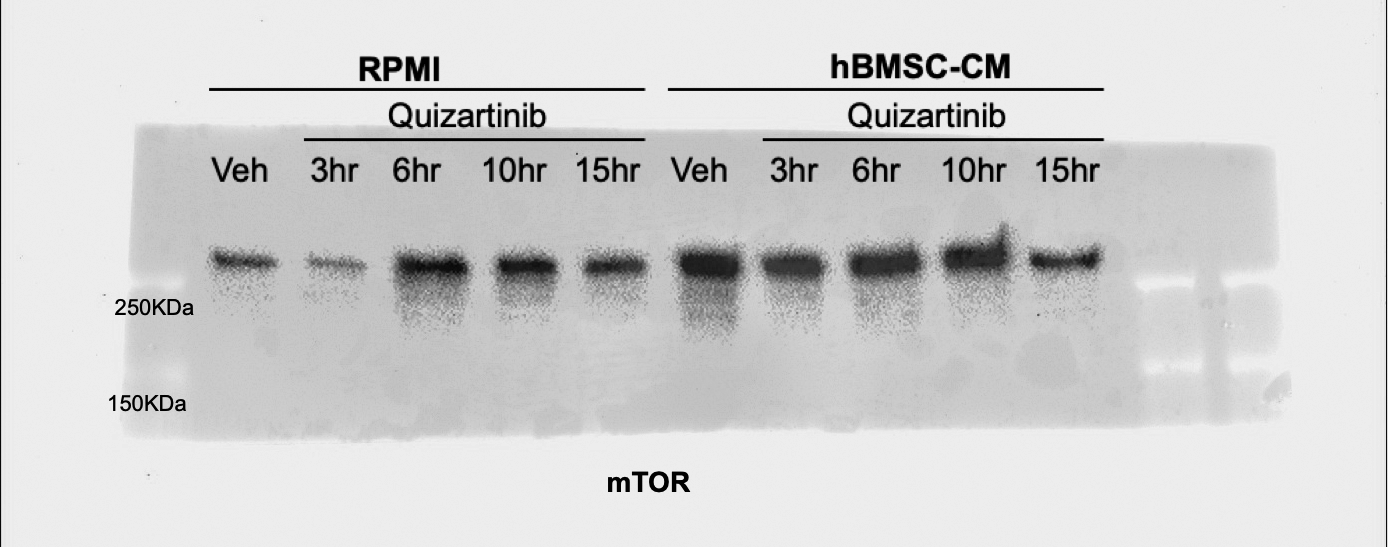

Supplement: Figure 2—source data 2. [file elife-79940-fig2-data2.zip › 2D-mTOR labeled.tiff]

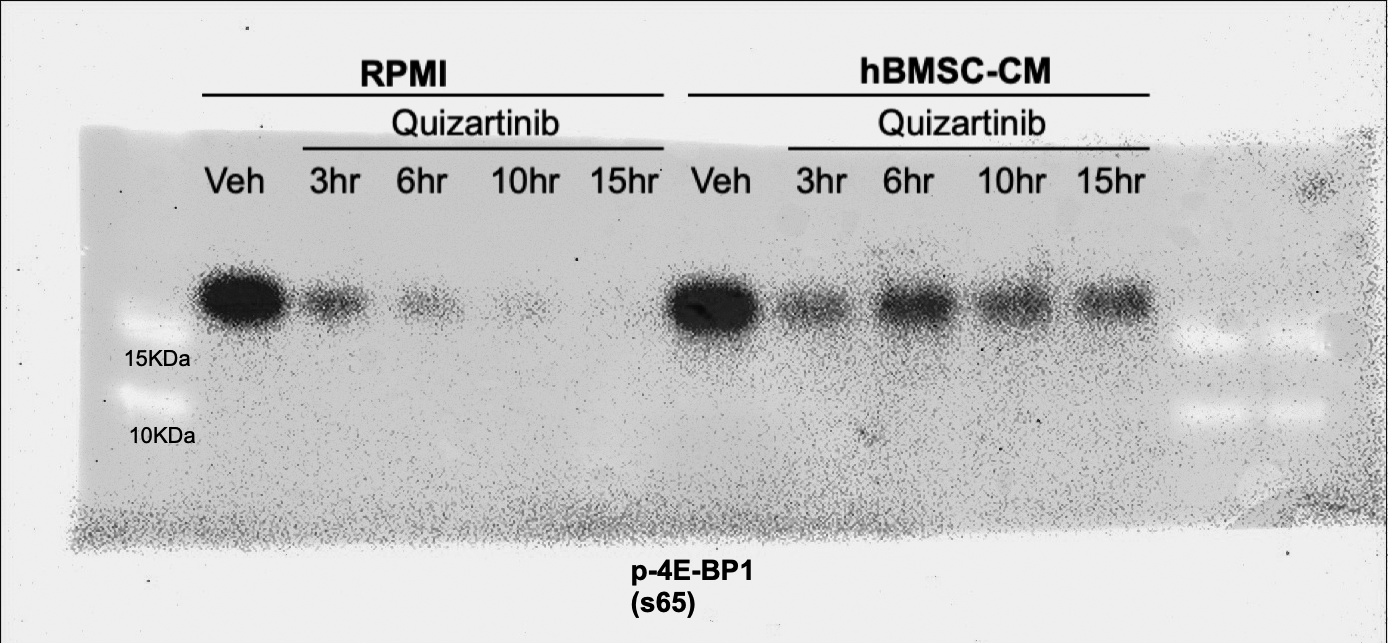

Supplement: Figure 2—source data 2. [file elife-79940-fig2-data2.zip › 2D-p4EBP1 (s65) labeled.tiff]

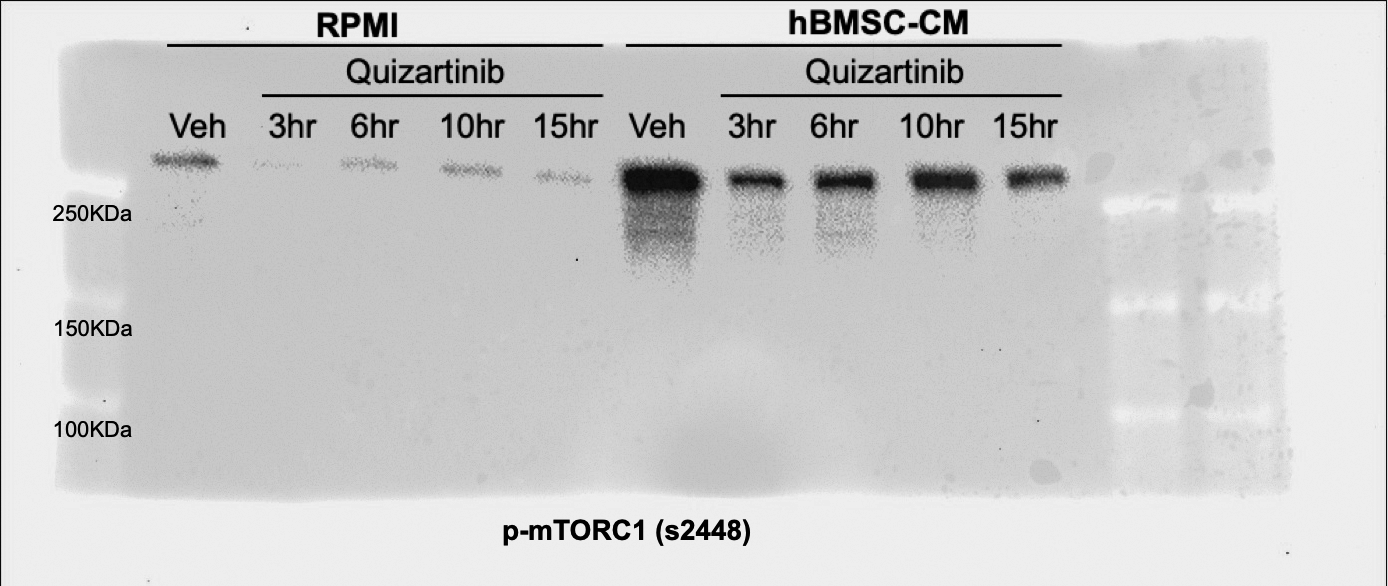

Supplement: Figure 2—source data 2. [file elife-79940-fig2-data2.zip › 2D-pMTORC1 (s2448) labeled.tiff]

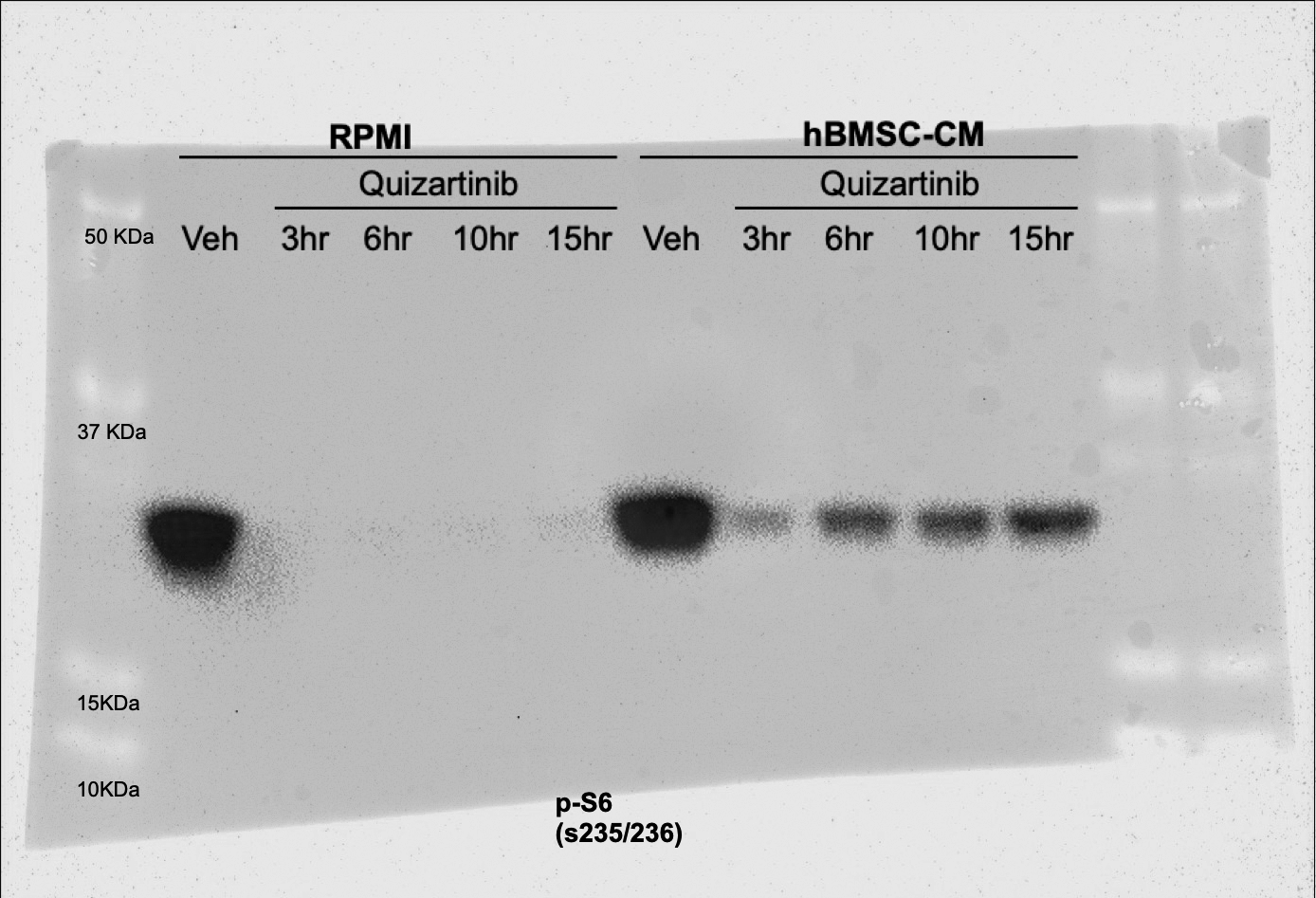

Supplement: Figure 2—source data 2. [file elife-79940-fig2-data2.zip › 2D-pS6 (s235:236) labeled.tiff]

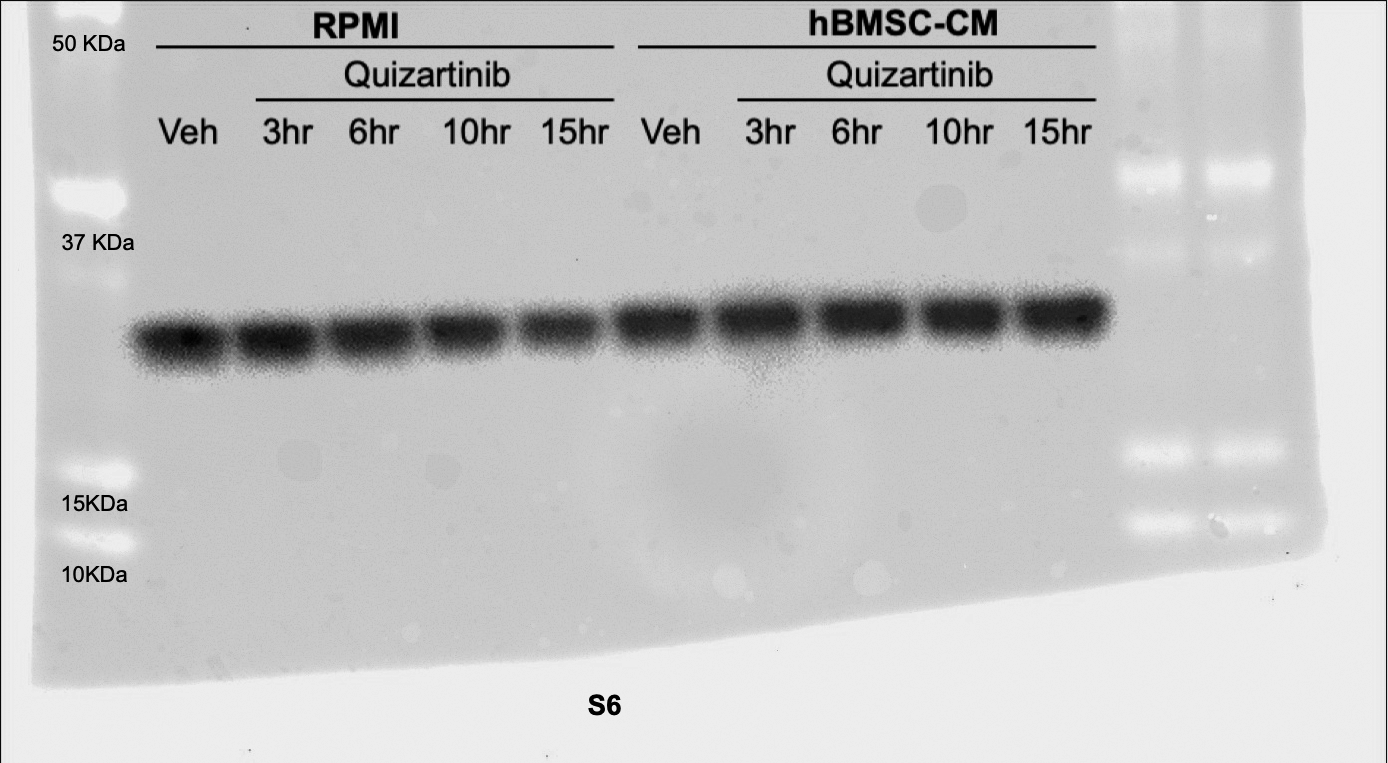

Supplement: Figure 2—source data 2. [file elife-79940-fig2-data2.zip › 2D-S6 labeled .tiff]

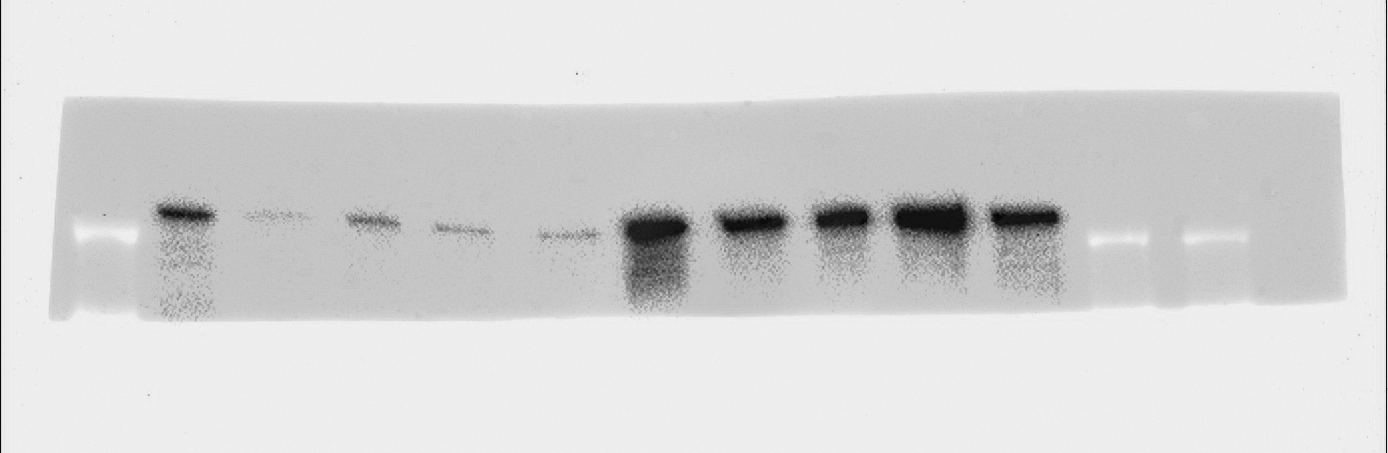

Supplement: Figure 2—figure supplement 2—source data 1. [file elife-79940-fig2-figsupp2-data1.zip › Fig 2-figure supplement 1- pMTORC1 (s2448) unlabeled.tif]

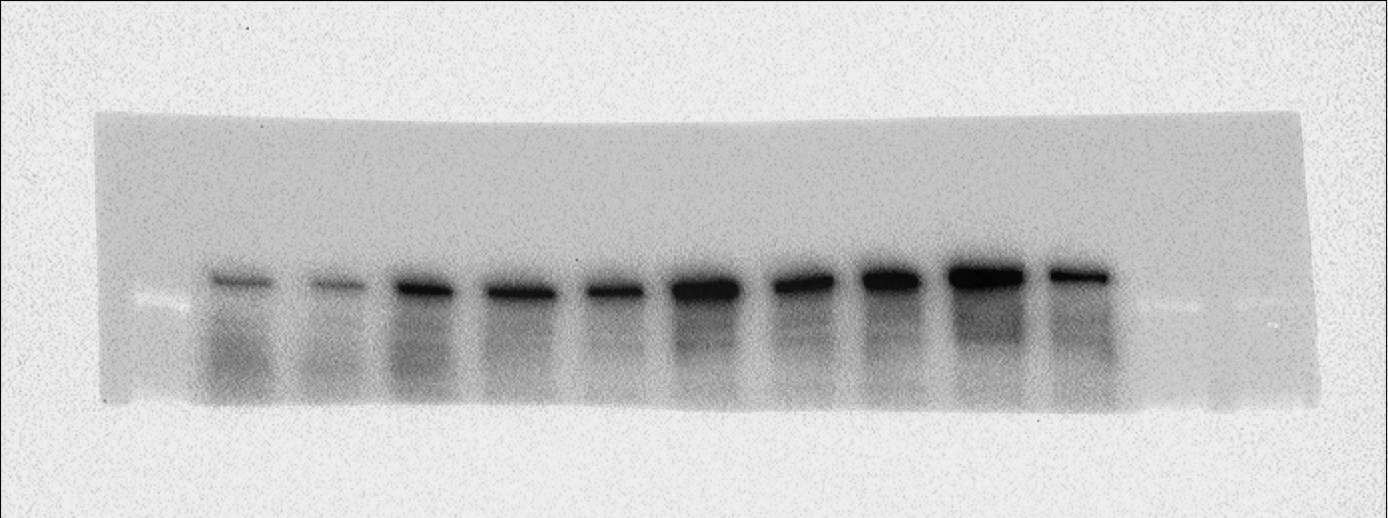

Supplement: Figure 2—figure supplement 2—source data 1. [file elife-79940-fig2-figsupp2-data1.zip › Fig 2-figure supplement 1-mTOR unlabeled.tif]

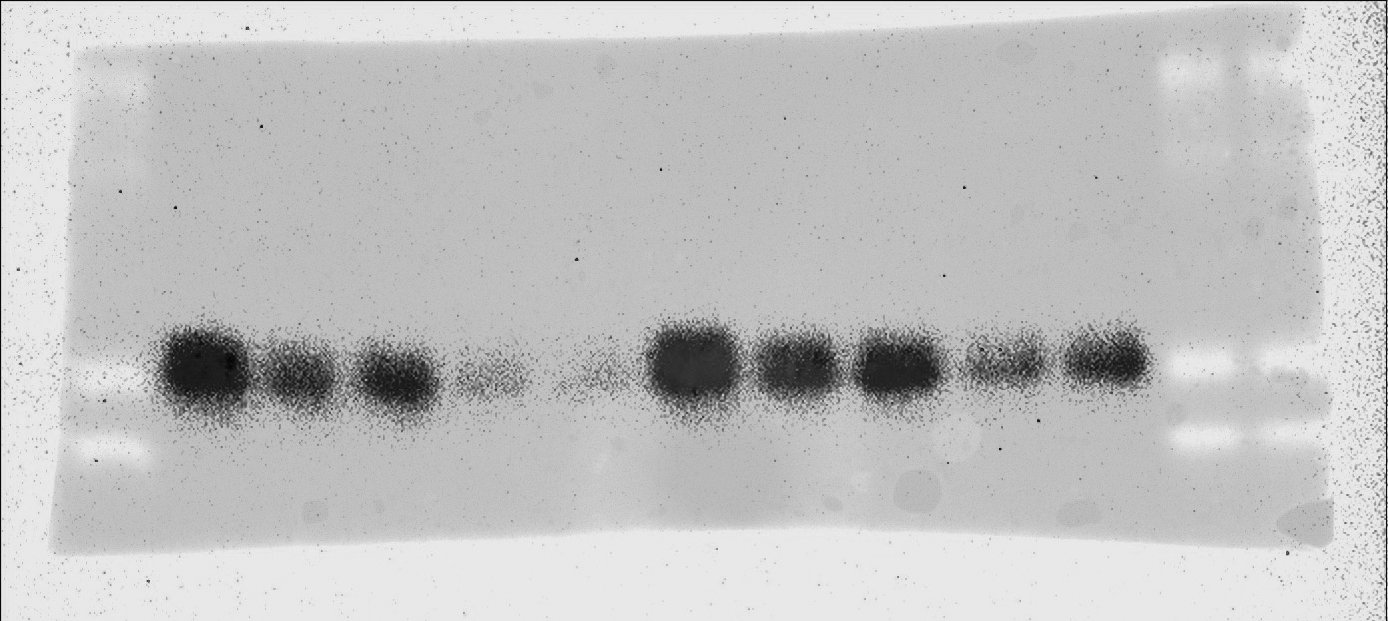

Supplement: Figure 2—figure supplement 2—source data 1. [file elife-79940-fig2-figsupp2-data1.zip › Fig 2-figure supplement 1-pS6 (s235:s236) unlabeled.tif .tiff]

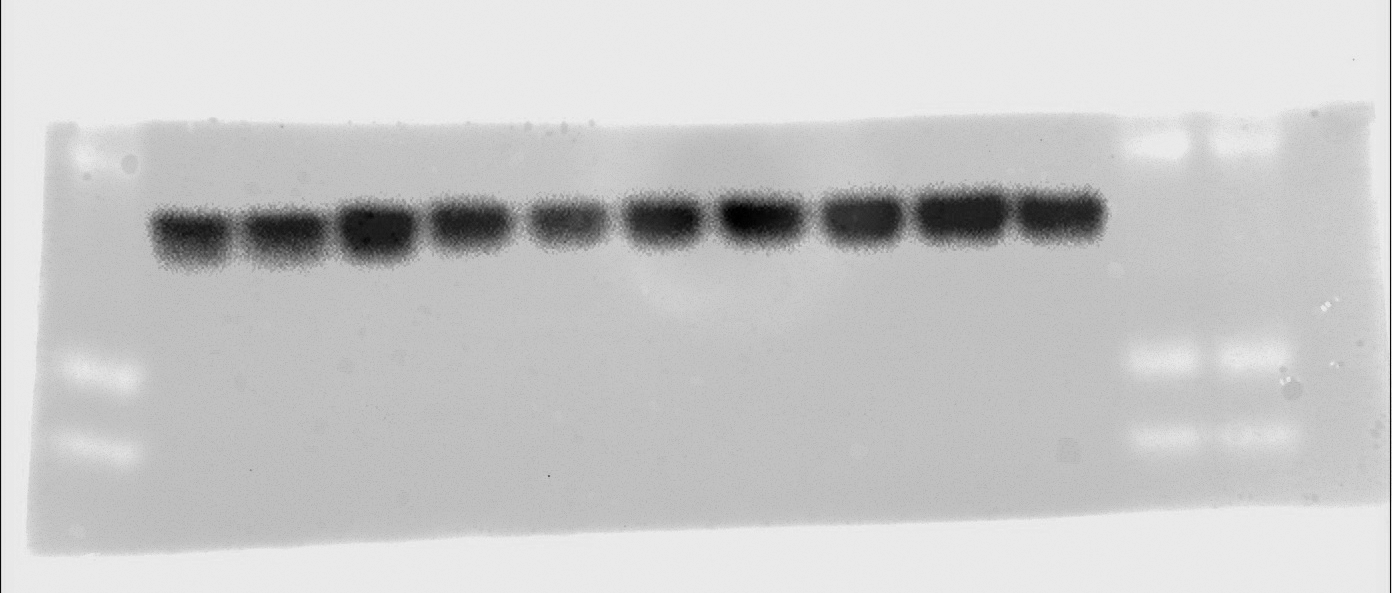

Supplement: Figure 2—figure supplement 2—source data 1. [file elife-79940-fig2-figsupp2-data1.zip › Fig 2-figure supplement 1-S6 unlabeled.tif]

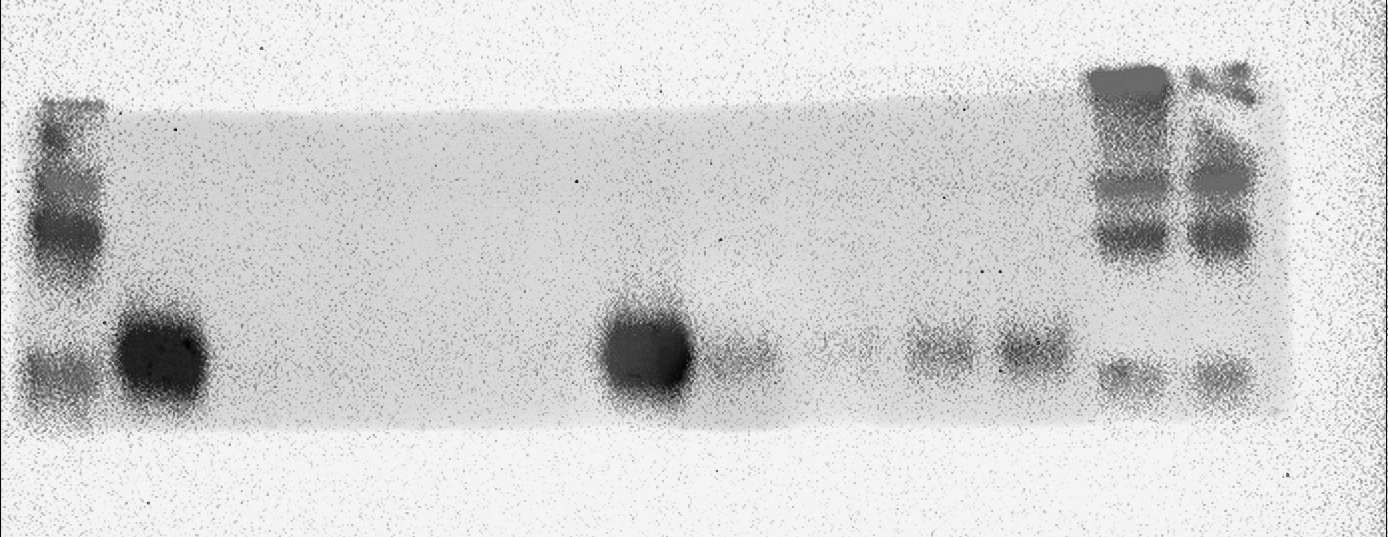

Supplement: Figure 2—figure supplement 2—source data 1. [file elife-79940-fig2-figsupp2-data1.zip › Fig 2-figure supplment 1-cMYC unlabeled.tif]

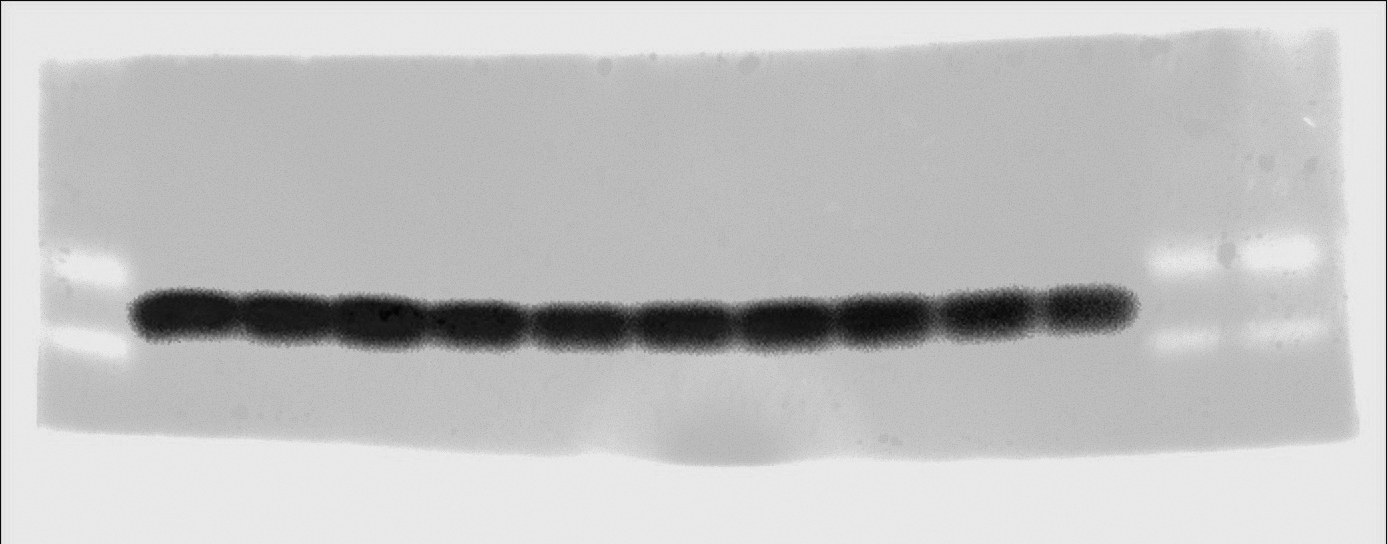

Supplement: Figure 2—figure supplement 2—source data 1. [file elife-79940-fig2-figsupp2-data1.zip › Fig 2-figure supplement 1-H3 unlabeled.tif]

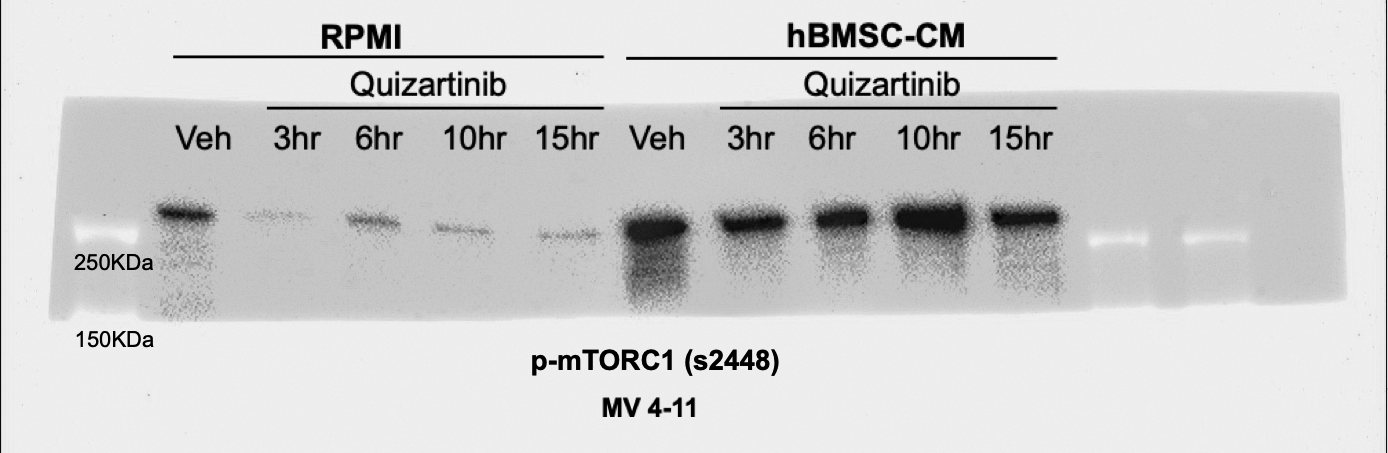

Supplement: Figure 2—figure supplement 2—source data 2. [file elife-79940-fig2-figsupp2-data2.zip › Fig 2-figure supplement 1- pMTORC1 (s2448) labeled .tiff]

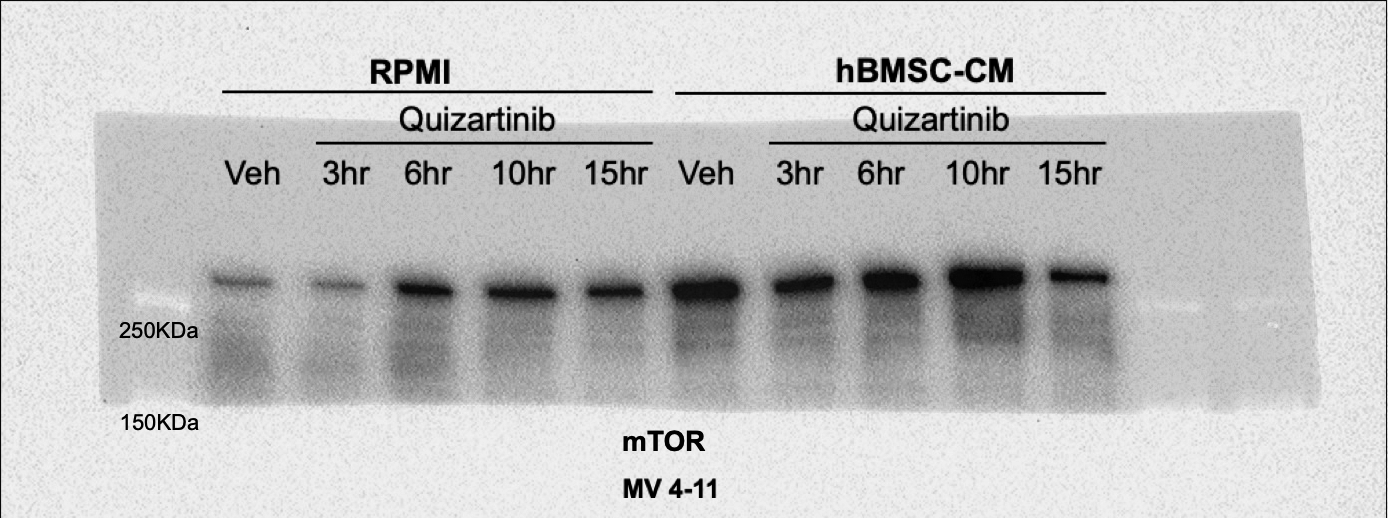

Supplement: Figure 2—figure supplement 2—source data 2. [file elife-79940-fig2-figsupp2-data2.zip › Fig 2-figure supplement 1-mTOR labeled.tiff]

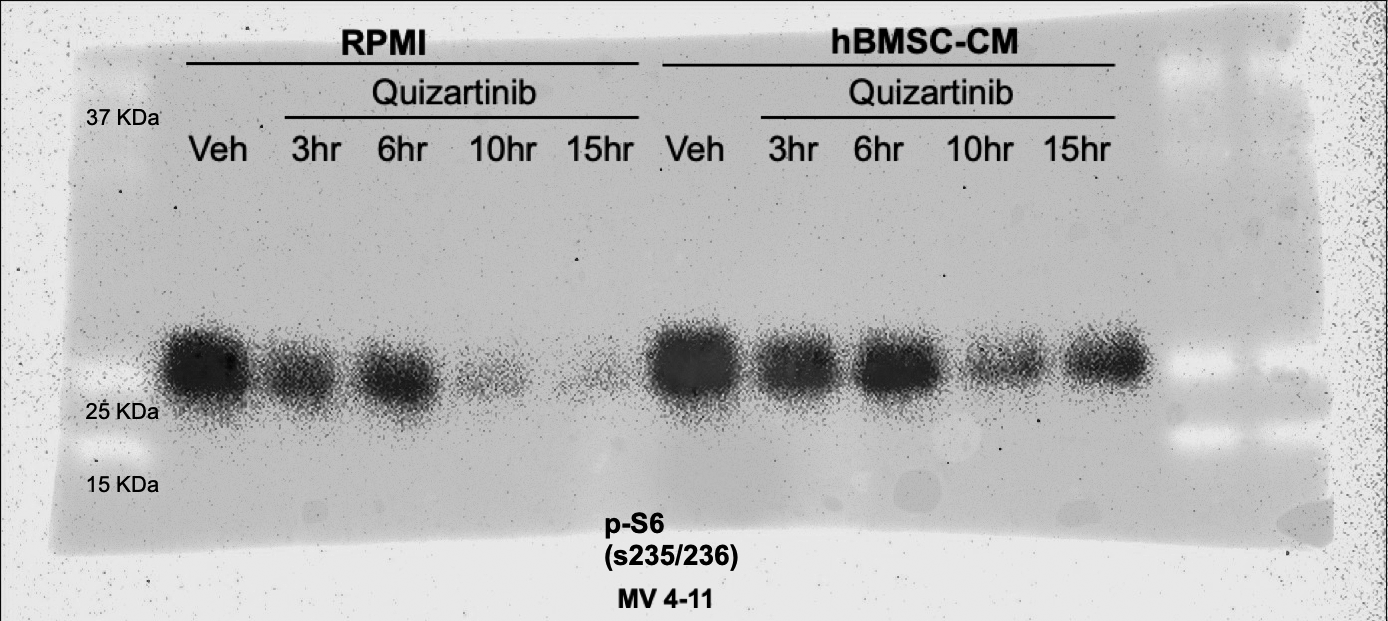

Supplement: Figure 2—figure supplement 2—source data 2. [file elife-79940-fig2-figsupp2-data2.zip › Fig 2-figure supplement 1-pS6 (s235:s236) labeled.tif]

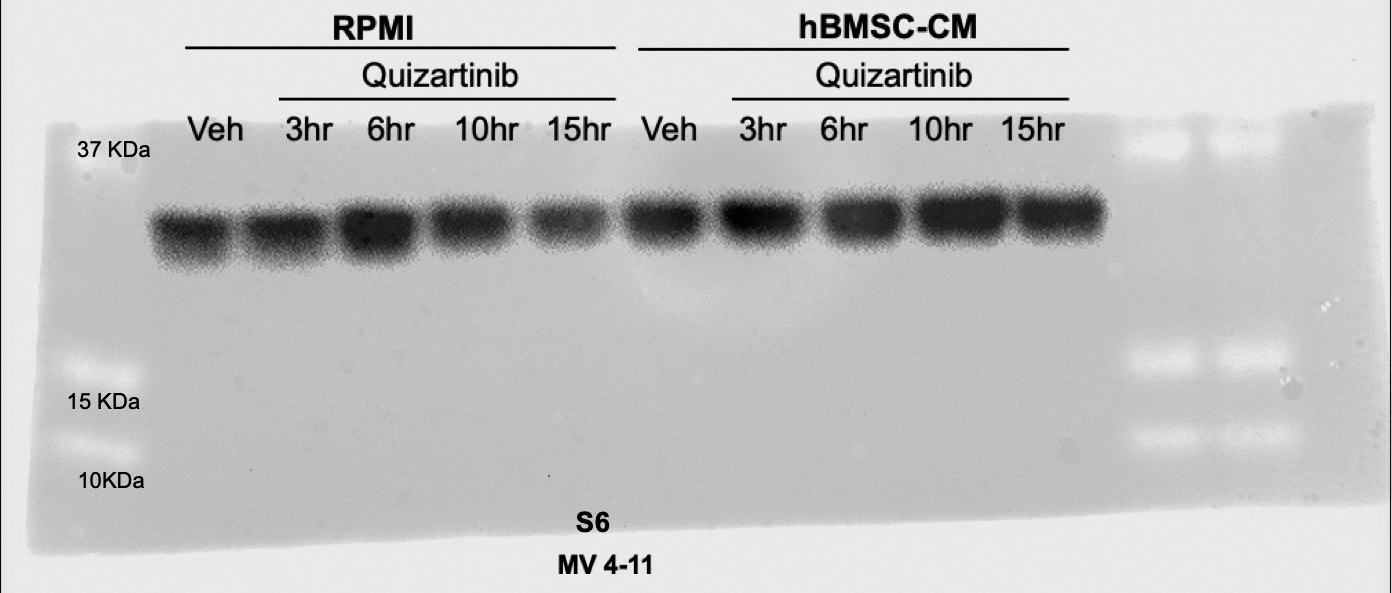

Supplement: Figure 2—figure supplement 2—source data 2. [file elife-79940-fig2-figsupp2-data2.zip › Fig 2-figure supplement 1-S6 labeled .tiff]

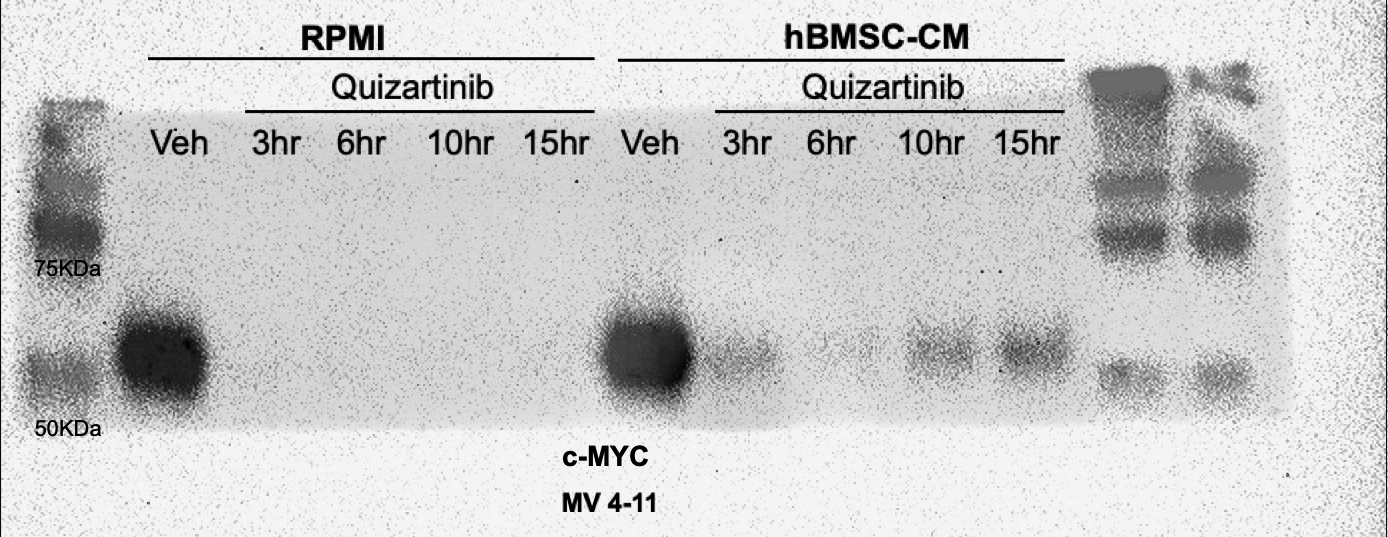

Supplement: Figure 2—figure supplement 2—source data 2. [file elife-79940-fig2-figsupp2-data2.zip › Fig 2-figure supplment 1-cMYC labeled .tiff]

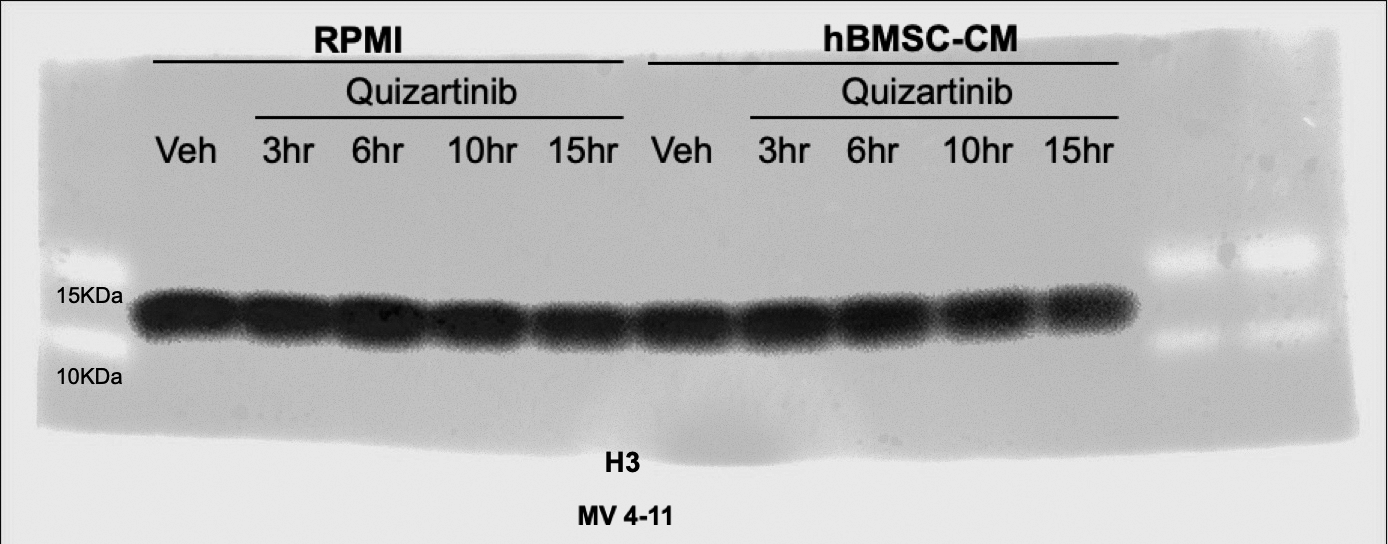

Supplement: Figure 2—figure supplement 2—source data 2. [file elife-79940-fig2-figsupp2-data2.zip › Fig 2-figure supplement 1-H3 labeled .tiff]

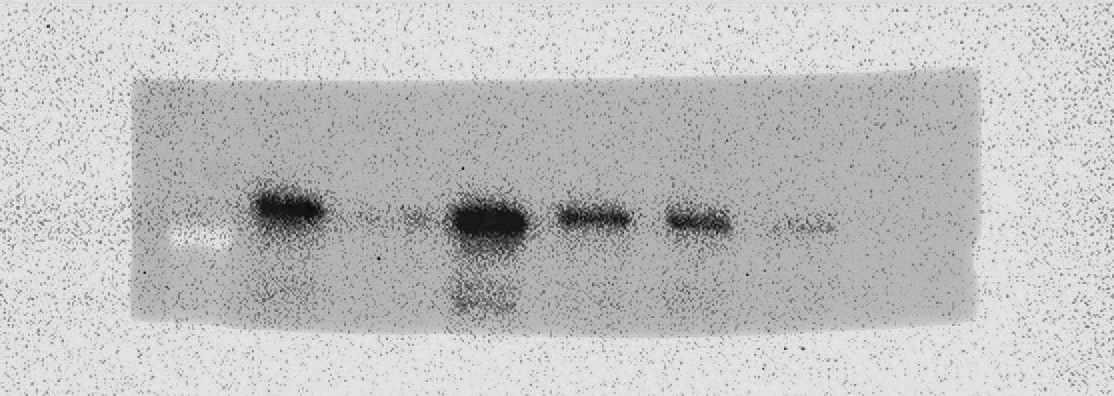

Supplement: Figure 4—source data 1. [file elife-79940-fig4-data1.zip › 4B-pMTORC1 (s2448) unlabeled.tif]

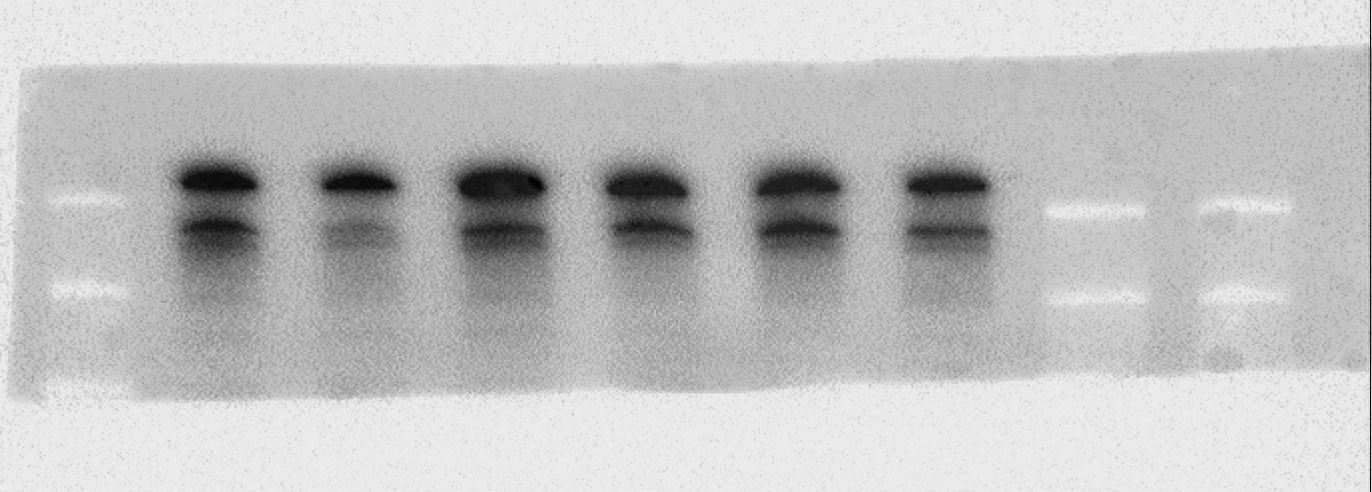

Supplement: Figure 4—source data 1. [file elife-79940-fig4-data1.zip › 4B-mTOR unlabeled.tif]

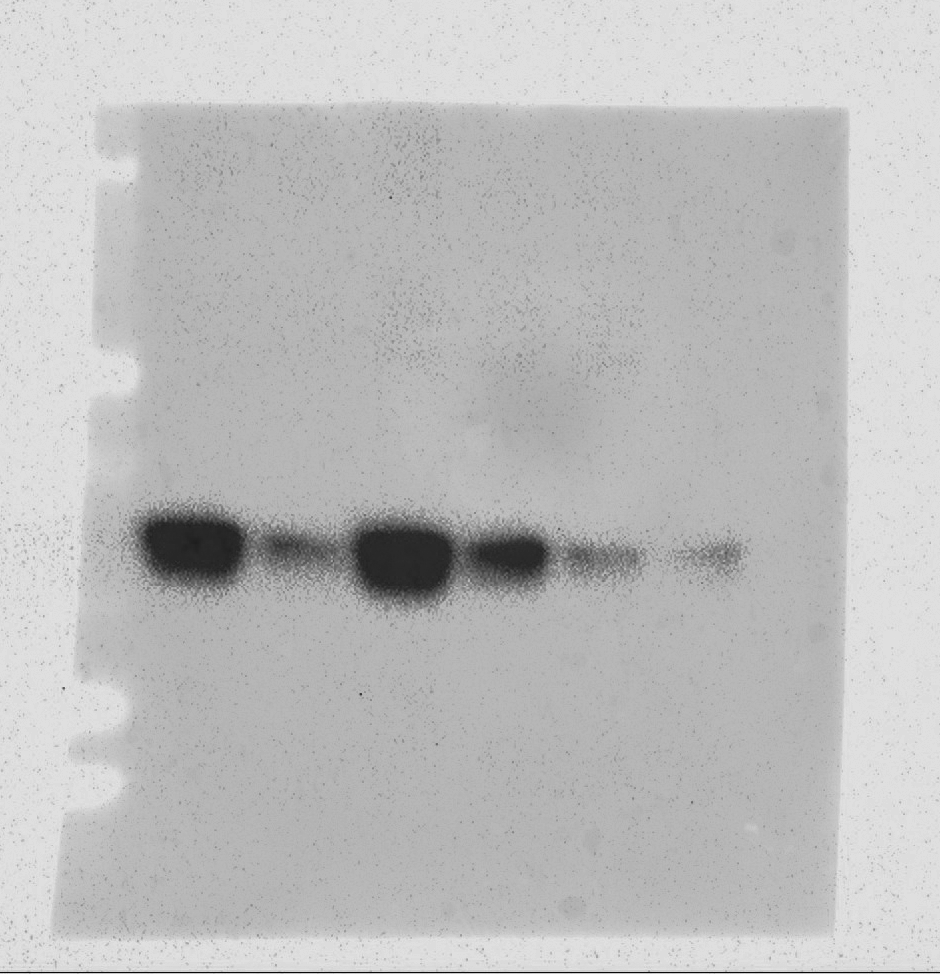

Supplement: Figure 4—source data 1. [file elife-79940-fig4-data1.zip › 4B-pS6(s240:244) unlabeled.tif]

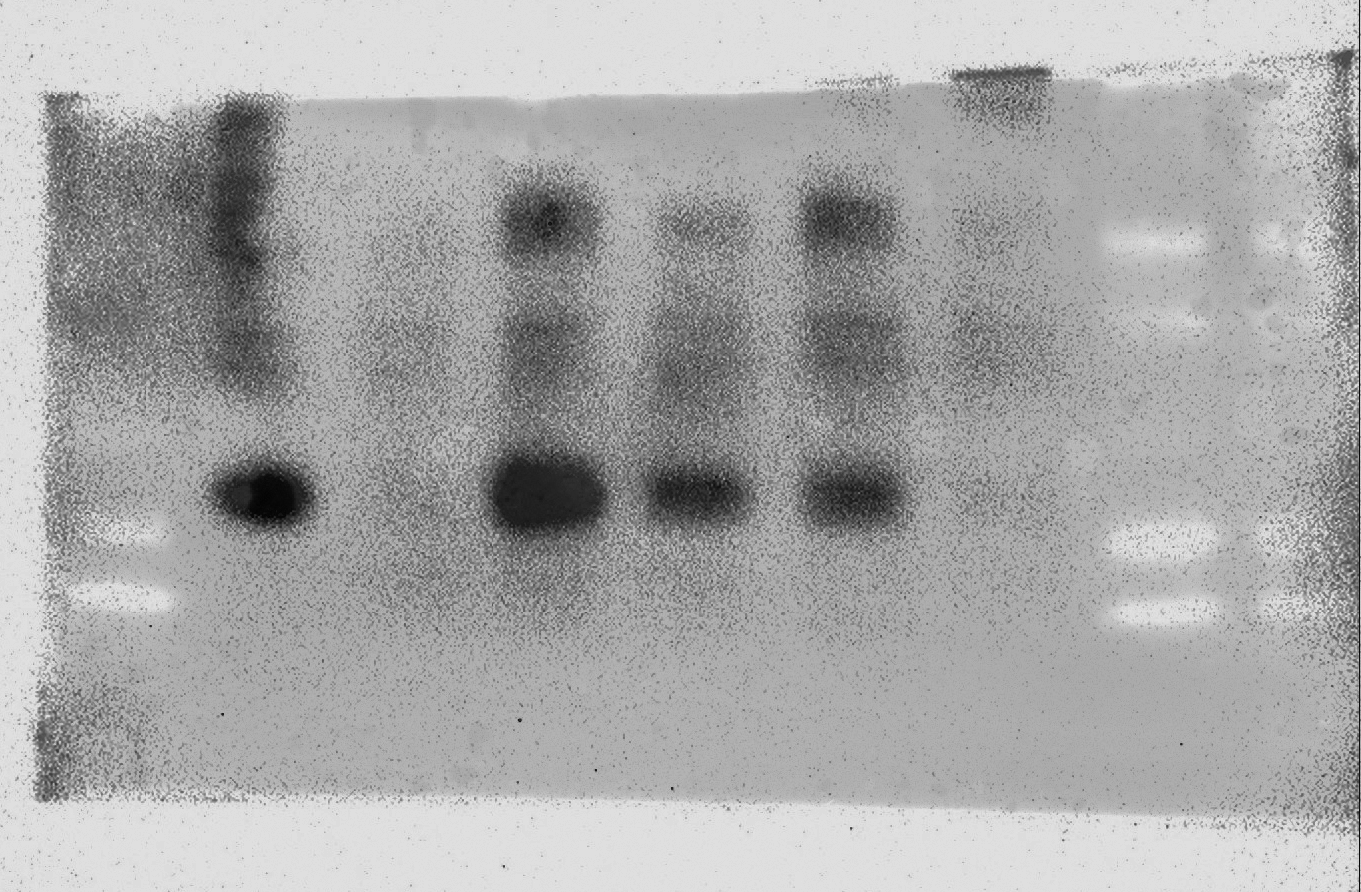

Supplement: Figure 4—source data 1. [file elife-79940-fig4-data1.zip › 4B-p4EBP1 (s65) unlabeled.tif]

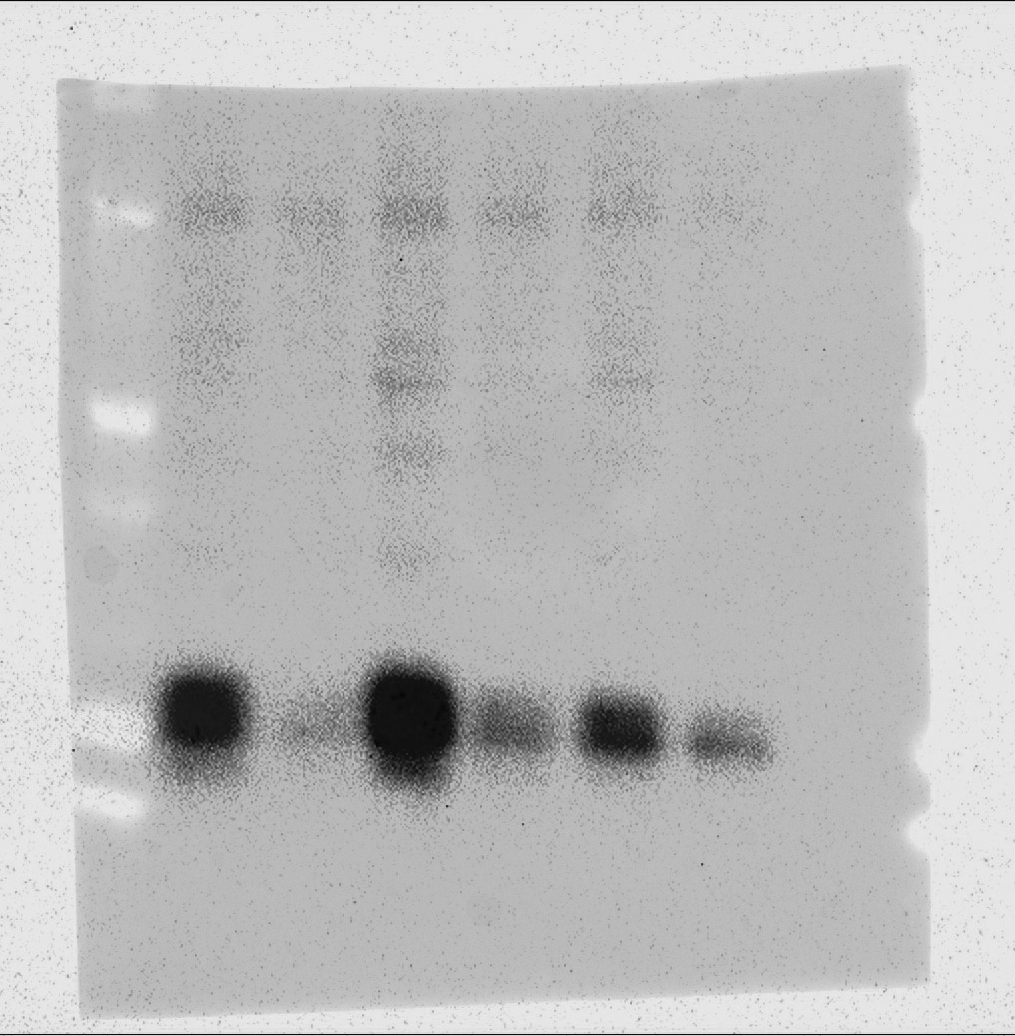

Supplement: Figure 4—source data 1. [file elife-79940-fig4-data1.zip › 4B-p4EBP1 (T37:46) unlabeled.tif]

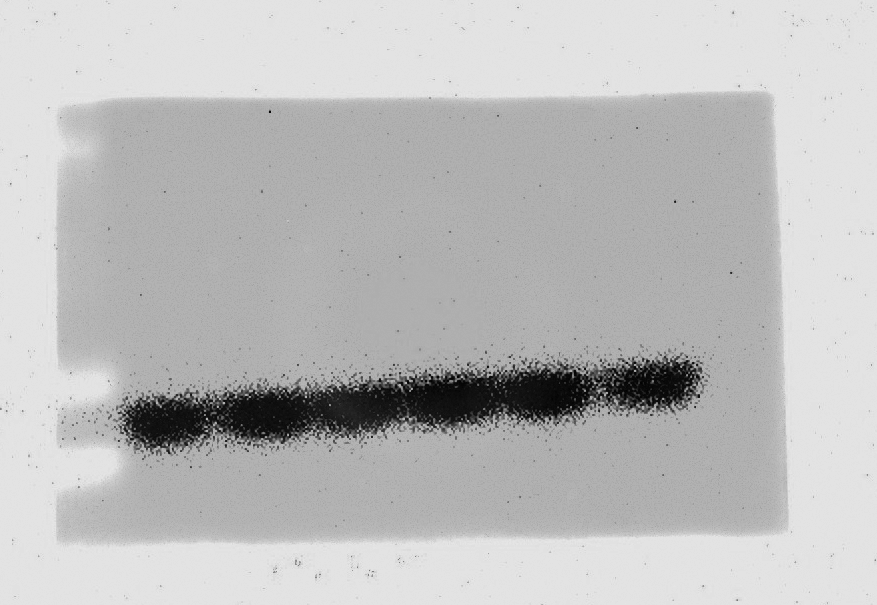

Supplement: Figure 4—source data 1. [file elife-79940-fig4-data1.zip › 4B-H3 unlabeled.tif]

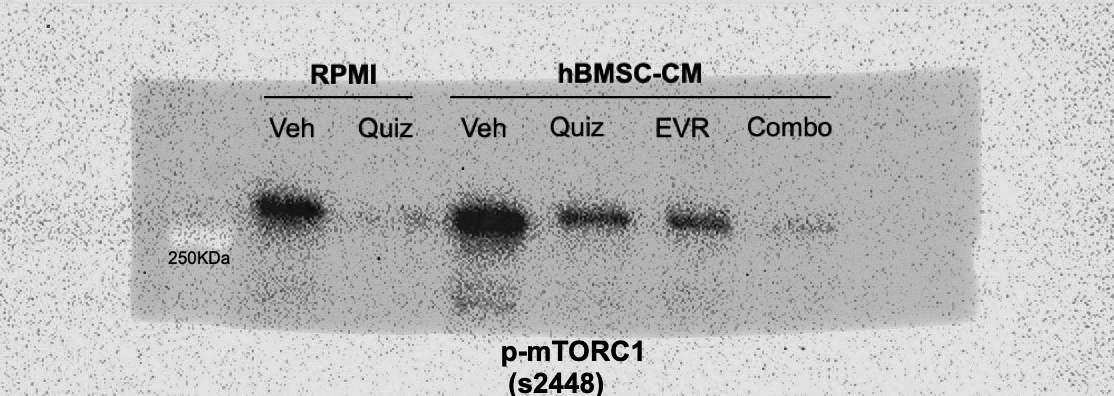

Supplement: Figure 4—source data 2. [file elife-79940-fig4-data2.zip › 4B-pMTORC1 (s2448) labeled.tiff]

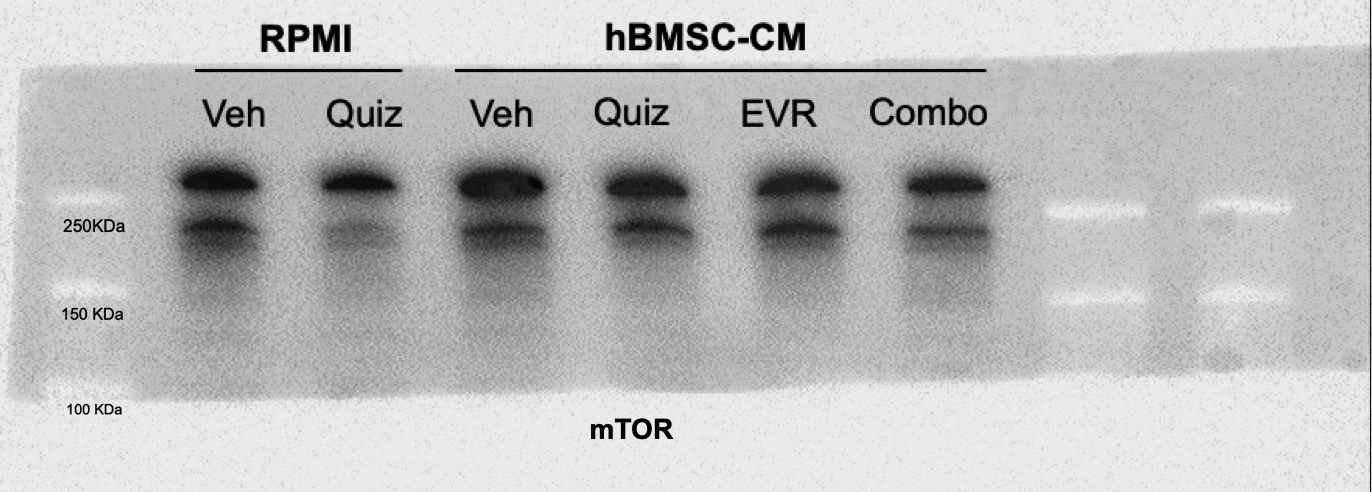

Supplement: Figure 4—source data 2. [file elife-79940-fig4-data2.zip › 4B-mTOR labeled .tiff]

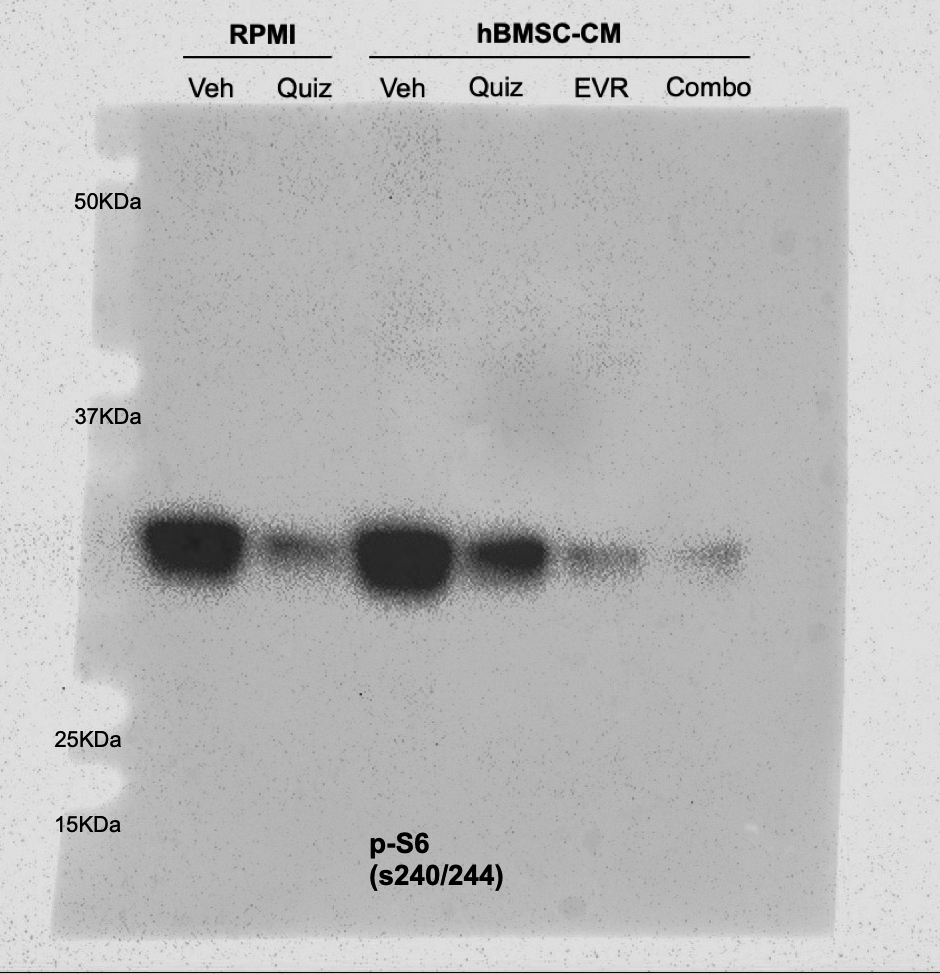

Supplement: Figure 4—source data 2. [file elife-79940-fig4-data2.zip › 4B-pS6(s240:244) labeled.tiff]

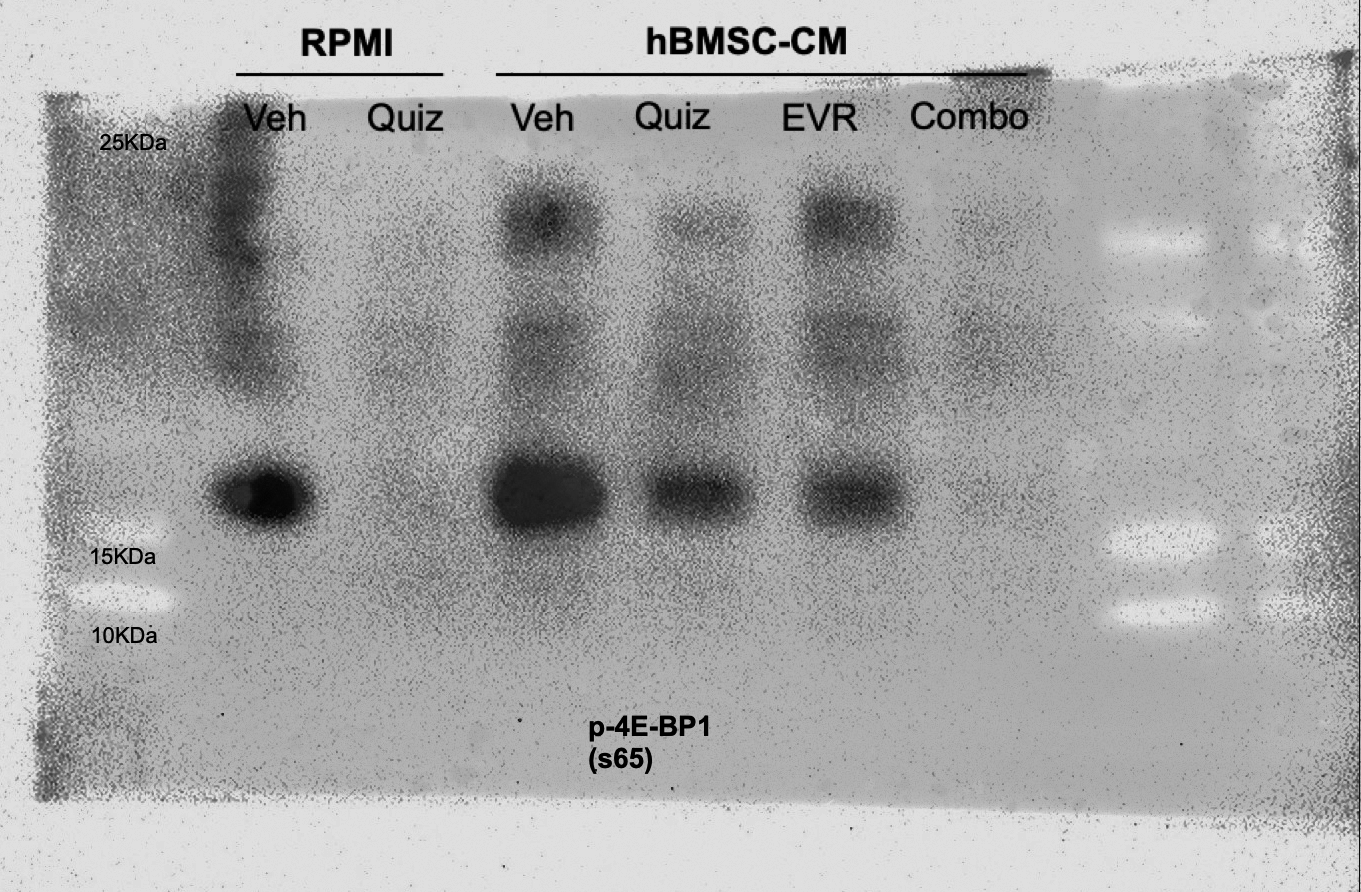

Supplement: Figure 4—source data 2. [file elife-79940-fig4-data2.zip › 4B-p4EBP1 (s65) labeled .tiff]

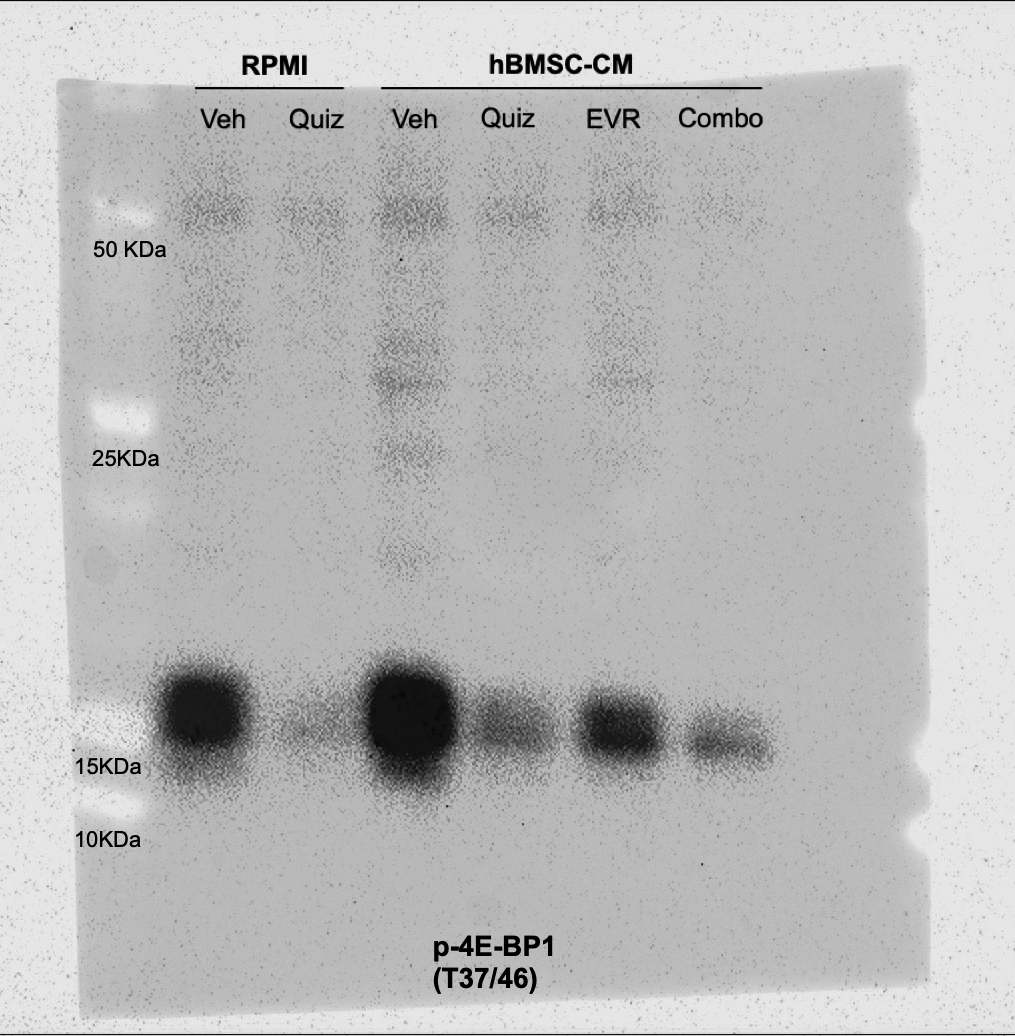

Supplement: Figure 4—source data 2. [file elife-79940-fig4-data2.zip › 4B-p4EBP1 (T37:46) labeled.tif]

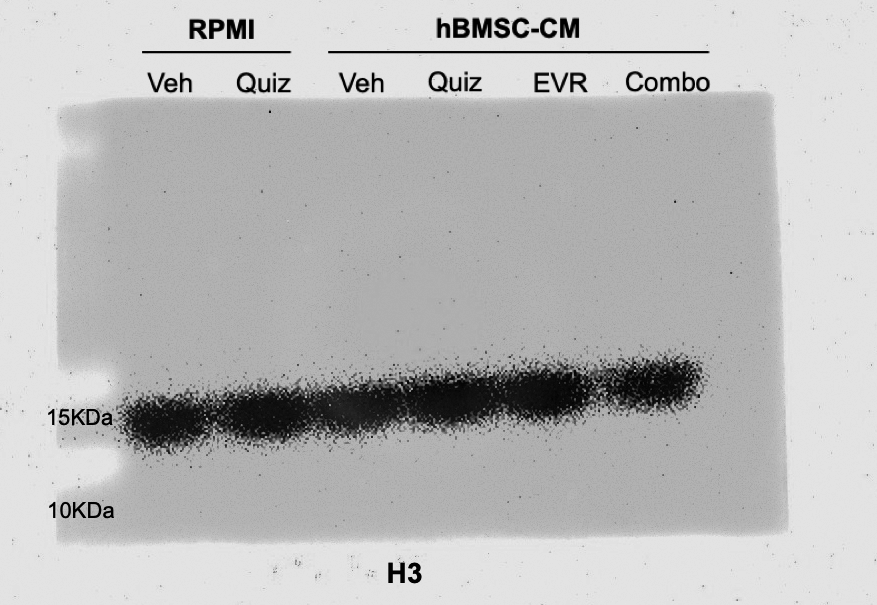

Supplement: Figure 4—source data 2. [file elife-79940-fig4-data2.zip › 4B-H3 labeled.tiff]

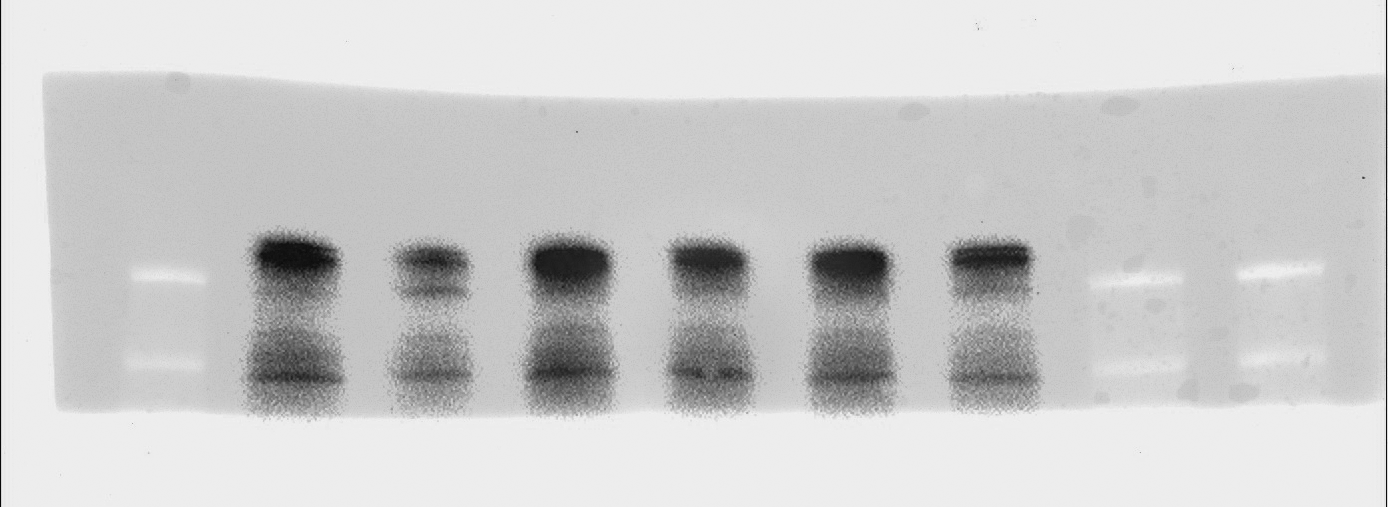

Supplement: Figure 7—source data 1. [file elife-79940-fig7-data1.zip › 7A-ATM unlabeled.tif]

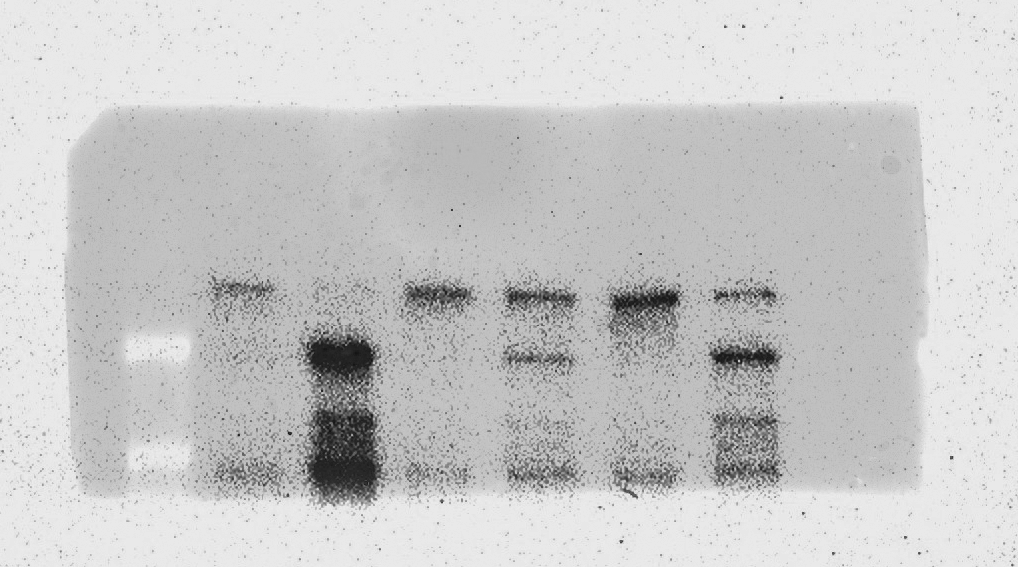

Supplement: Figure 7—source data 1. [file elife-79940-fig7-data1.zip › 7A-pATM (s1981) unlabeled.tif]

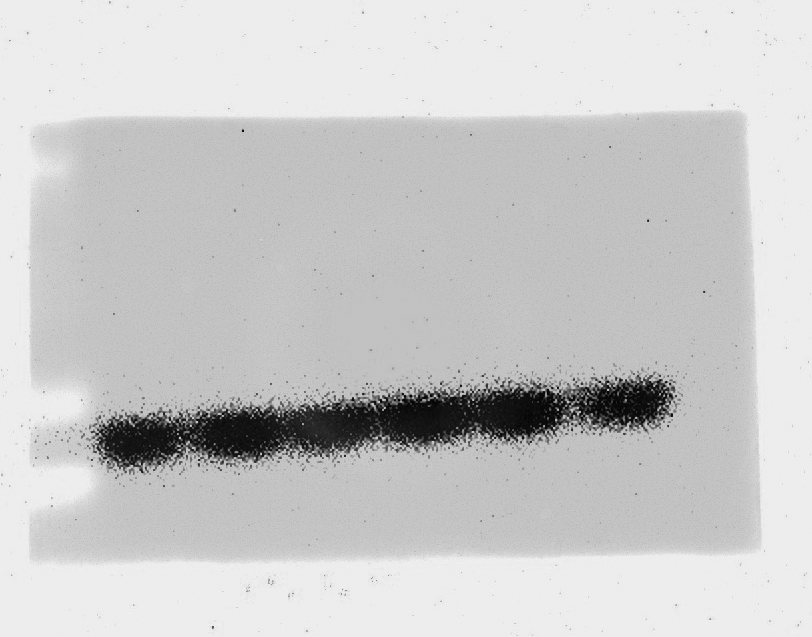

Supplement: Figure 7—source data 1. [file elife-79940-fig7-data1.zip › 7A-H3 unlabeled.tif]

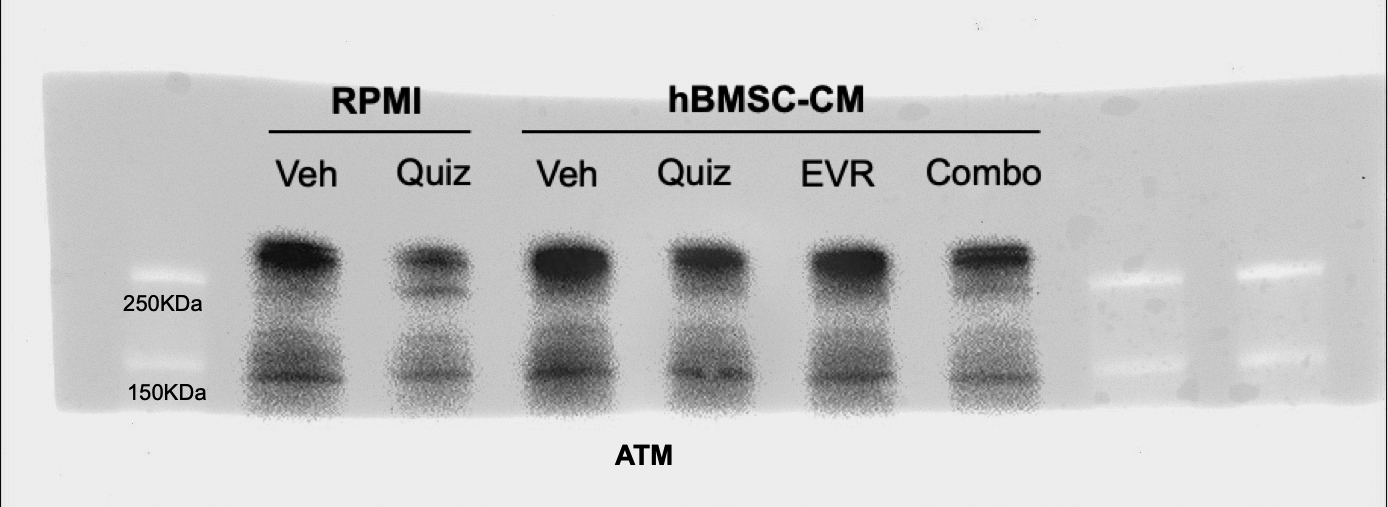

Supplement: Figure 7—source data 2. [file elife-79940-fig7-data2.zip › 7A-ATM labeled.tiff]

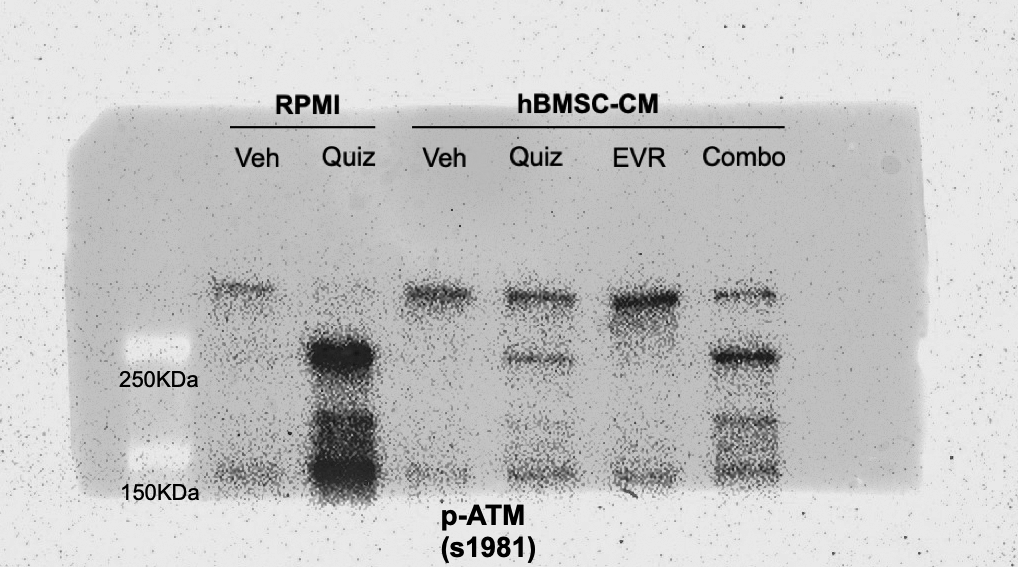

Supplement: Figure 7—source data 2. [file elife-79940-fig7-data2.zip › 7A-pATM (s1981) labeled .tiff]

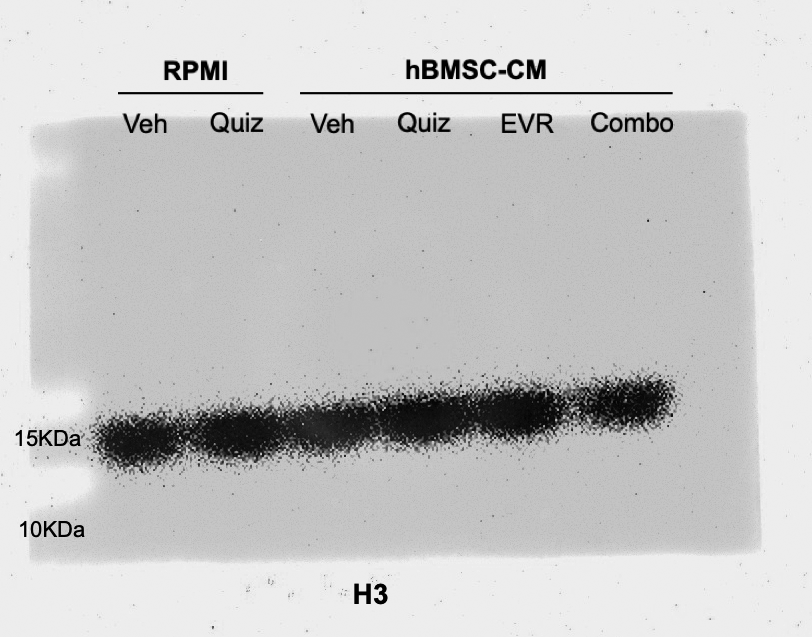

Supplement: Figure 7—source data 2. [file elife-79940-fig7-data2.zip › 7A-H3 labeled .tiff]

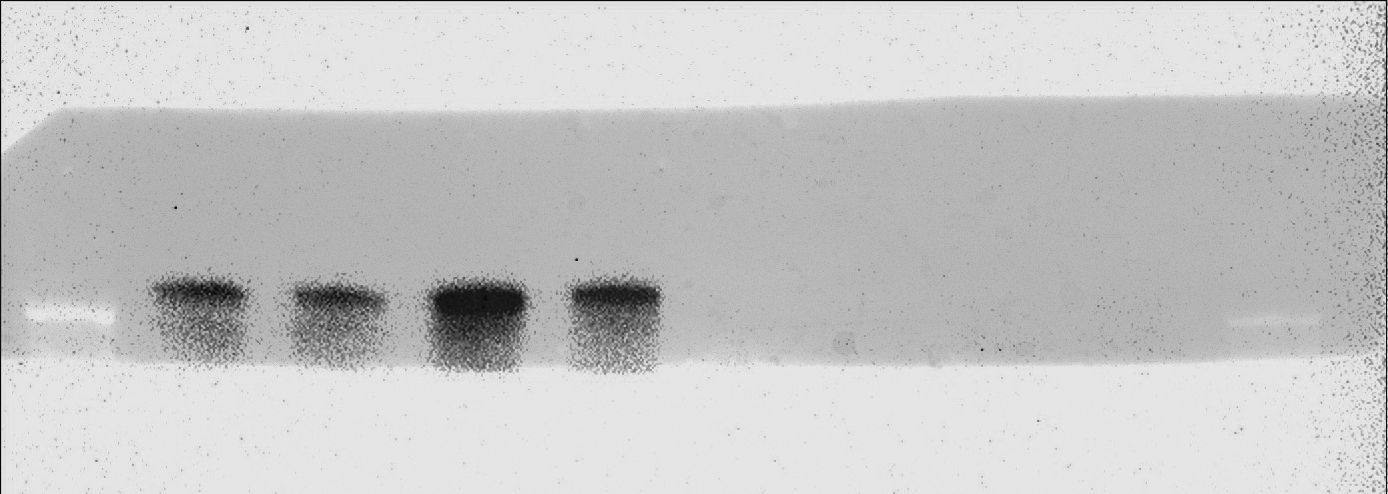

Supplement: Figure 7—source data 3. [file elife-79940-fig7-data3.zip › 7C-ATM unlabeled.tif]

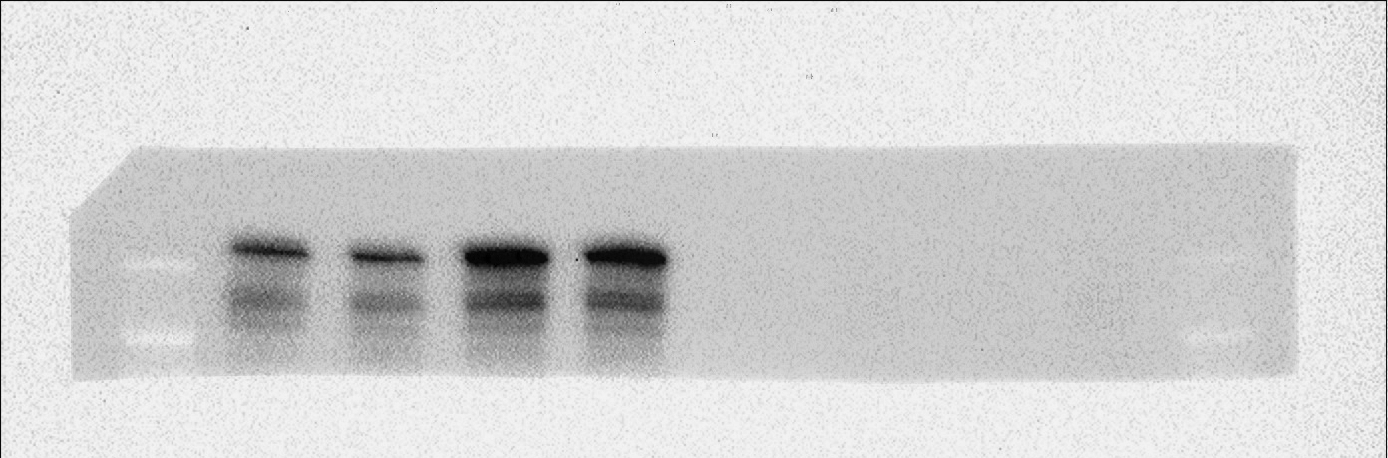

Supplement: Figure 7—source data 3. [file elife-79940-fig7-data3.zip › 7C-mTOR unlabeled.tif]

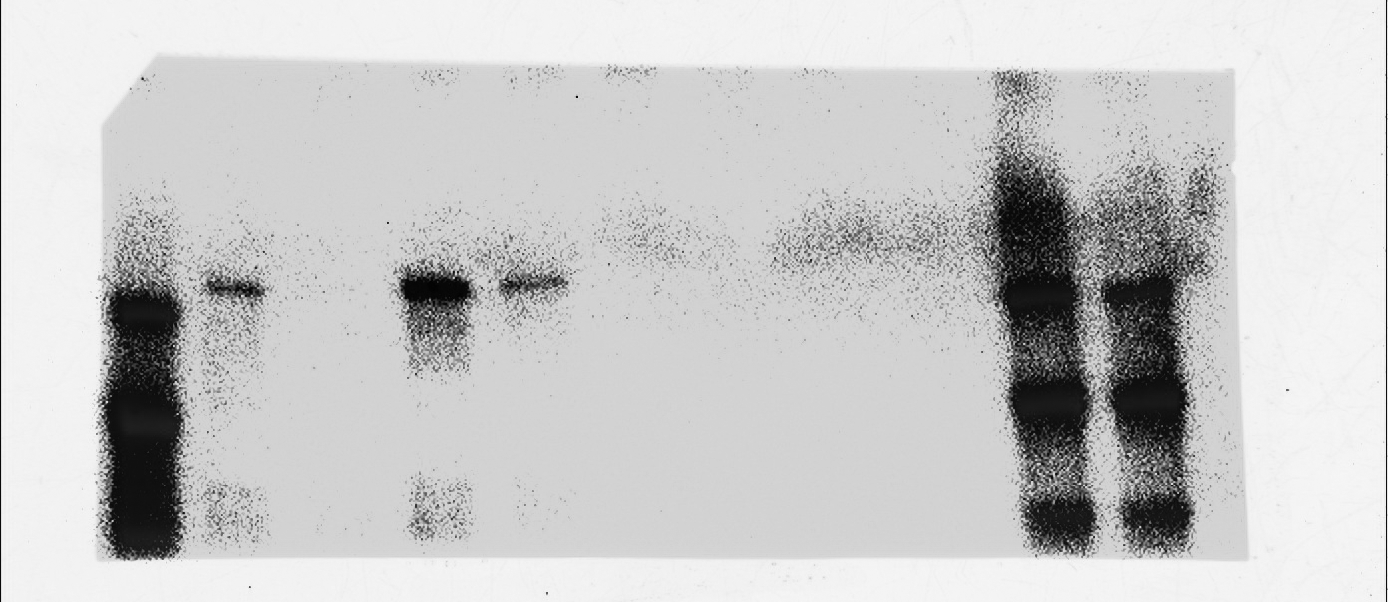

Supplement: Figure 7—source data 3. [file elife-79940-fig7-data3.zip › 7C-pMTOR (s2448) unlabeled.tif]

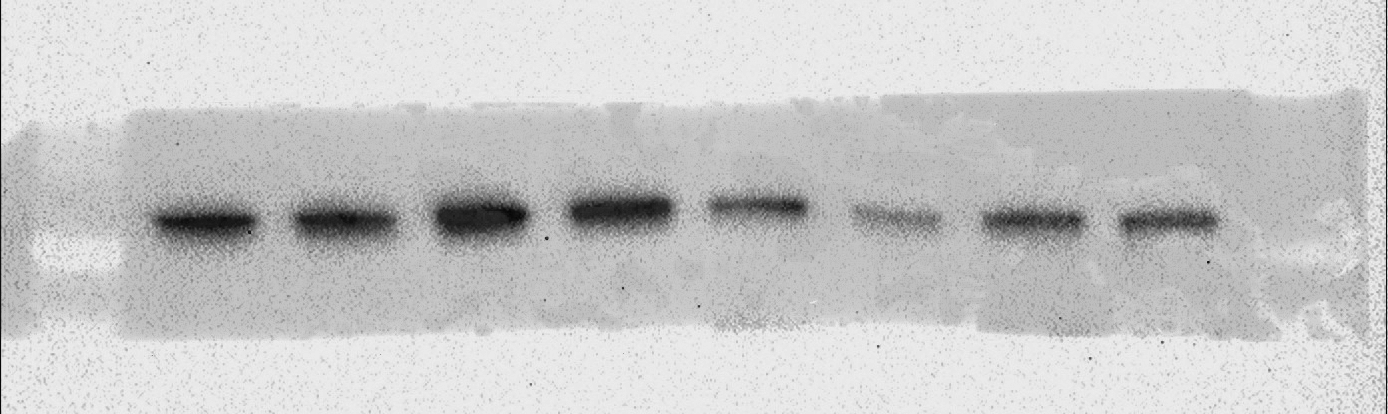

Supplement: Figure 7—source data 3. [file elife-79940-fig7-data3.zip › 7C-AKT unlabeled.tif]

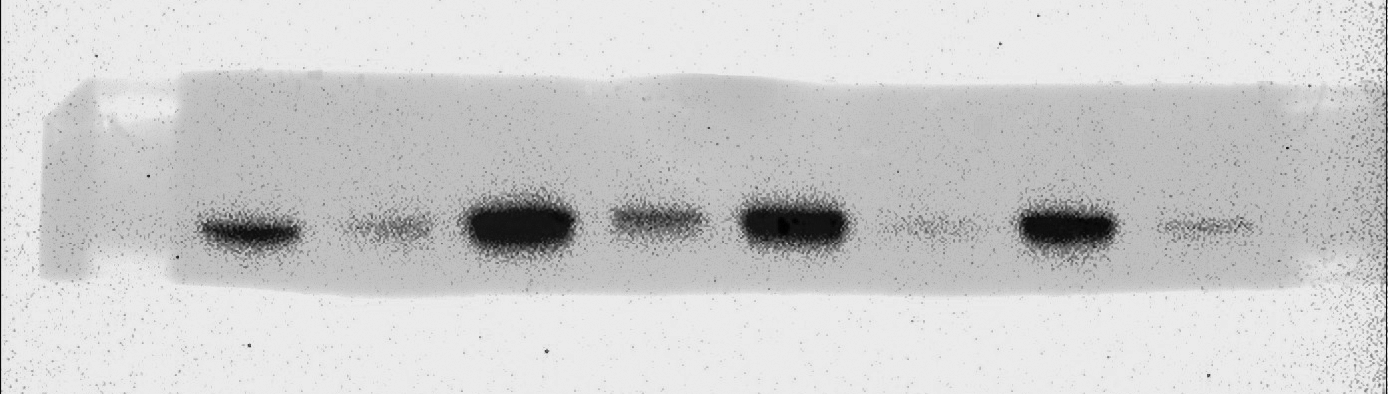

Supplement: Figure 7—source data 3. [file elife-79940-fig7-data3.zip › 7C-pAKT (s473) unlabeled.tif]

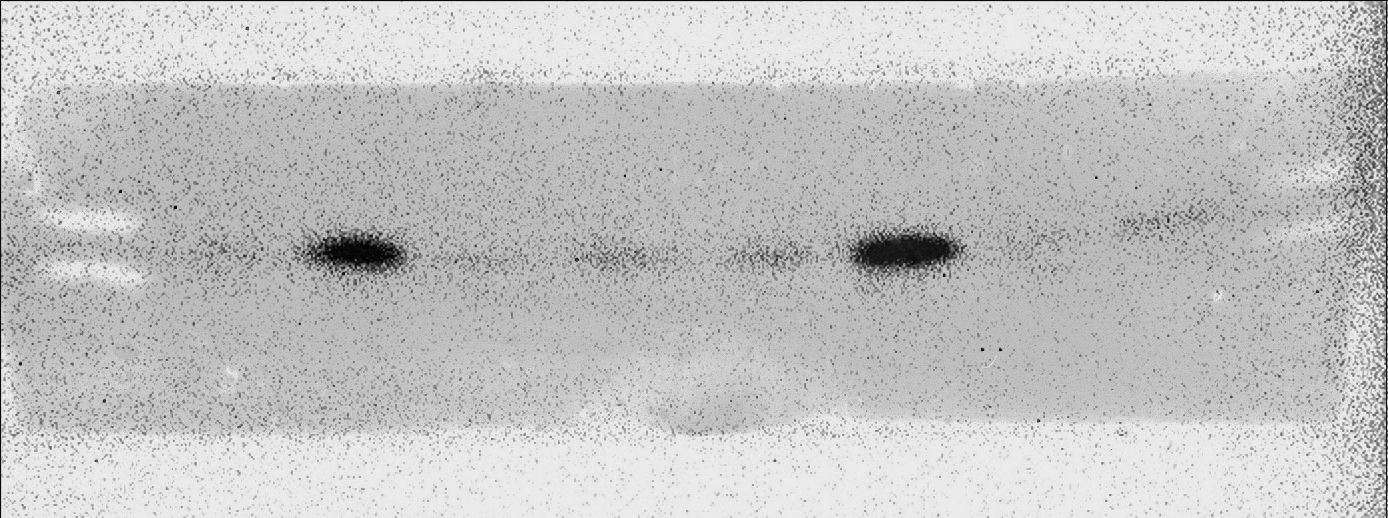

Supplement: Figure 7—source data 3. [file elife-79940-fig7-data3.zip › 7C-pH2AX (s139) unlabeled.tif]

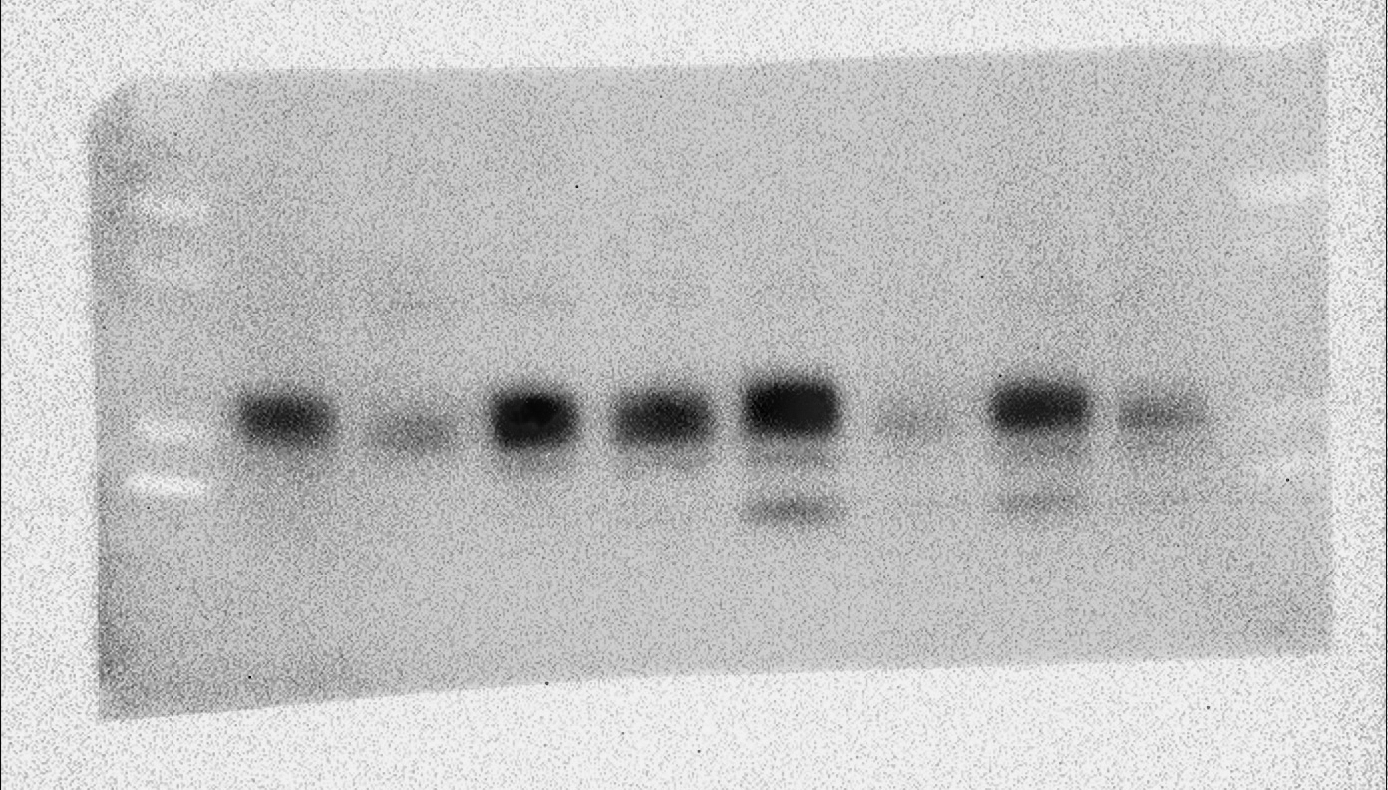

Supplement: Figure 7—source data 3. [file elife-79940-fig7-data3.zip › 7C-p4EBP1 (T37:46) unlabeled.tif]

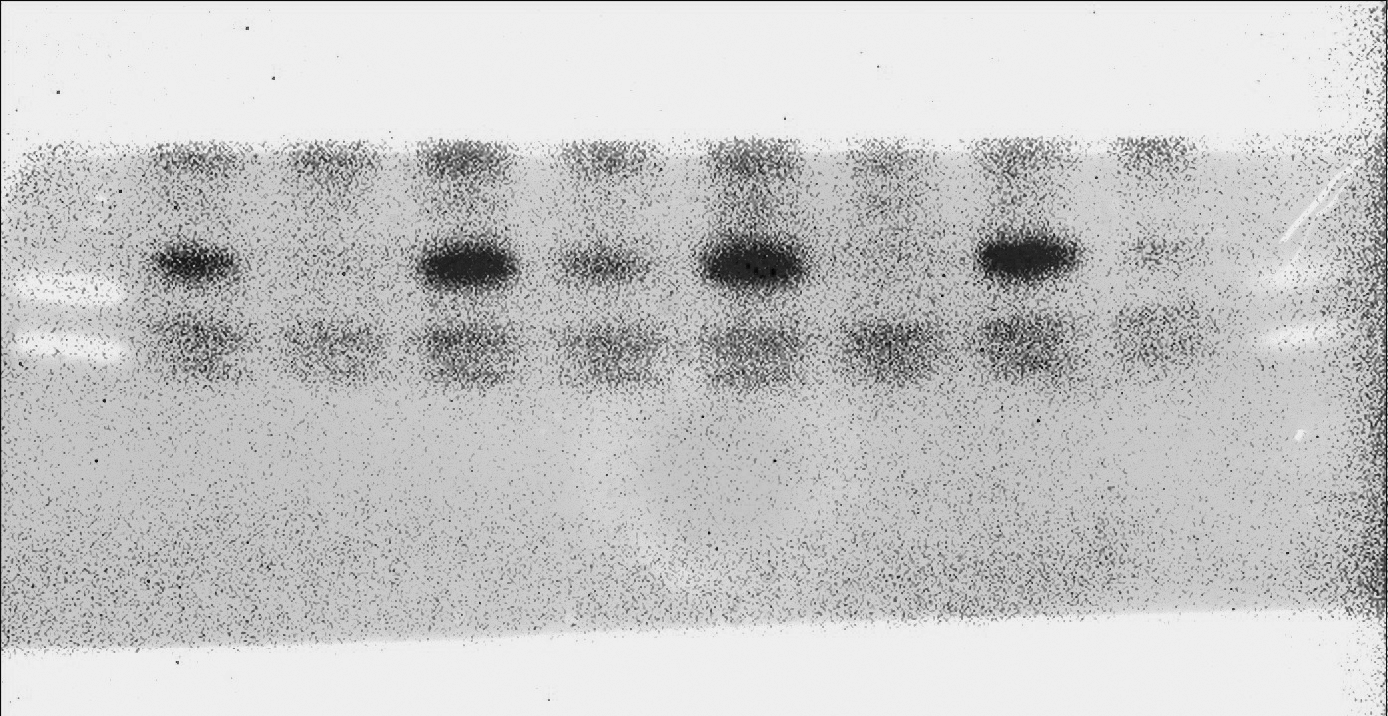

Supplement: Figure 7—source data 3. [file elife-79940-fig7-data3.zip › 7C-p4EBP1(s65) unlabeled.tif]

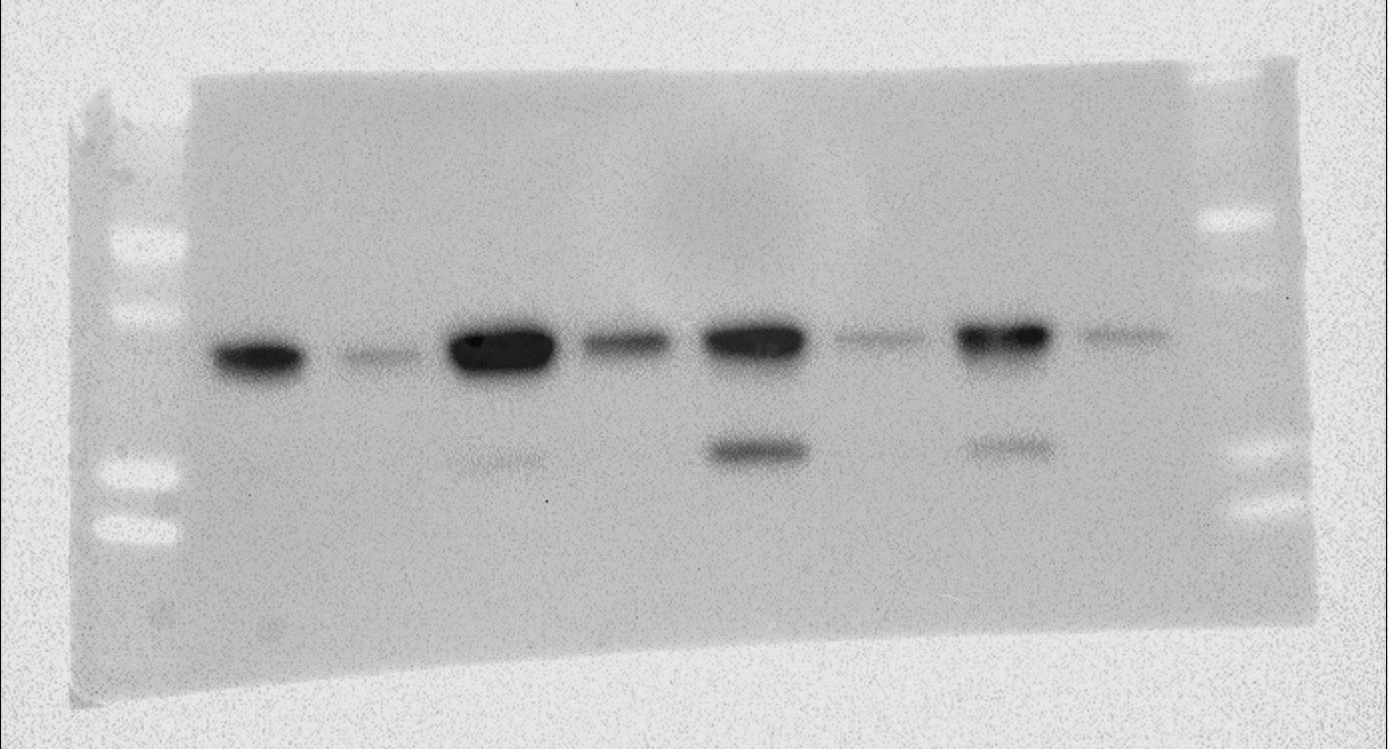

Supplement: Figure 7—source data 3. [file elife-79940-fig7-data3.zip › 7C-pS6 (s240:244) unlabeled.tif]

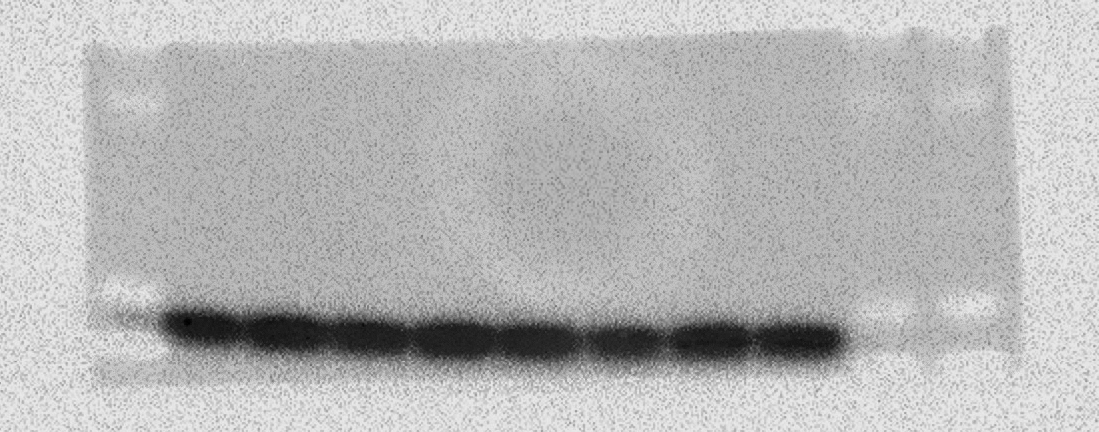

Supplement: Figure 7—source data 3. [file elife-79940-fig7-data3.zip › 7C-H3 unlabeled.tif]

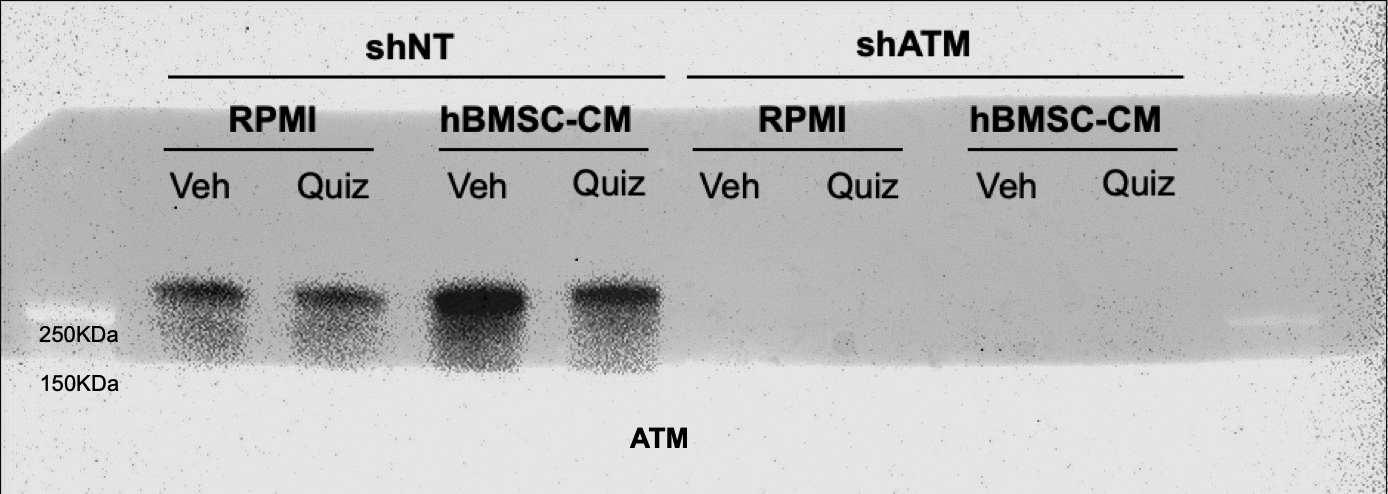

Supplement: Figure 7—source data 4. [file elife-79940-fig7-data4.zip › 7C-ATM labeled .tiff]

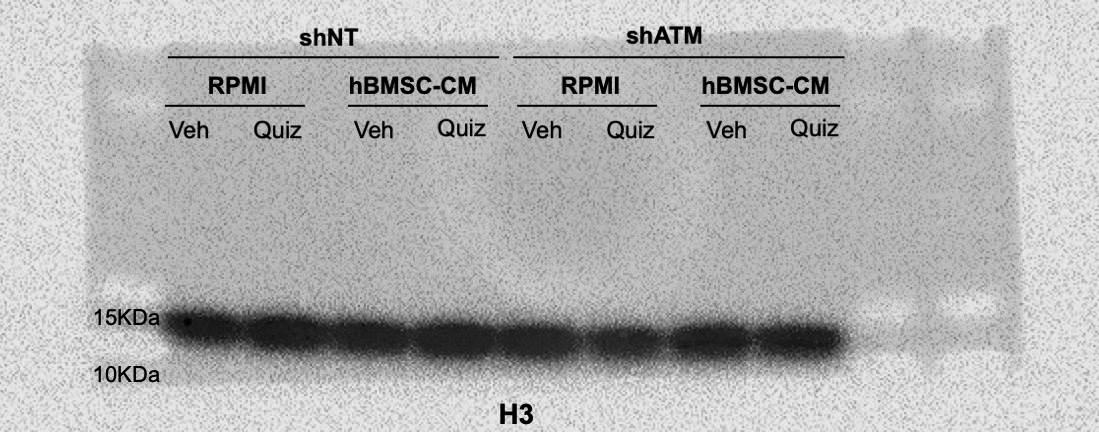

Supplement: Figure 7—source data 4. [file elife-79940-fig7-data4.zip › 7C-H3 labeled .tiff]

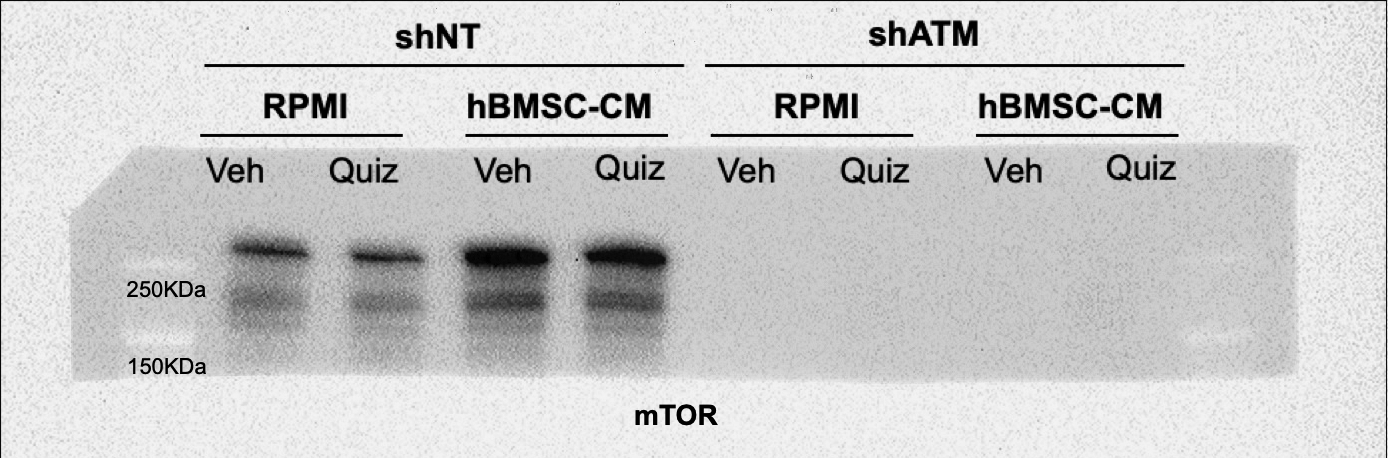

Supplement: Figure 7—source data 4. [file elife-79940-fig7-data4.zip › 7C-mTOR labeled.tiff]

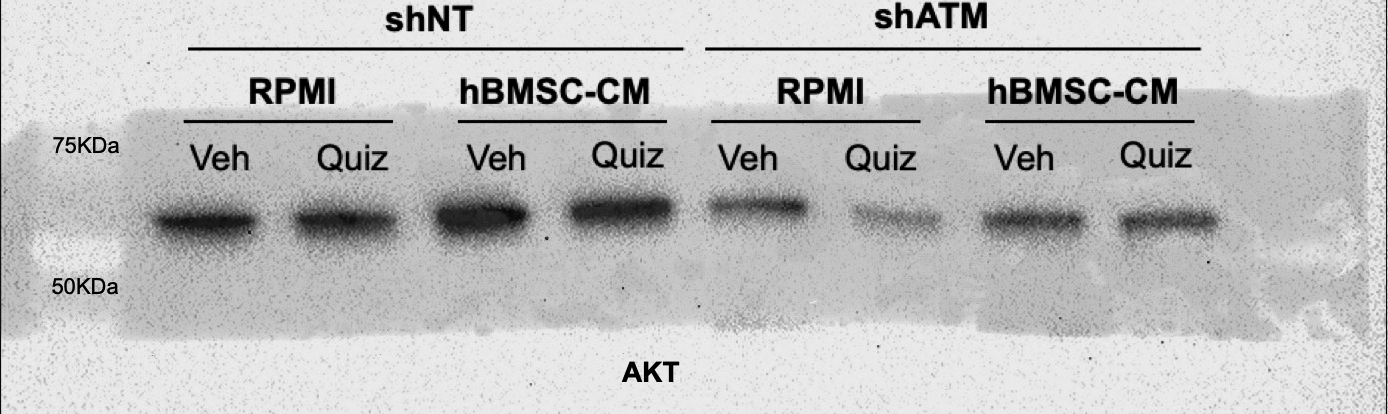

Supplement: Figure 7—source data 4. [file elife-79940-fig7-data4.zip › 7C-AKT labeled .tiff]

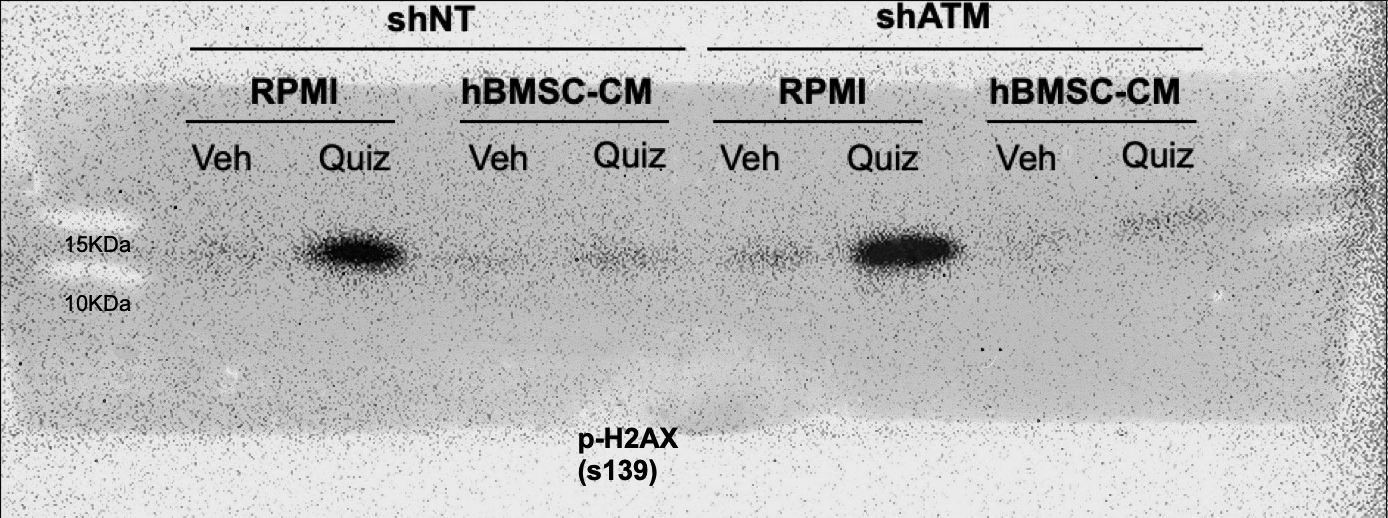

Supplement: Figure 7—source data 4. [file elife-79940-fig7-data4.zip › 7C-pH2AX (s139) labeled.tiff]

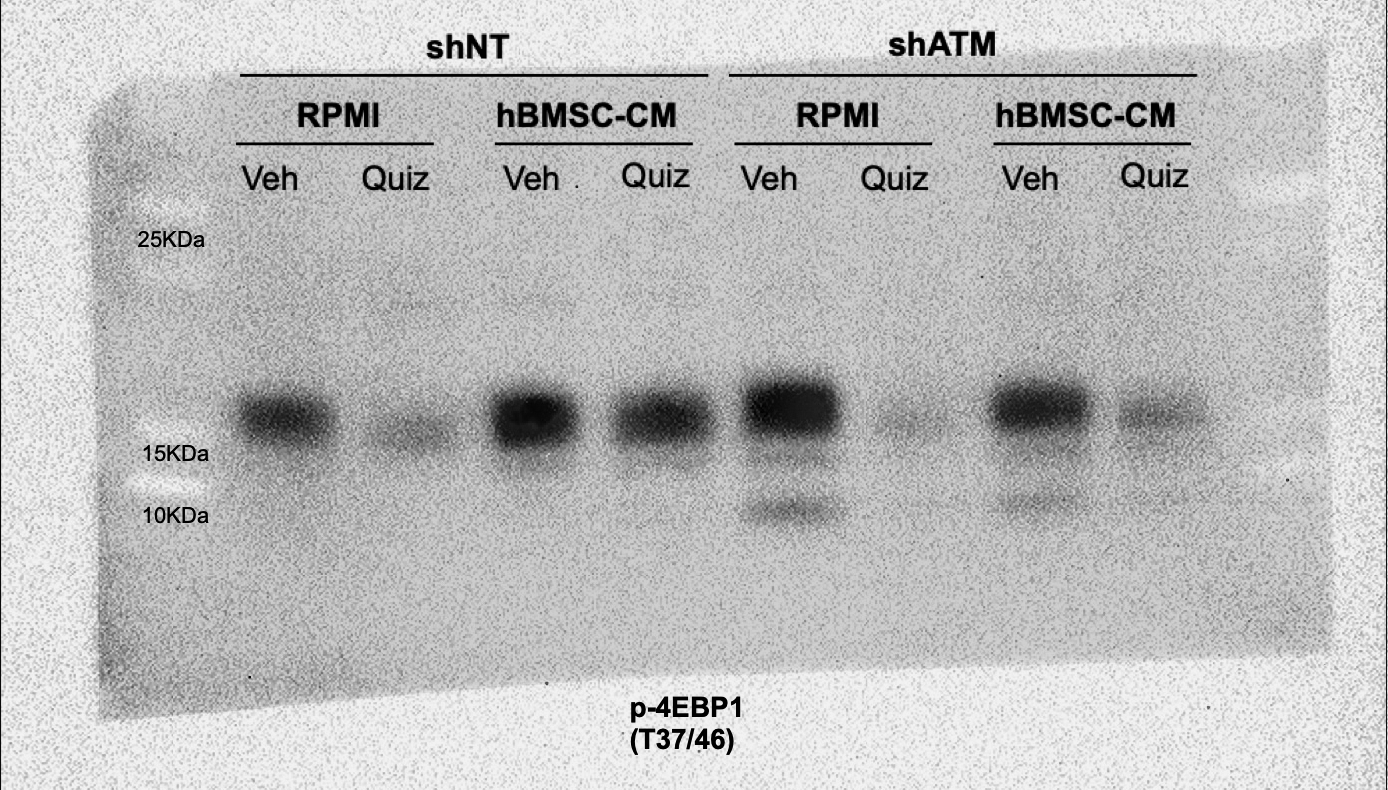

Supplement: Figure 7—source data 4. [file elife-79940-fig7-data4.zip › 7C-p4EBP1 (T37:46) labeled.tiff]

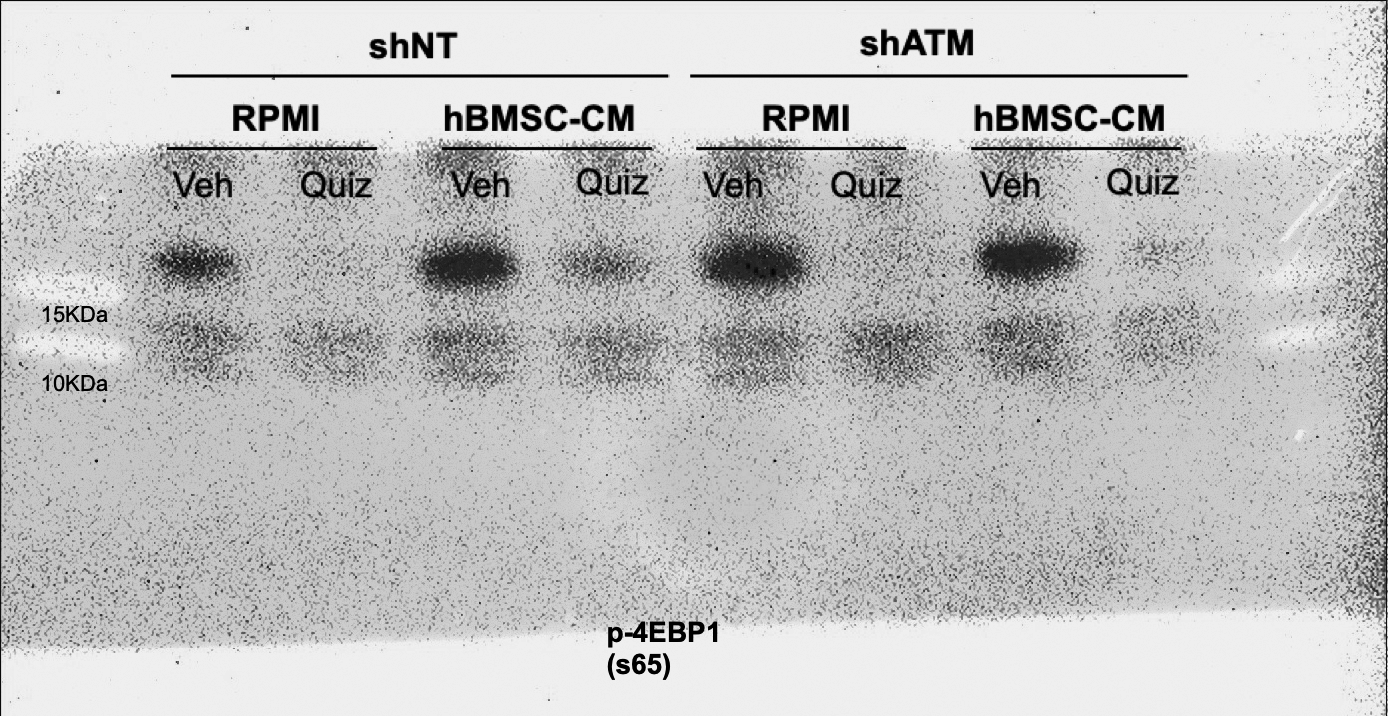

Supplement: Figure 7—source data 4. [file elife-79940-fig7-data4.zip › 7C-p4EBP1(s65) labeled.tiff]

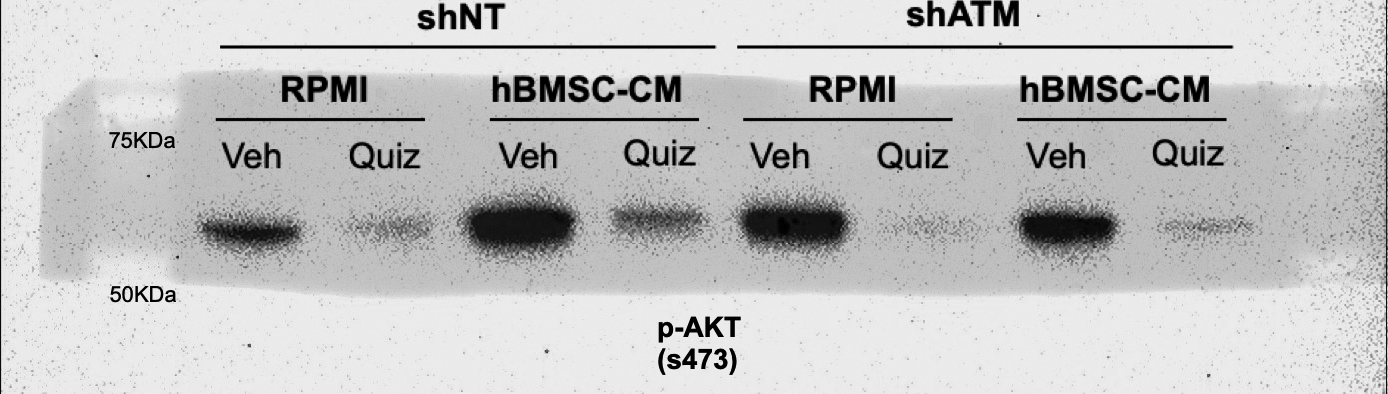

Supplement: Figure 7—source data 4. [file elife-79940-fig7-data4.zip › 7C-pAKT (s473) labdled.tiff]

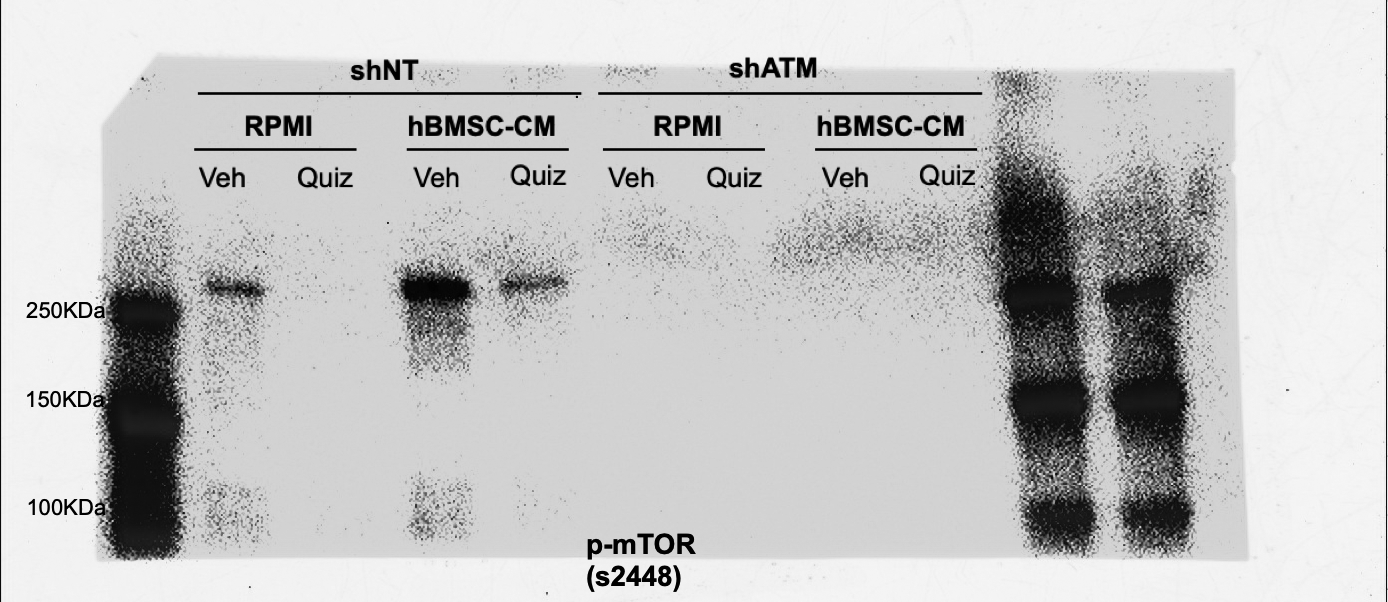

Supplement: Figure 7—source data 4. [file elife-79940-fig7-data4.zip › 7C-pMTOR (s2448) labeled.tiff]

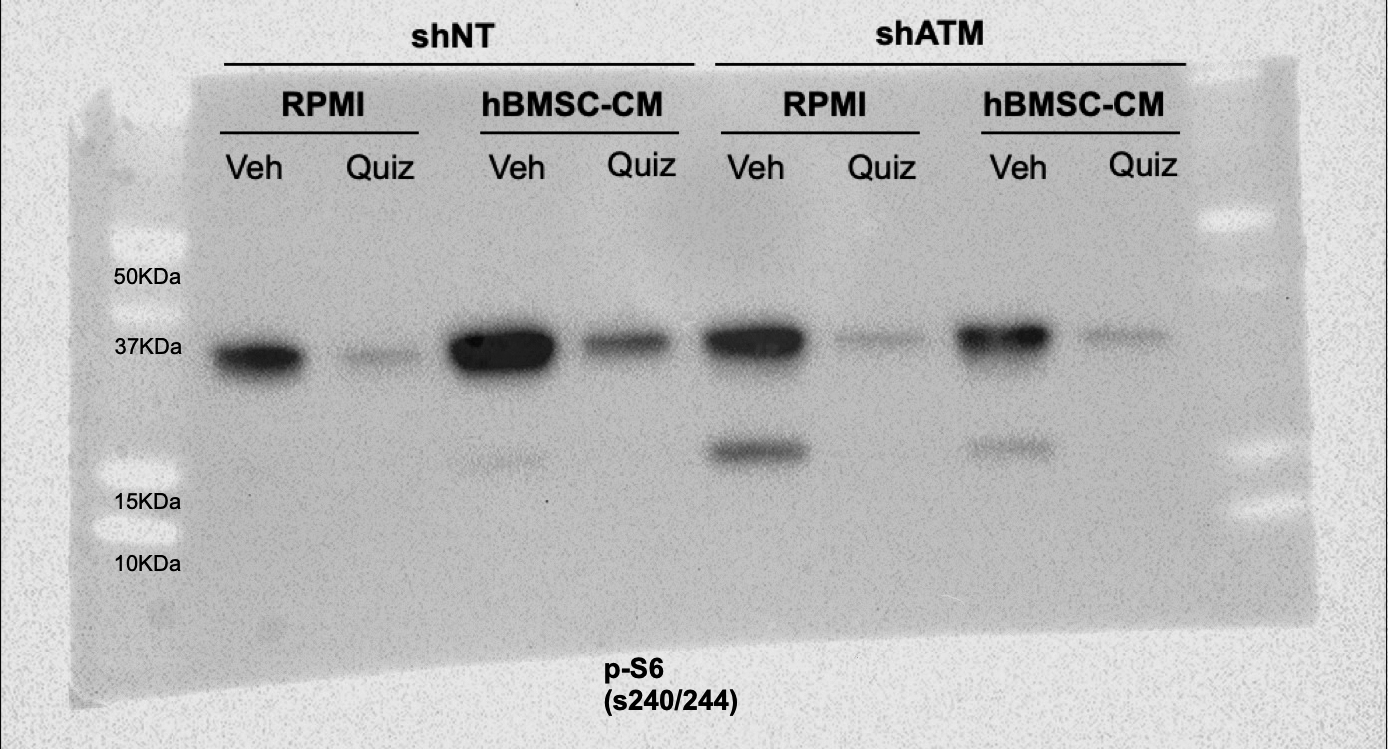

Supplement: Figure 7—source data 4. [file elife-79940-fig7-data4.zip › 7C-pS6 (s240:244) labeled.tiff]

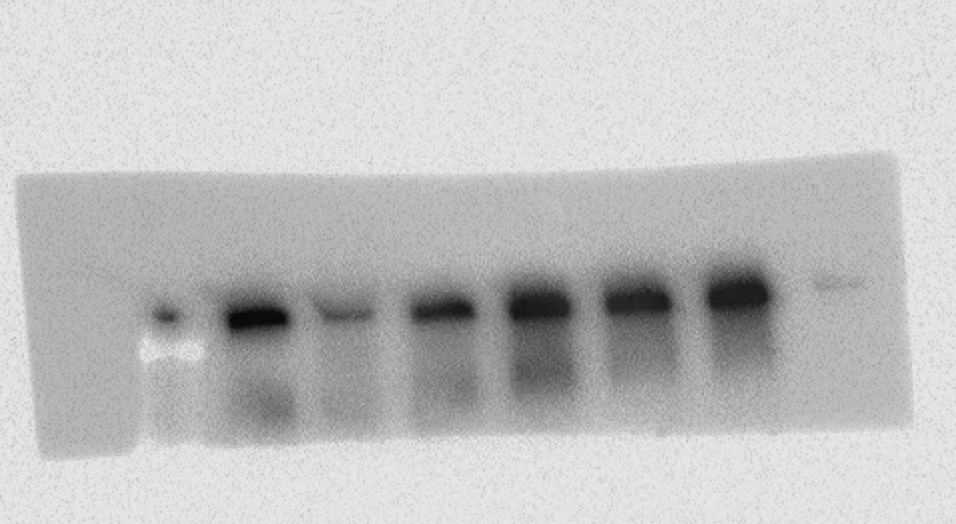

Supplement: Figure 7—figure supplement 1—source data 1. [file elife-79940-fig7-figsupp1-data1.zip › Fig 7- figure supplement 1-mTOR unlabeled.tif]

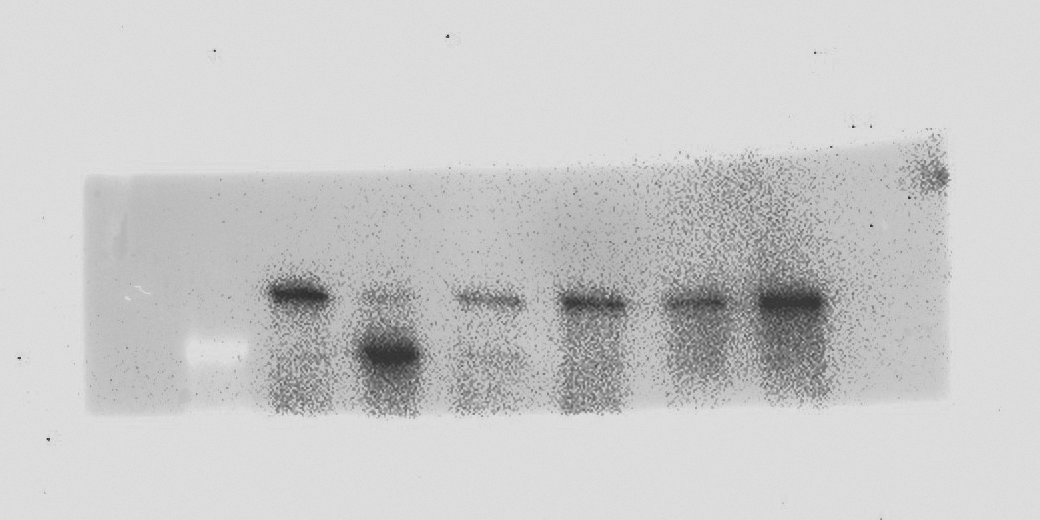

Supplement: Figure 7—figure supplement 1—source data 1. [file elife-79940-fig7-figsupp1-data1.zip › Fig 7- figure supplement 1- pATM(s1981) unlabeled.tif]

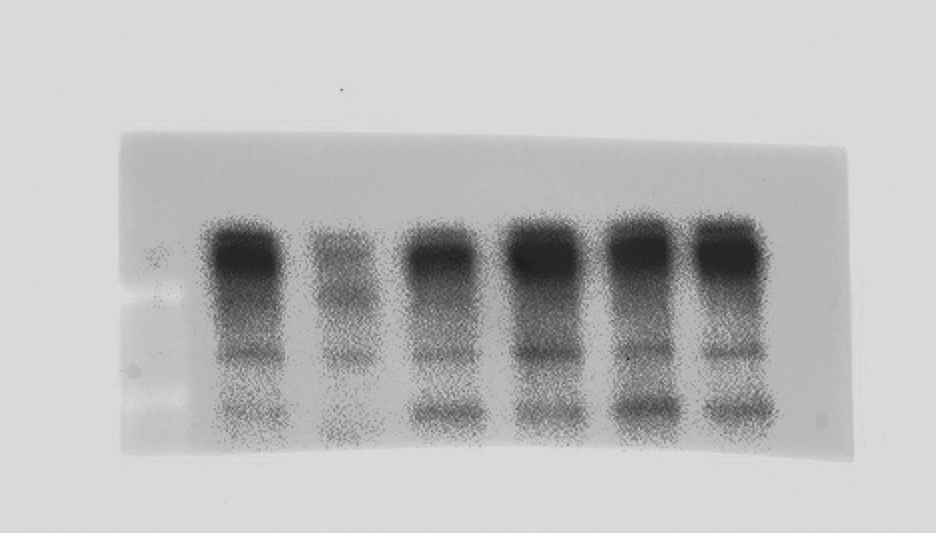

Supplement: Figure 7—figure supplement 1—source data 1. [file elife-79940-fig7-figsupp1-data1.zip › Fig 7- figure supplement 1-ATM unlabeled.tif]

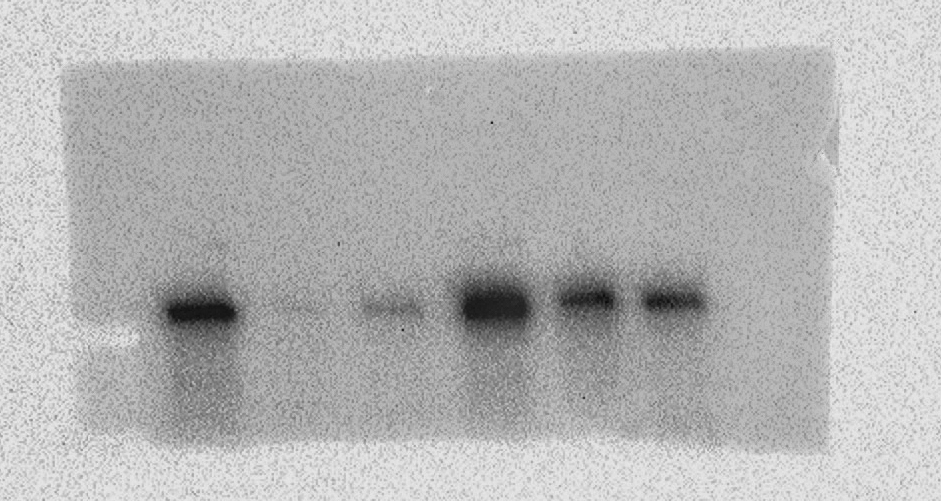

Supplement: Figure 7—figure supplement 1—source data 1. [file elife-79940-fig7-figsupp1-data1.zip › Fig 7-figure supplement 1-pMTOR (s2448) unlabeled.tif]

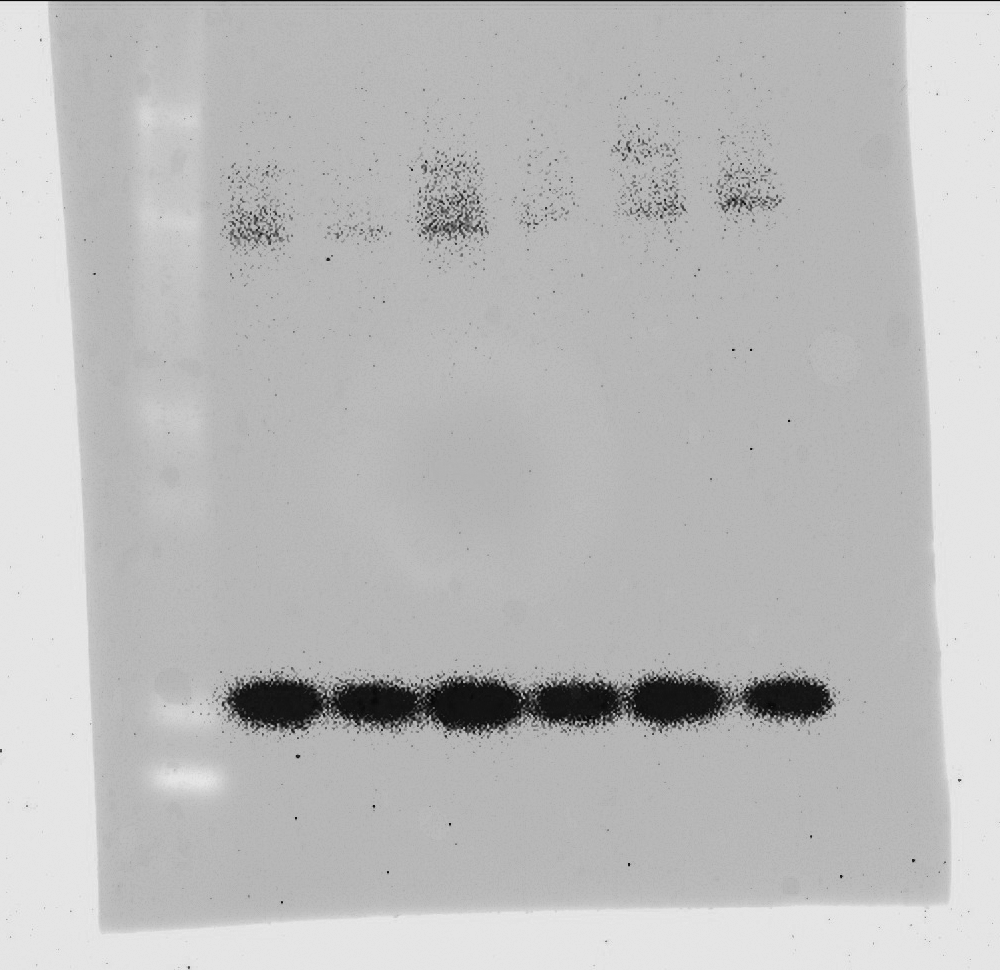

Supplement: Figure 7—figure supplement 1—source data 1. [file elife-79940-fig7-figsupp1-data1.zip › Fig 7-figure supplement 1-H3 unlabeled.tif]

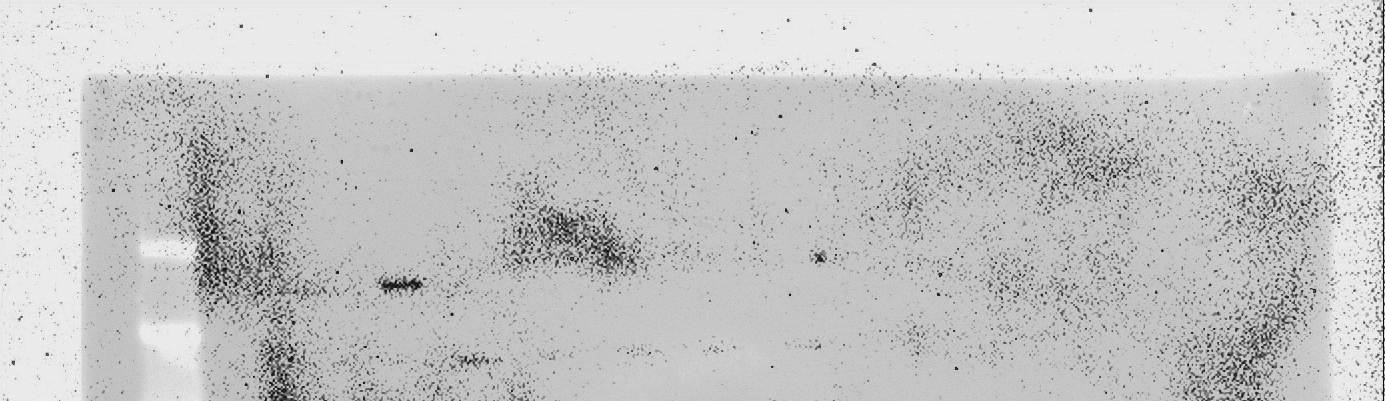

Supplement: Figure 7—figure supplement 1—source data 1. [file elife-79940-fig7-figsupp1-data1.zip › Fig7- figure supplement 1- cleaved caspase 3 unlabeled.tif]

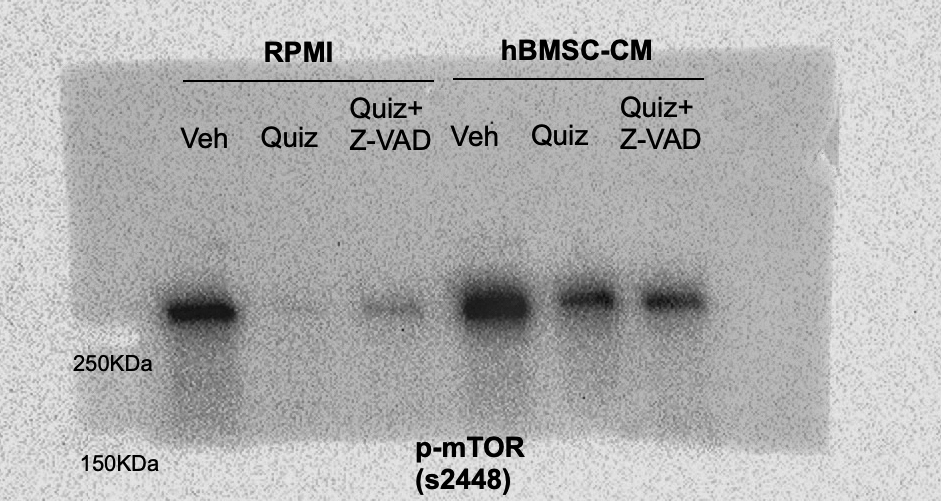

Supplement: Figure 7—figure supplement 1—source data 2. [file elife-79940-fig7-figsupp1-data2.zip › Fig 7-figure supplement 1-pMTOR (s2448) labeled .tiff]

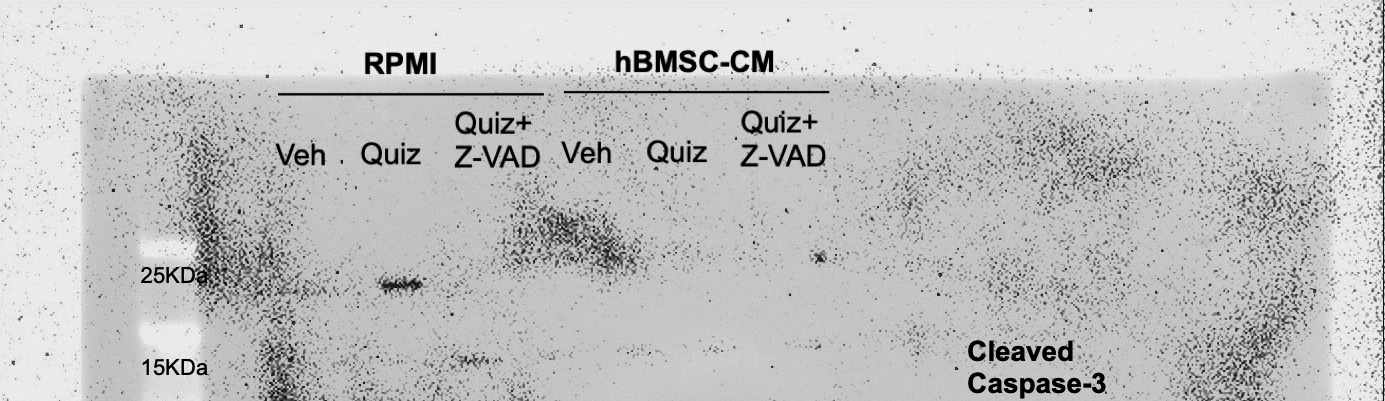

Supplement: Figure 7—figure supplement 1—source data 2. [file elife-79940-fig7-figsupp1-data2.zip › Fig7- figure supplement 1- cleaved caspase 3 labeled .tiff]

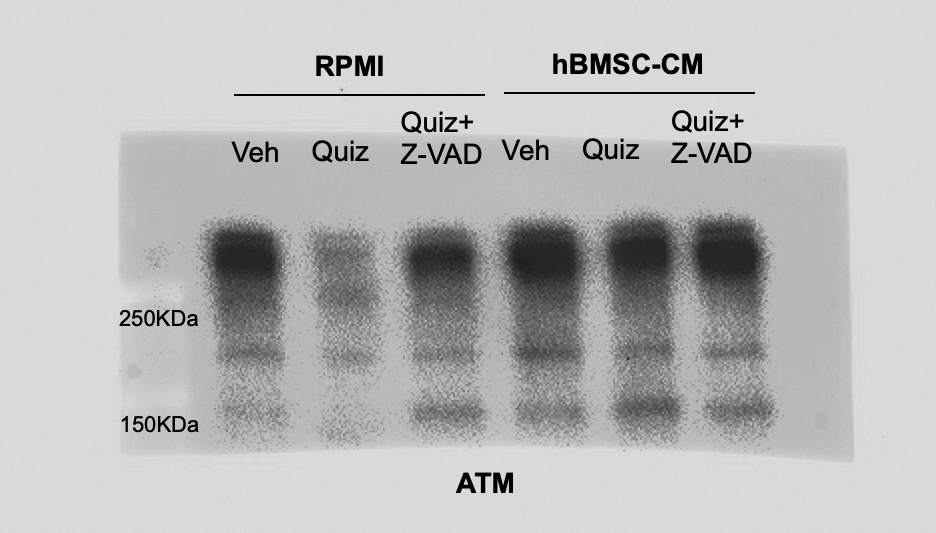

Supplement: Figure 7—figure supplement 1—source data 2. [file elife-79940-fig7-figsupp1-data2.zip › Fig 7- figure supplement 1-ATM labeled .tiff]

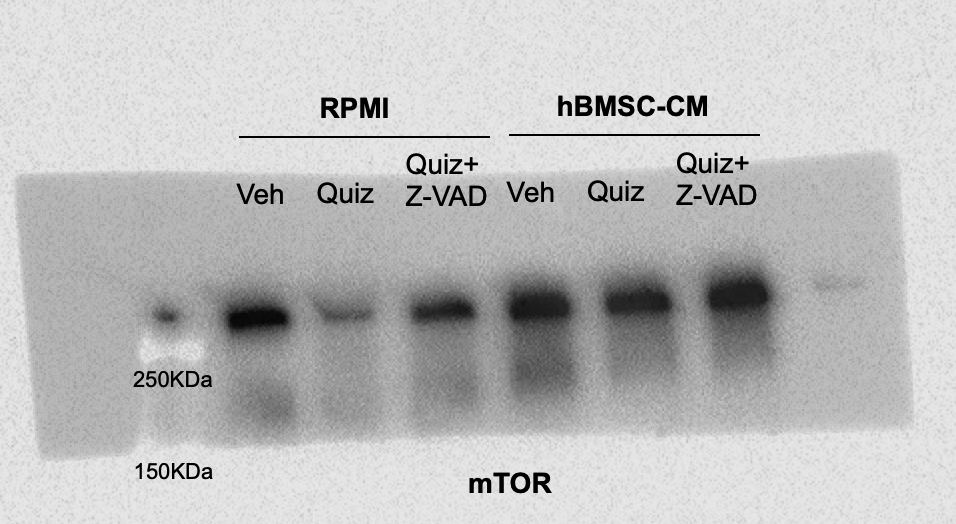

Supplement: Figure 7—figure supplement 1—source data 2. [file elife-79940-fig7-figsupp1-data2.zip › Fig 7- figure supplement 1-mTOR labeled.tiff]

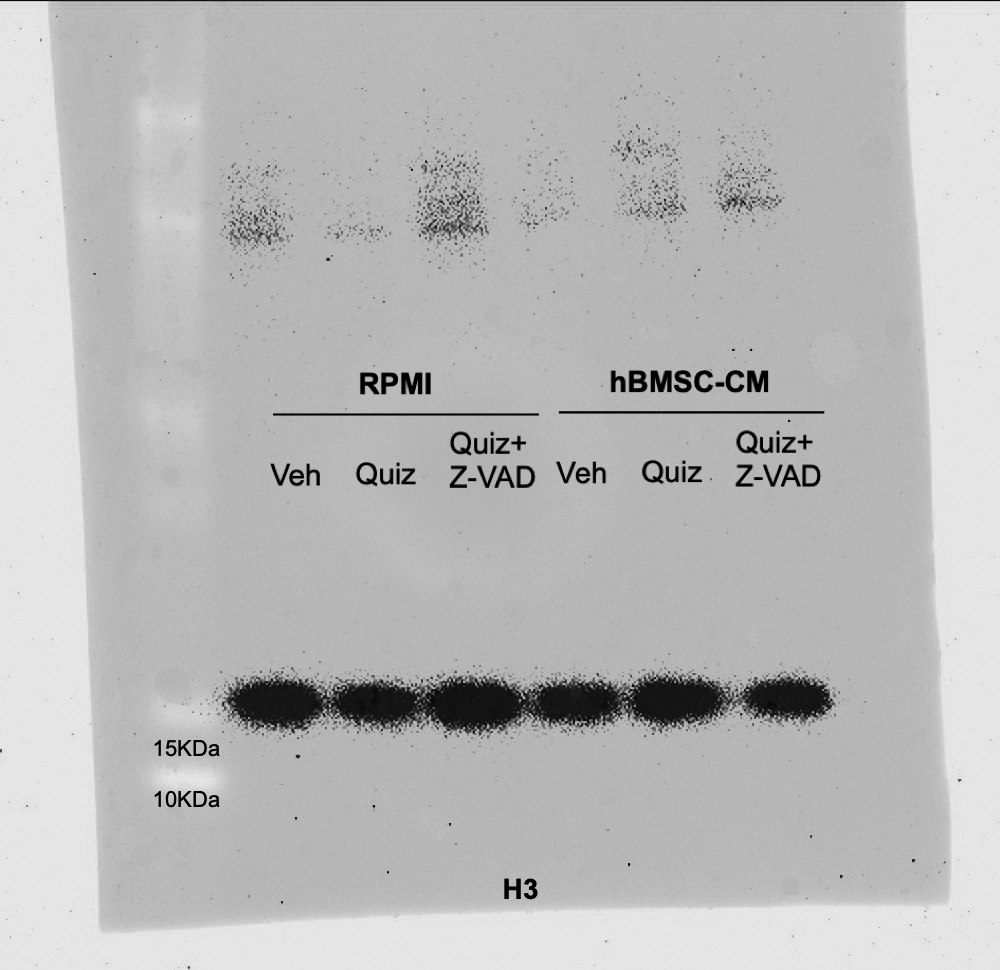

Supplement: Figure 7—figure supplement 1—source data 2. [file elife-79940-fig7-figsupp1-data2.zip › Fig 7-figure supplement 1-H3 labeled .tiff]

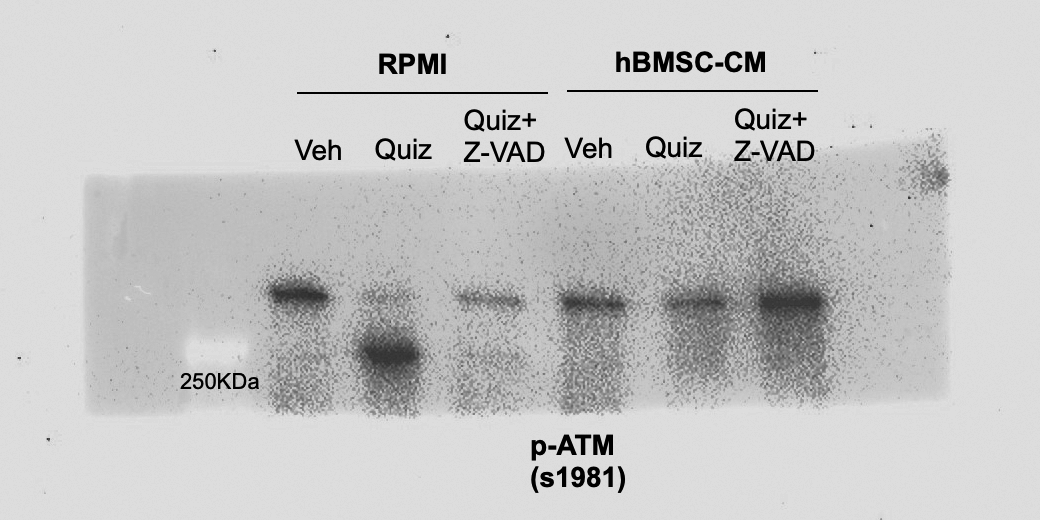

Supplement: Figure 7—figure supplement 1—source data 2. [file elife-79940-fig7-figsupp1-data2.zip › Fig 7- figure supplement 1- pATM(s1981) labeled .tiff]

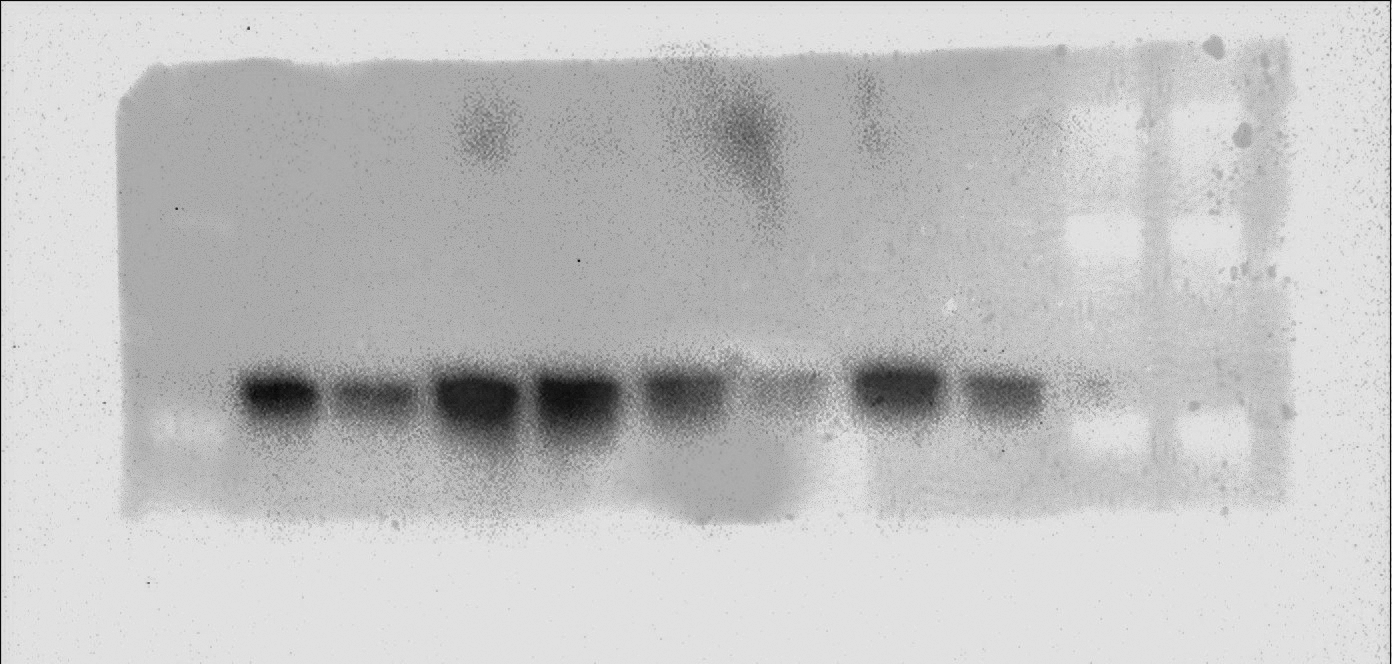

Supplement: Figure 7—figure supplement 5—source data 1. [file elife-79940-fig7-figsupp5-data1.zip › Fig 7- figure supplement 5- AKT unlabeled.tif]

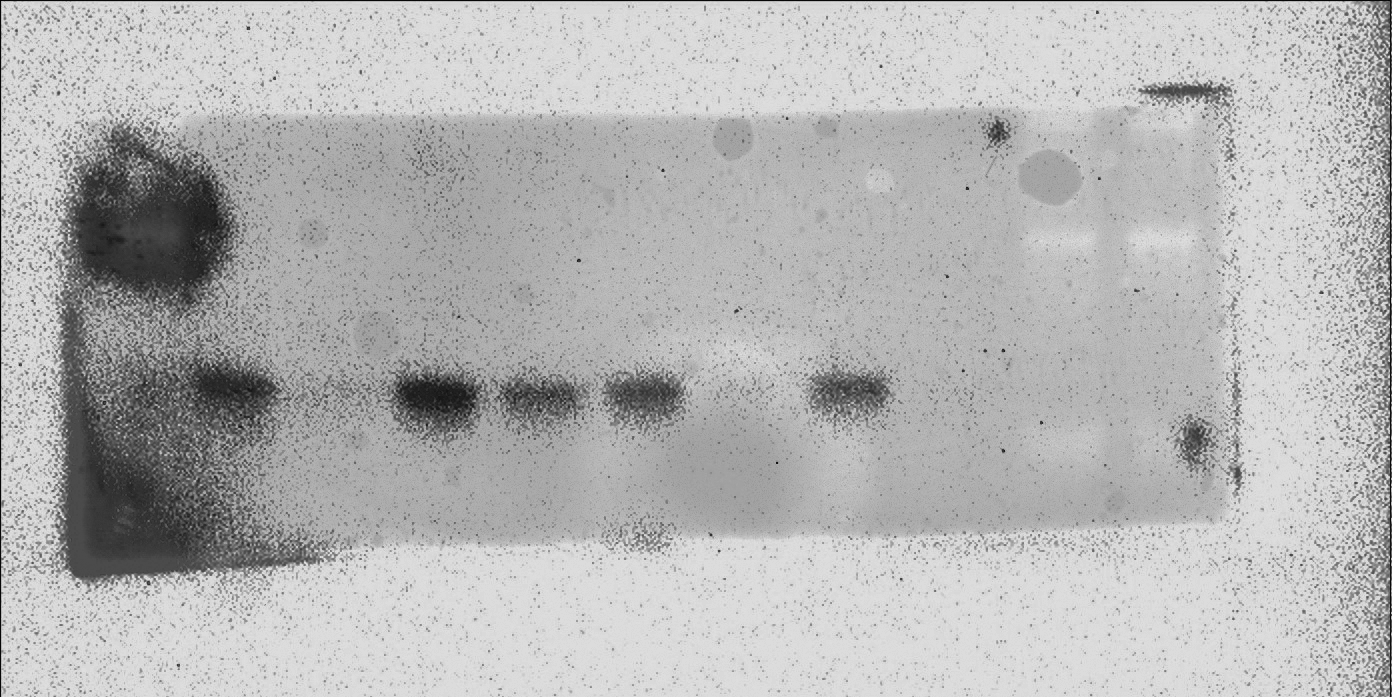

Supplement: Figure 7—figure supplement 5—source data 1. [file elife-79940-fig7-figsupp5-data1.zip › Fig 7- figure supplement 5-pAKT (s473) unlabeled.tif]

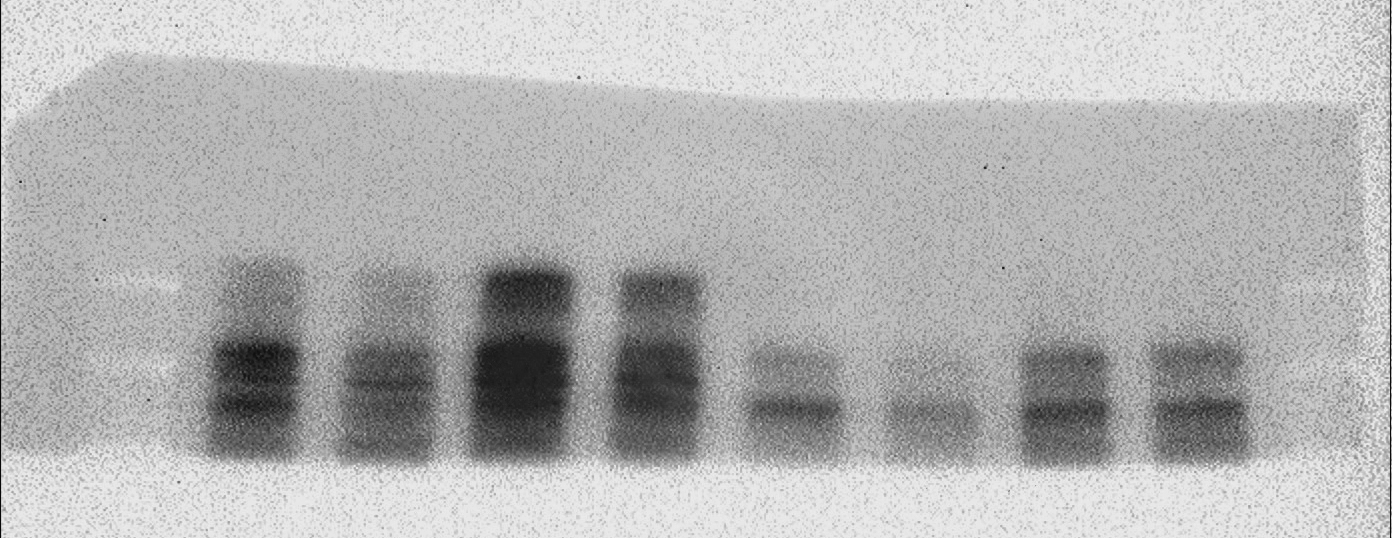

Supplement: Figure 7—figure supplement 5—source data 1. [file elife-79940-fig7-figsupp5-data1.zip › Fig 7- figure supplement 5-ATM unlabeled.tif]

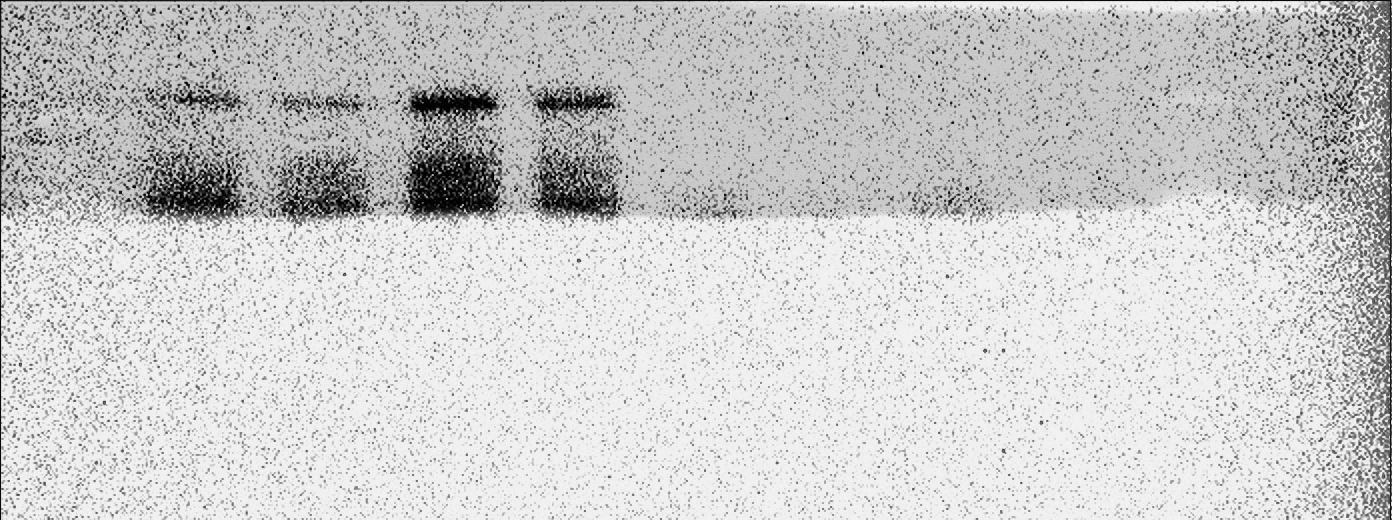

Supplement: Figure 7—figure supplement 5—source data 1. [file elife-79940-fig7-figsupp5-data1.zip › Fig 7- figure supplement 5- mTOR unlabeled.tif]

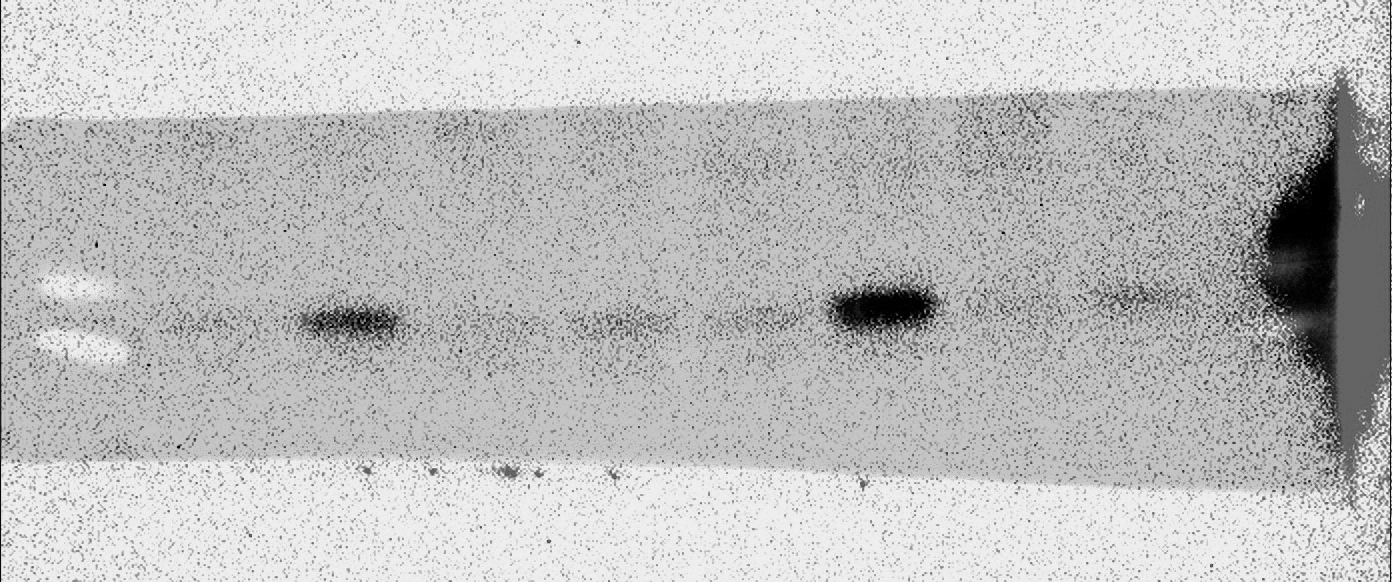

Supplement: Figure 7—figure supplement 5—source data 1. [file elife-79940-fig7-figsupp5-data1.zip › Fig 7- figure supplement 5- pH2AX (s139) unlabeled.tif]

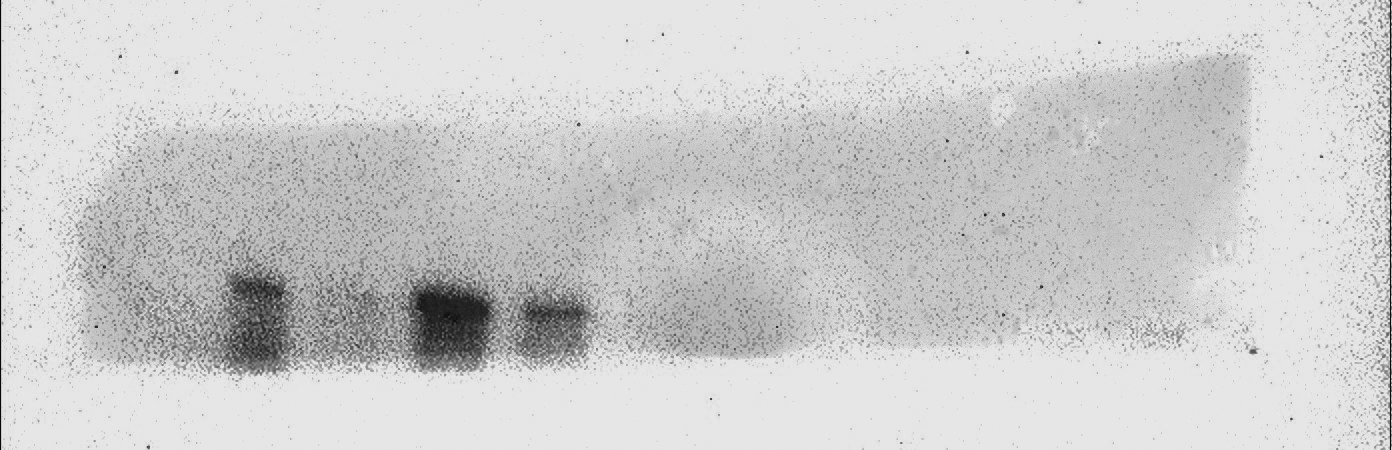

Supplement: Figure 7—figure supplement 5—source data 1. [file elife-79940-fig7-figsupp5-data1.zip › Fig 7- figure supplement 5- pMTOR (s2448) unlabeled.tif]

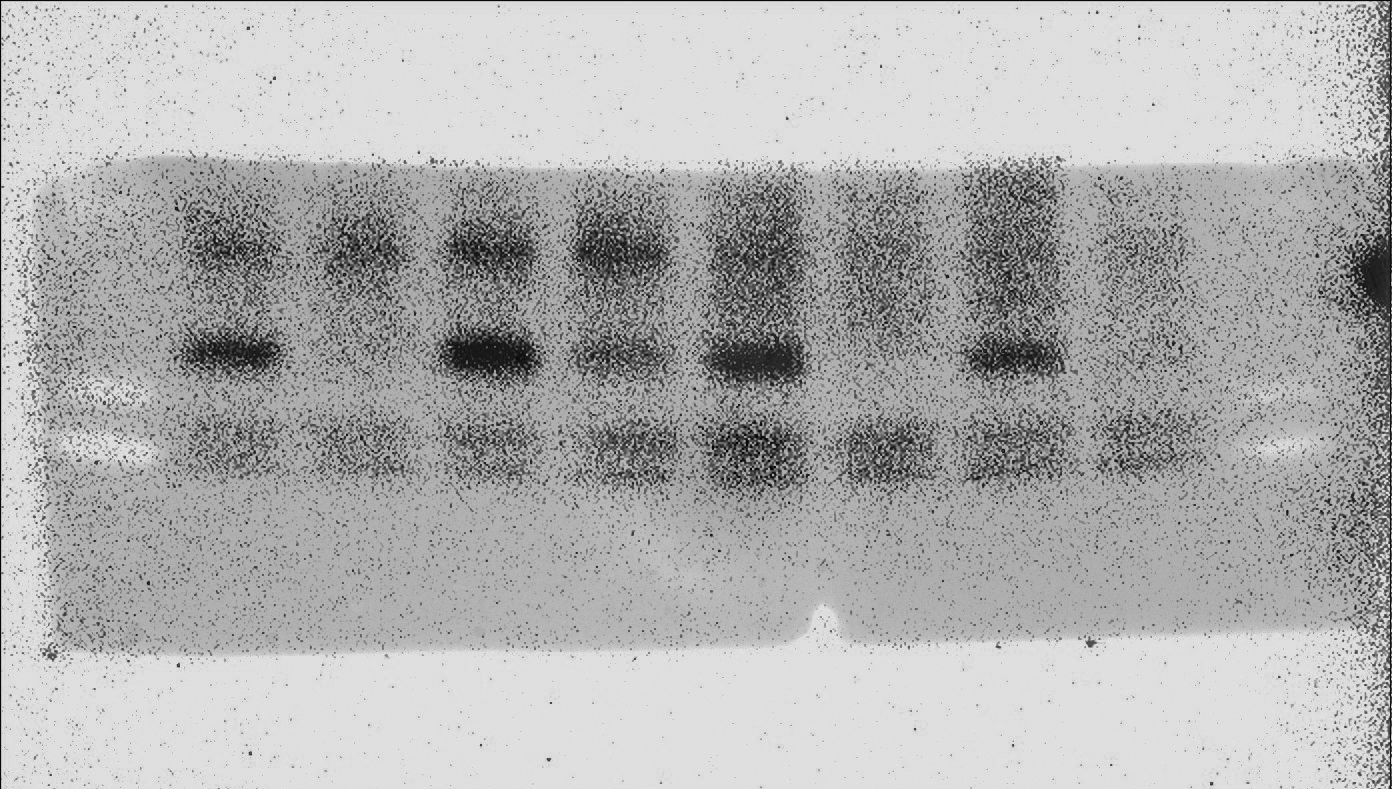

Supplement: Figure 7—figure supplement 5—source data 1. [file elife-79940-fig7-figsupp5-data1.zip › Fig 7- figure supplement 5- p4EBP1 (T37:46) unlabeled.tif]

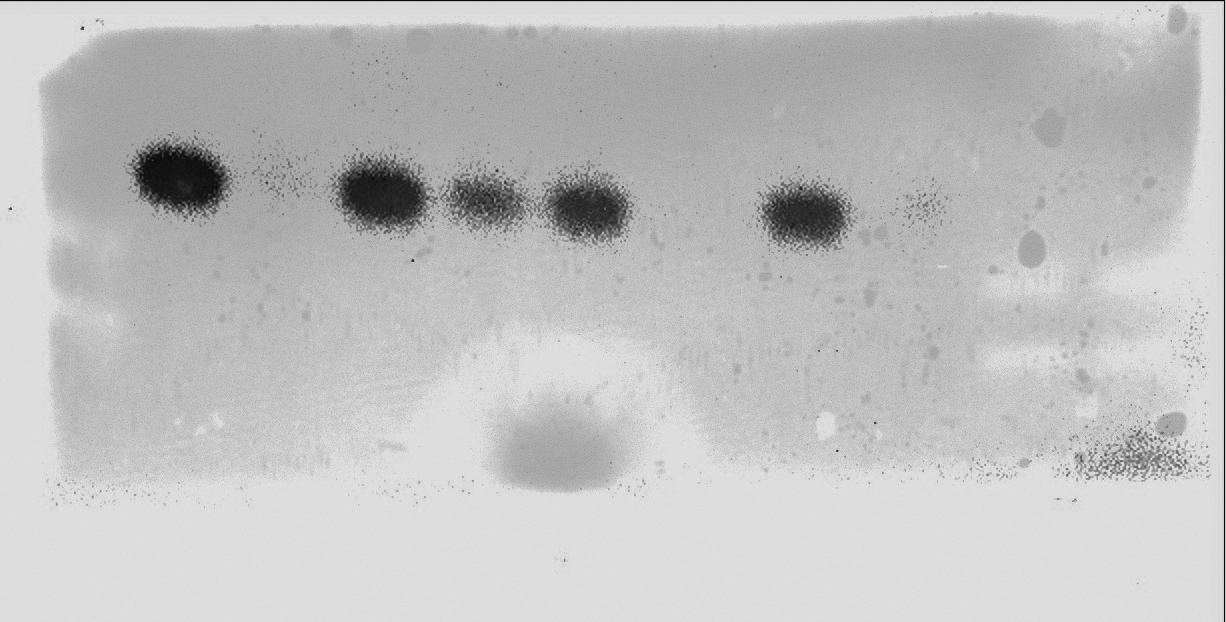

Supplement: Figure 7—figure supplement 5—source data 1. [file elife-79940-fig7-figsupp5-data1.zip › Fig 7- figure supplement 5- pS6 (s240:244) unlabeled.tif]

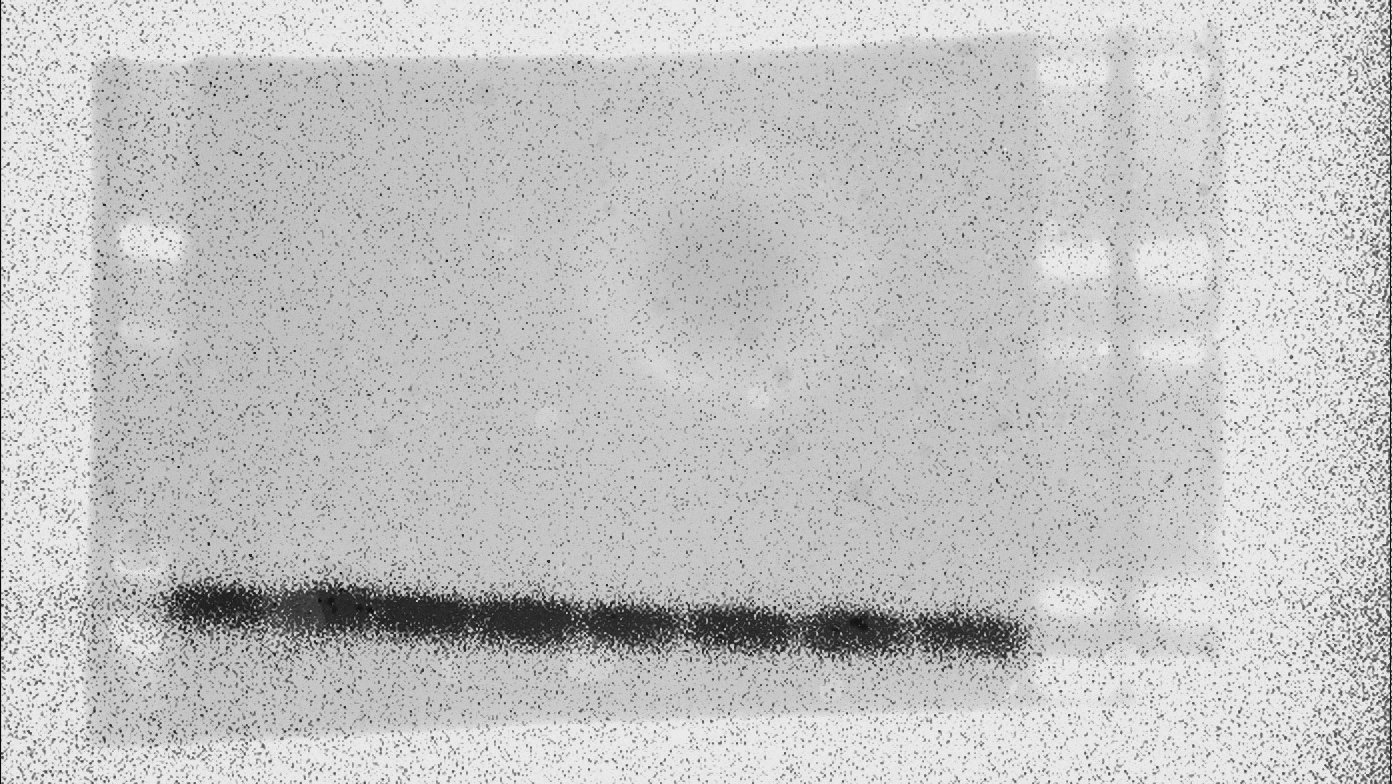

Supplement: Figure 7—figure supplement 5—source data 1. [file elife-79940-fig7-figsupp5-data1.zip › Fig 7- figure supplement 5- H3 unlabeled.tif]

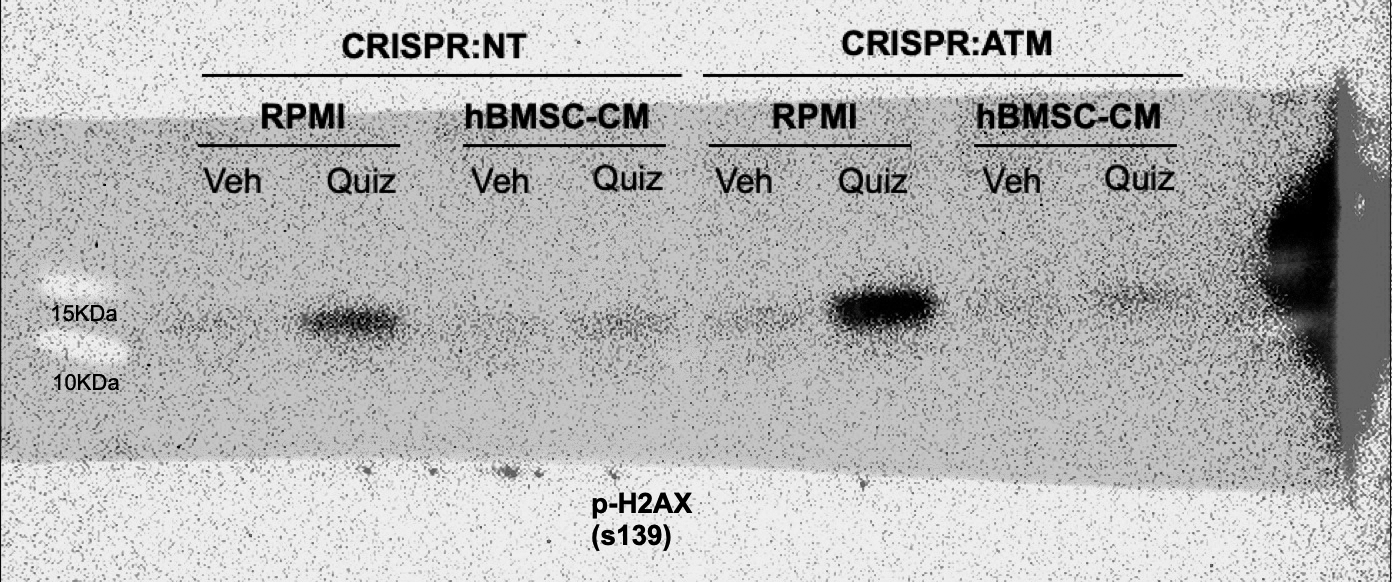

Supplement: Figure 7—figure supplement 5—source data 2. [file elife-79940-fig7-figsupp5-data2.zip › Fig 7- figure supplement 5- pH2AX (s139) labeled.tiff]

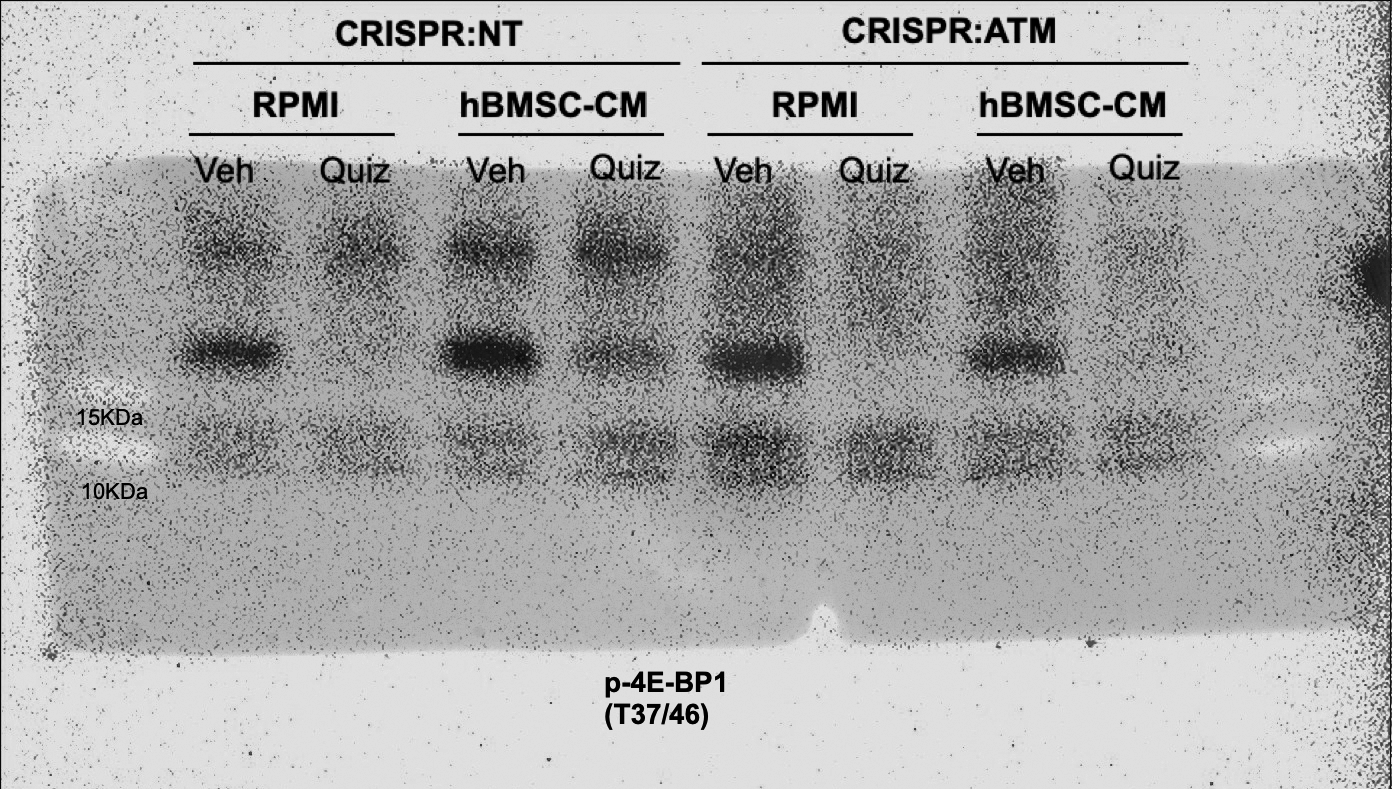

Supplement: Figure 7—figure supplement 5—source data 2. [file elife-79940-fig7-figsupp5-data2.zip › Fig 7- figure supplement 5- p4EBP1 (T37:46) labeled.tiff]

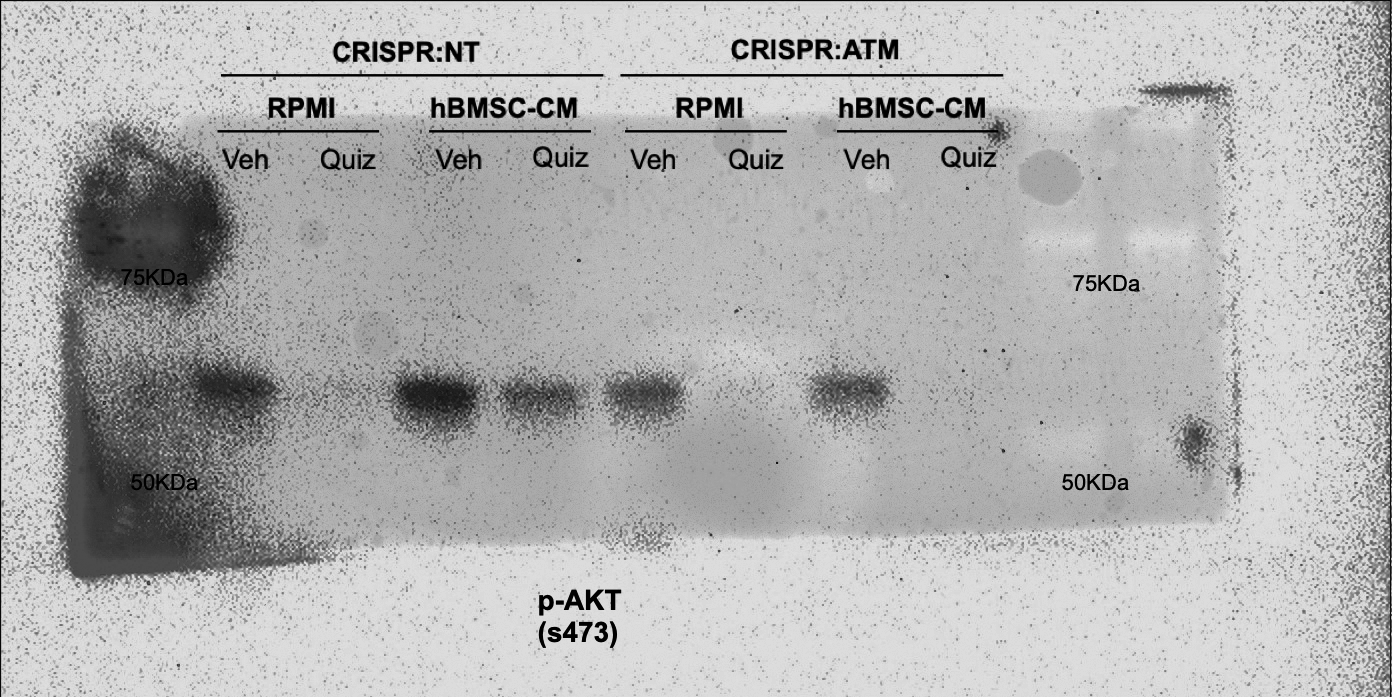

Supplement: Figure 7—figure supplement 5—source data 2. [file elife-79940-fig7-figsupp5-data2.zip › Fig 7- figure supplement 5-pAKT (s473) labeled.tiff]

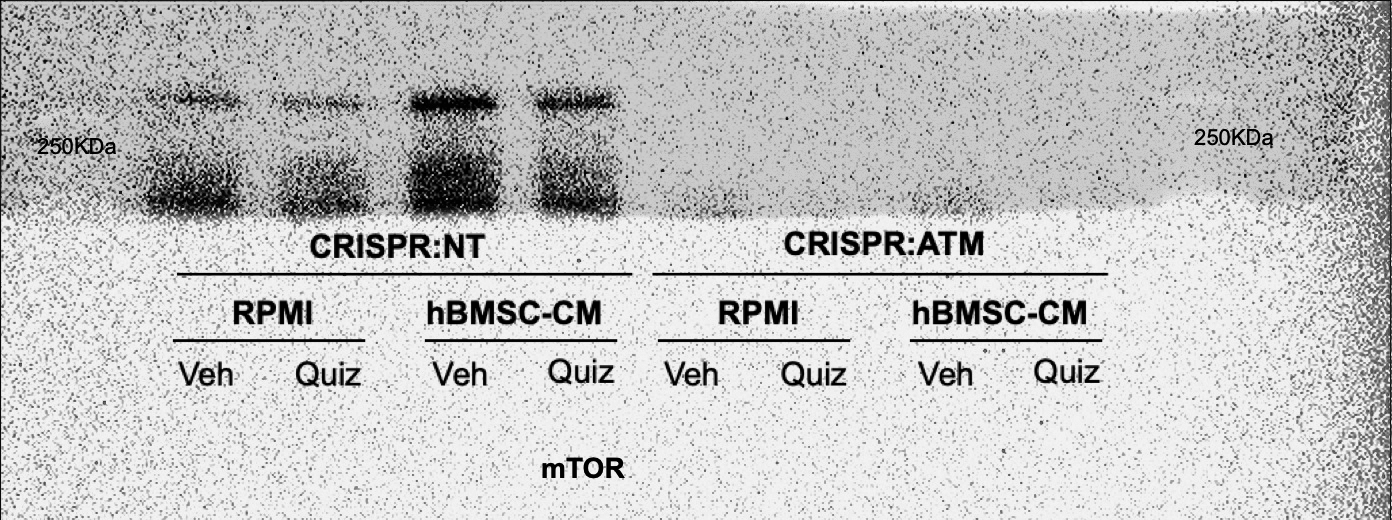

Supplement: Figure 7—figure supplement 5—source data 2. [file elife-79940-fig7-figsupp5-data2.zip › Fig 7- figure supplement 5- mTOR labeled.tiff]

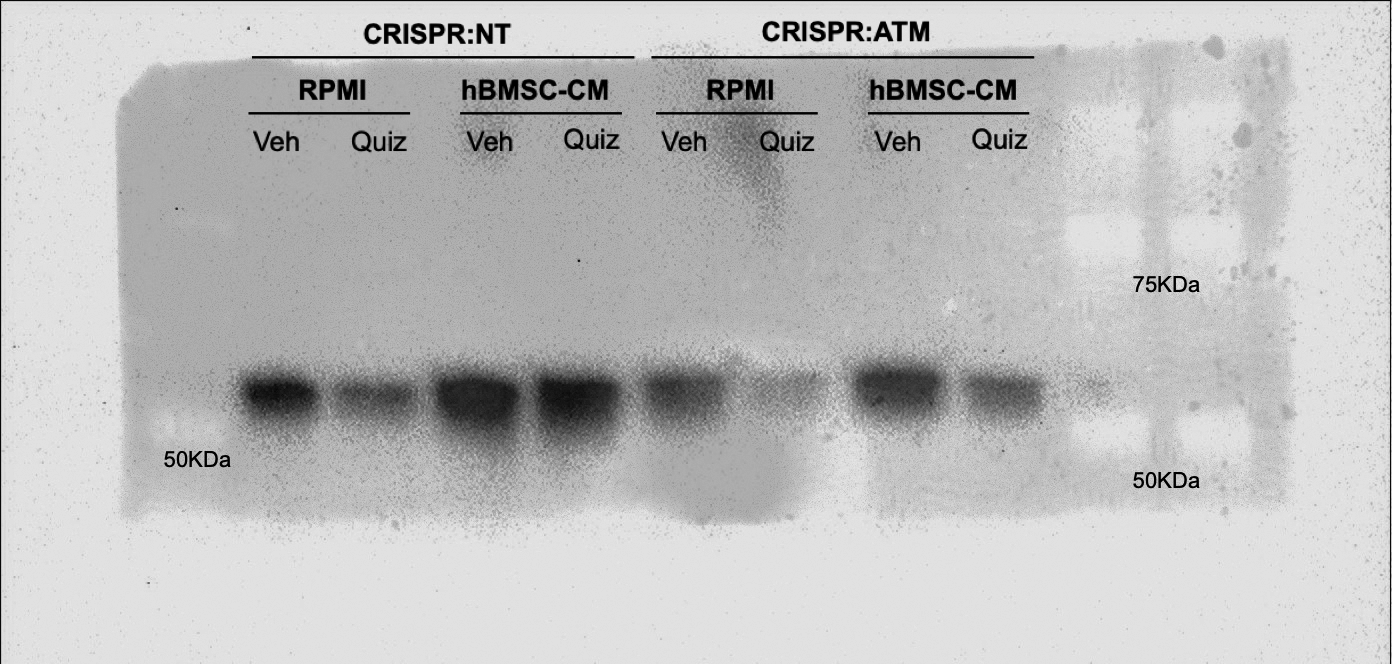

Supplement: Figure 7—figure supplement 5—source data 2. [file elife-79940-fig7-figsupp5-data2.zip › Fig 7- figure supplement 5- AKT labeled.tiff]

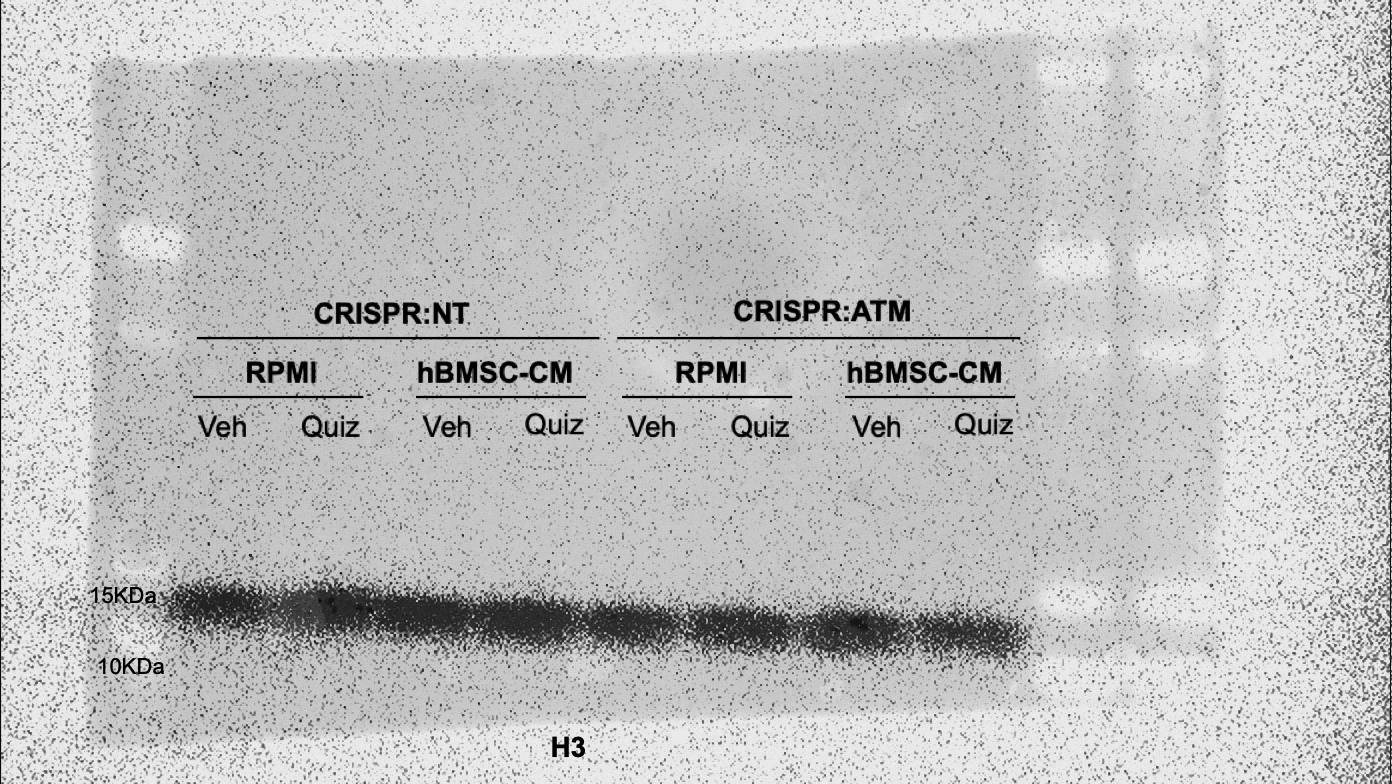

Supplement: Figure 7—figure supplement 5—source data 2. [file elife-79940-fig7-figsupp5-data2.zip › Fig 7- figure supplement 5- H3 labeled.tiff]

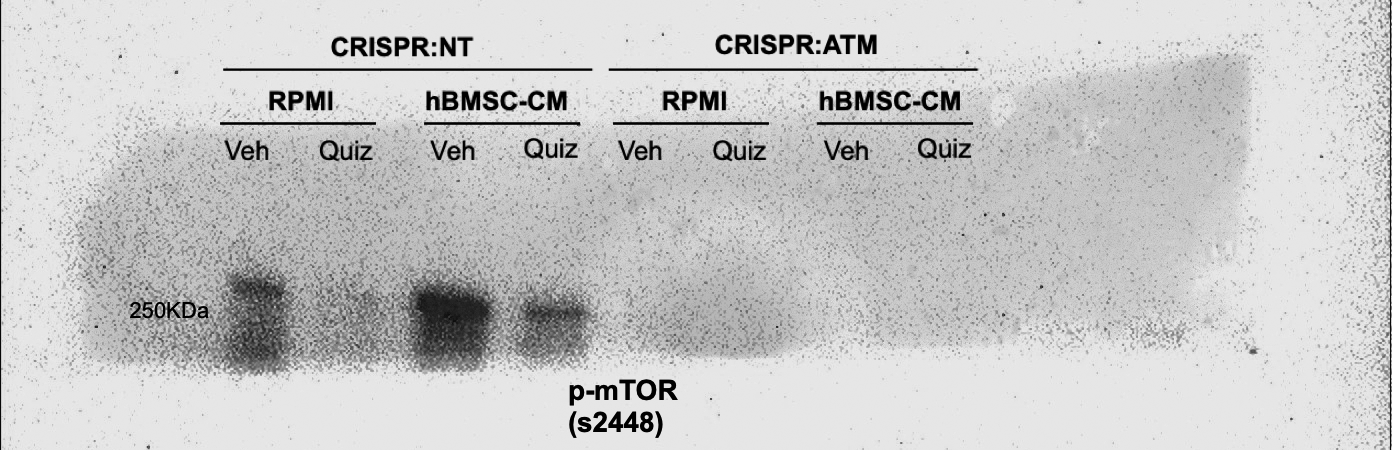

Supplement: Figure 7—figure supplement 5—source data 2. [file elife-79940-fig7-figsupp5-data2.zip › Fig 7- figure supplement 5- pMTOR (s2448) labeled.tiff]
