# Supplementary material for: Therapeutic resistance in acute myeloid leukemia cells is mediated by a novel ATM/mTOR pathway regulating oxidative phosphorylation
Source: eLife. 2022 Oct 19;11:e79940. doi: 10.7554/eLife.79940 (PMC9645811; doi:10.7554/eLife.79940)
Supplement: Figure 2—source data 3. — Top 10 enrichment pathways (by p-adj) from gene set enrichment analyses (GSEA) of Hallmark gene sets applied on RNA-seq data from human acute myeloid leukemia (AML) cells. Samples from the spleen of vehicle-treated mice were compared with the samples from the BM of the same paired mouse. Pathways with significant alteration (p-adj<0.1) are represented with colors (red: negative normalized enrichment score [NES]; blue: positive NES). Asterisks represent matched pathways with same enrichment patterns observed from GSEA of Hallmark gene sets comparing the spleen from quizartinib-treated mice with BM of the same paired mouse (Figure 2G). [file elife-79940-fig2-data3.pdf]

Figure 2- figure supplement 4

**Top10 pathways- HALLMARK**<br>**Vehicle-treated mice**<br>**Spleen vs. Bone marrow**	**p-adj**	**NES**
*HALLMARK_OXIDATIVE_PHOSPHORYLATION	0.00482	-1.70337
HALLMARK_CHOLESTEROL_HOMEOSTASIS	0.03956	-1.67506
*HALLMARK_MITOTIC_SPINDLE	0.00482	2.44152
*HALLMARK_UV_RESPONSE_DN	0.00482	1.76716
*HALLMARK_ G2M_CHECKPOINT	0.00482	1.68288
*HALLMARK_NOTCH_SIGNALING	0.03956	1.77514
HALLMARK_ E2F_TARGETS	0.03956	1.41857
HALLMARK_ PROTEIN_SECRETION	0.08533	1.45851
*HALLMARK_ APICAL_JUNCTION	0.09197	1.40337
HALLMARK_TNFA_SIGNALING_VIA_NFKB	0.09354	1.36889
