# Supplementary figures and images for: Therapeutic resistance in acute myeloid leukemia cells is mediated by a novel ATM/mTOR pathway regulating oxidative phosphorylation (part 2 of 2)
Source: eLife. 2022 Oct 19;11:e79940. doi: 10.7554/eLife.79940 (PMC9645811; doi:10.7554/eLife.79940)

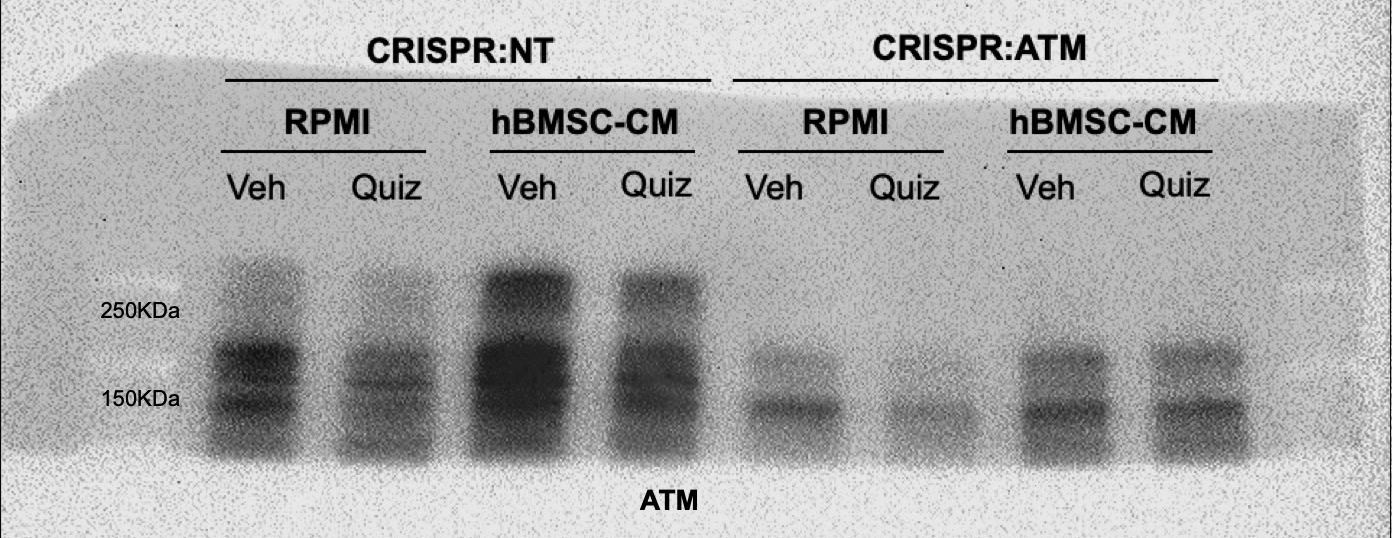

Supplement: Figure 7—figure supplement 5—source data 2. [file elife-79940-fig7-figsupp5-data2.zip › Fig 7- figure supplement 5-ATM labeled.tiff]

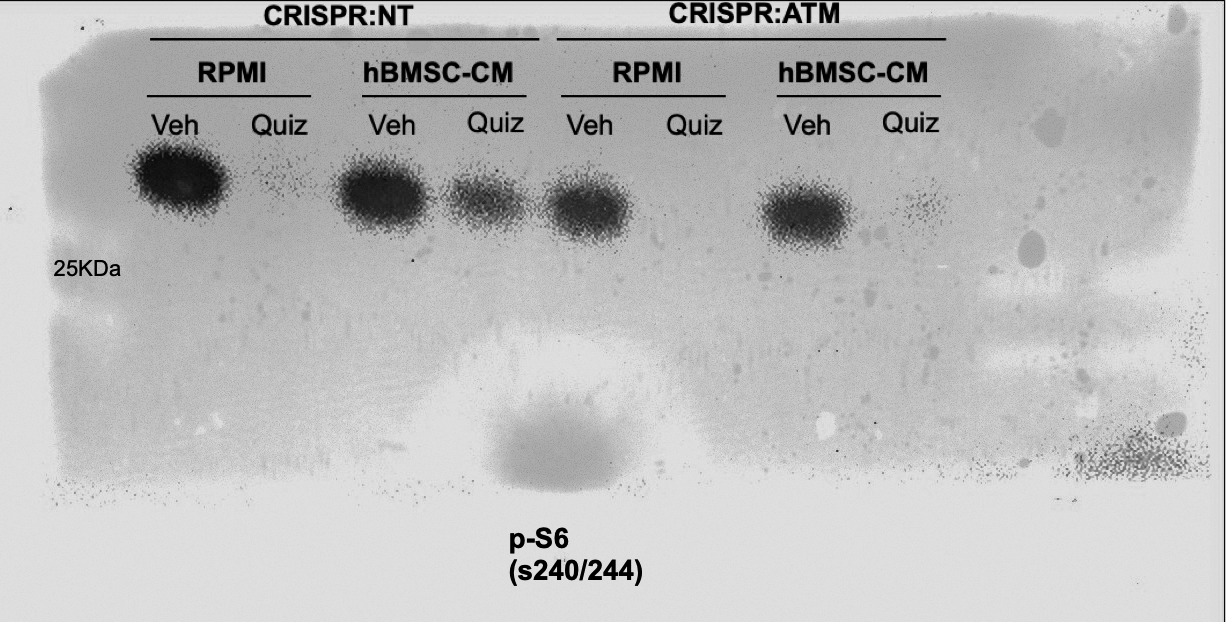

Supplement: Figure 7—figure supplement 5—source data 2. [file elife-79940-fig7-figsupp5-data2.zip › Fig 7- figure supplement 5- pS6 (s240:244) labeled .tiff]

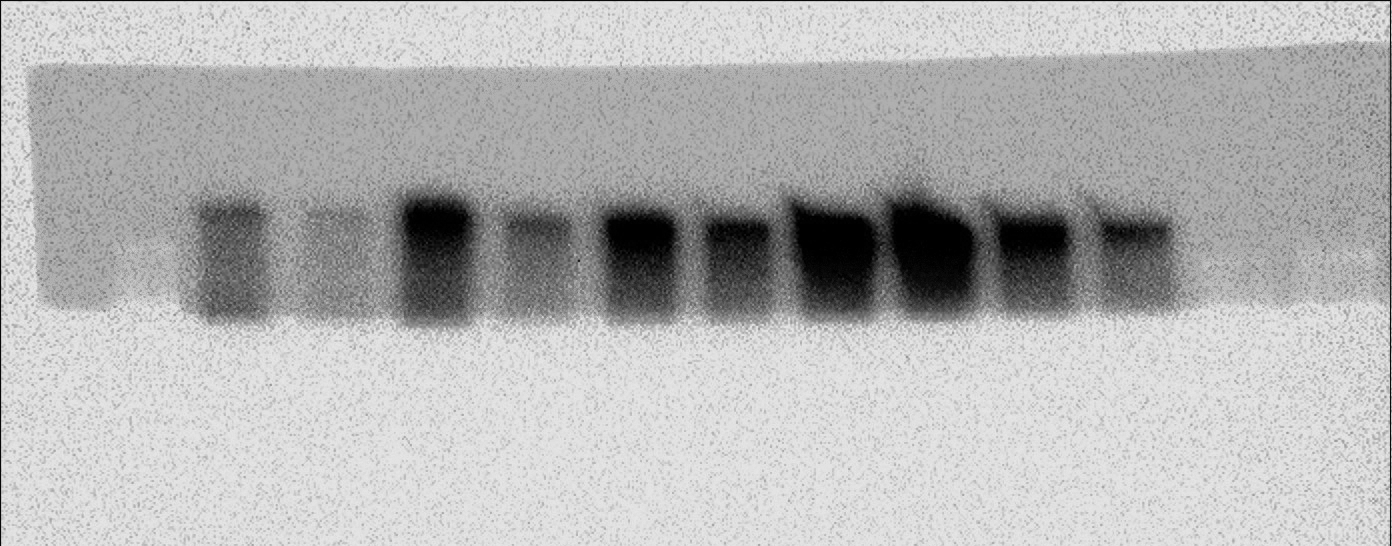

Supplement: Figure 7—figure supplement 6—source data 1. [file elife-79940-fig7-figsupp6-data1.zip › Fig 7- figure supplement 6- ATM unlabeled.tif]

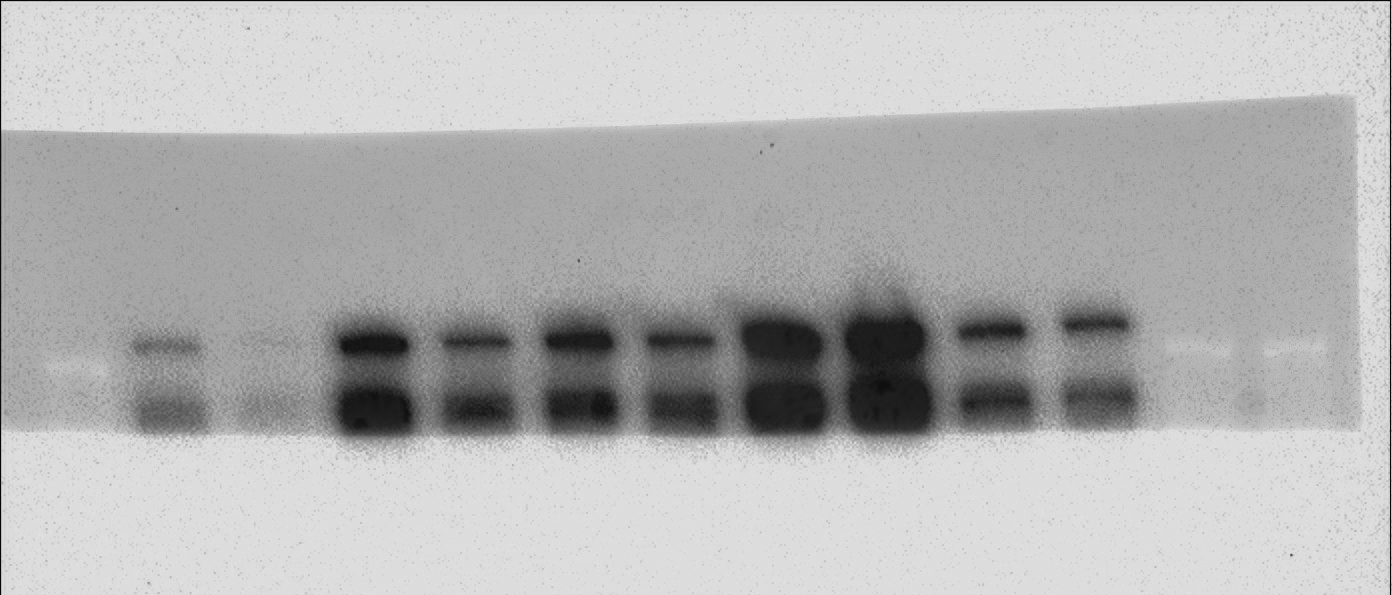

Supplement: Figure 7—figure supplement 6—source data 1. [file elife-79940-fig7-figsupp6-data1.zip › Fig 7- figure supplement 6- mTOR unlabeled.tif]

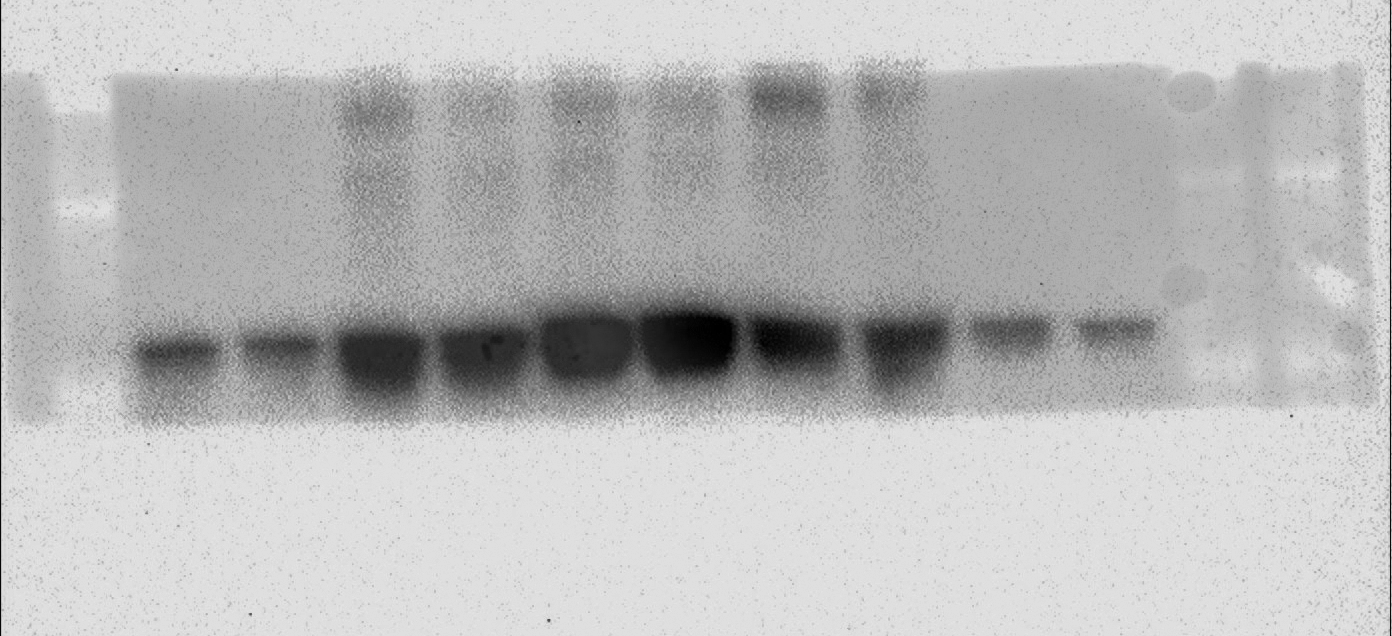

Supplement: Figure 7—figure supplement 6—source data 1. [file elife-79940-fig7-figsupp6-data1.zip › Fig 7- figure supplement 6- AKT unlabeled.tif]

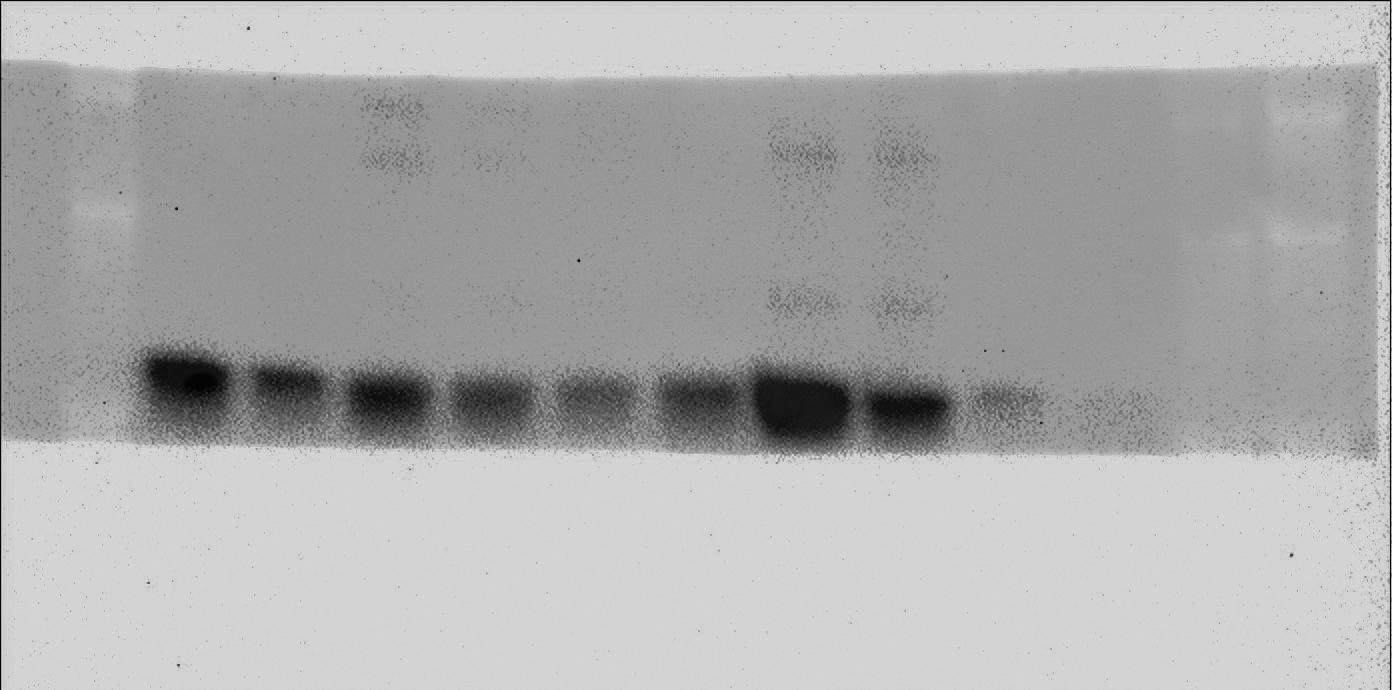

Supplement: Figure 7—figure supplement 6—source data 1. [file elife-79940-fig7-figsupp6-data1.zip › Fig 7- figure supplement 6- pAKT (s473) unlabeled.tif]

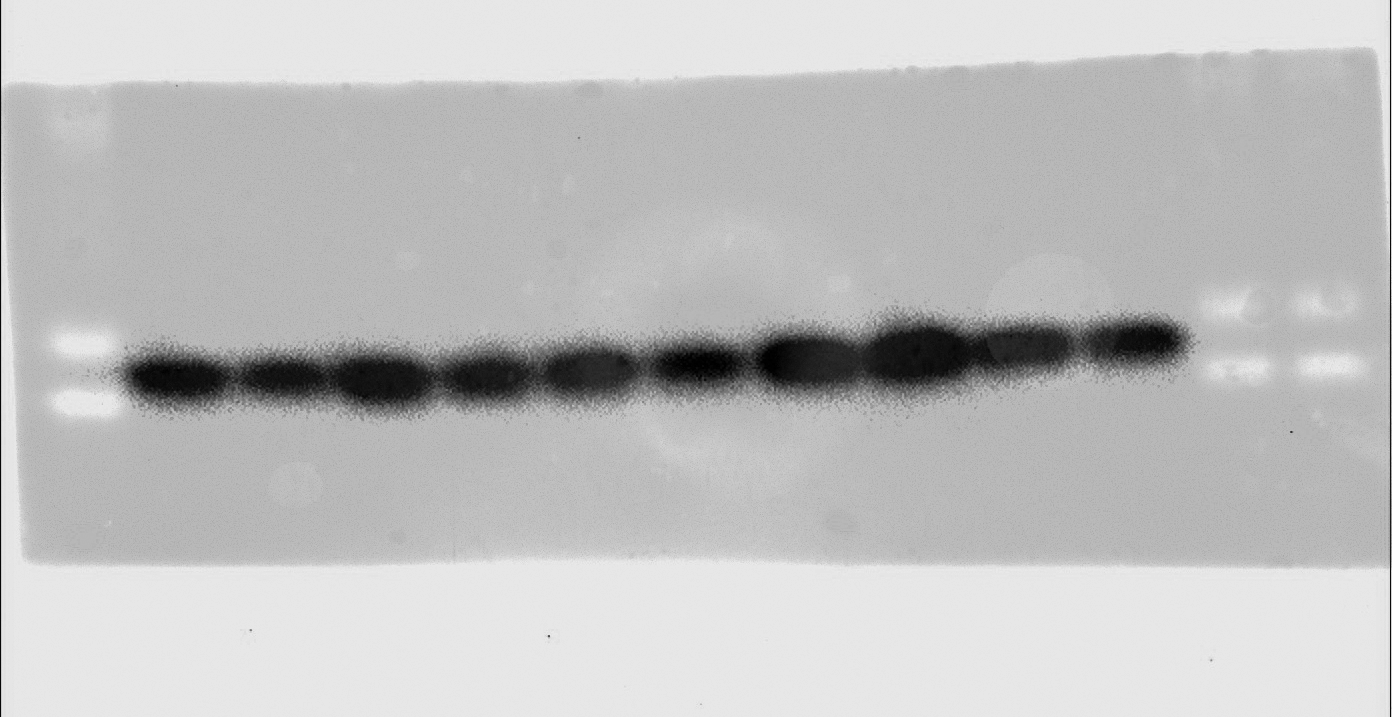

Supplement: Figure 7—figure supplement 6—source data 1. [file elife-79940-fig7-figsupp6-data1.zip › Fig 7- figure supplement 6- H3 unlabeld.tif]

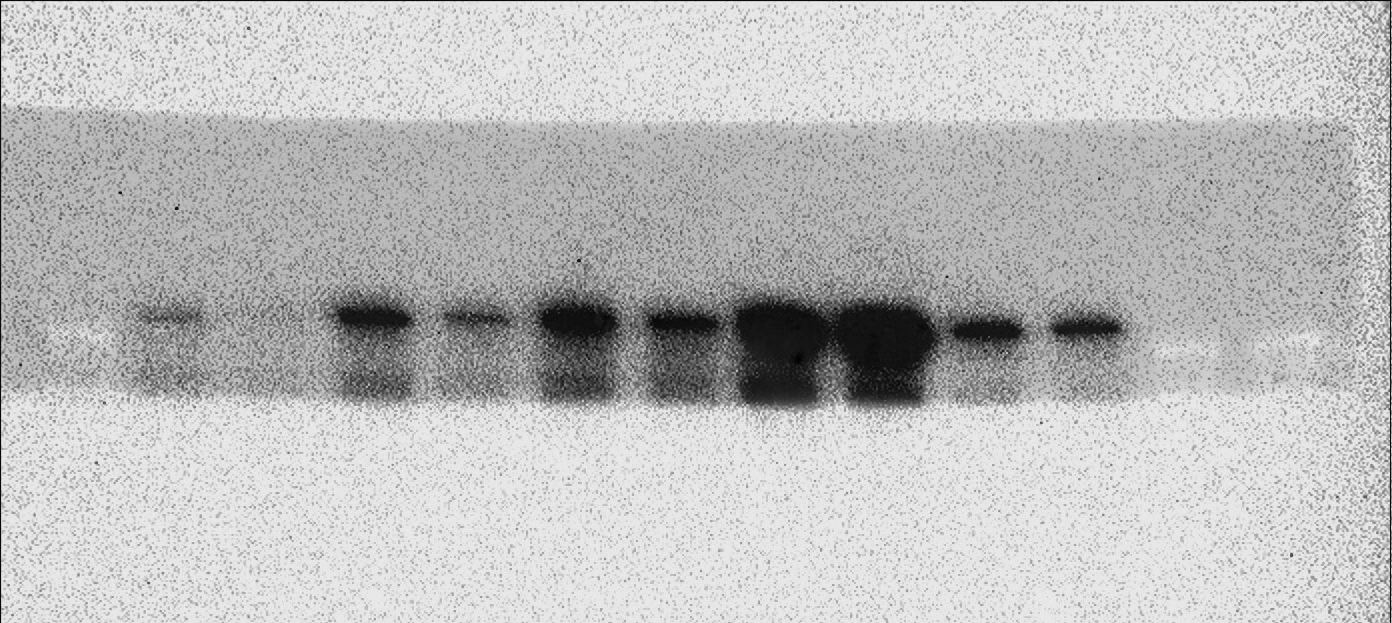

Supplement: Figure 7—figure supplement 6—source data 1. [file elife-79940-fig7-figsupp6-data1.zip › Fig 7- figure supplement 6- pMTOR (s2448) unlabeled.tif]

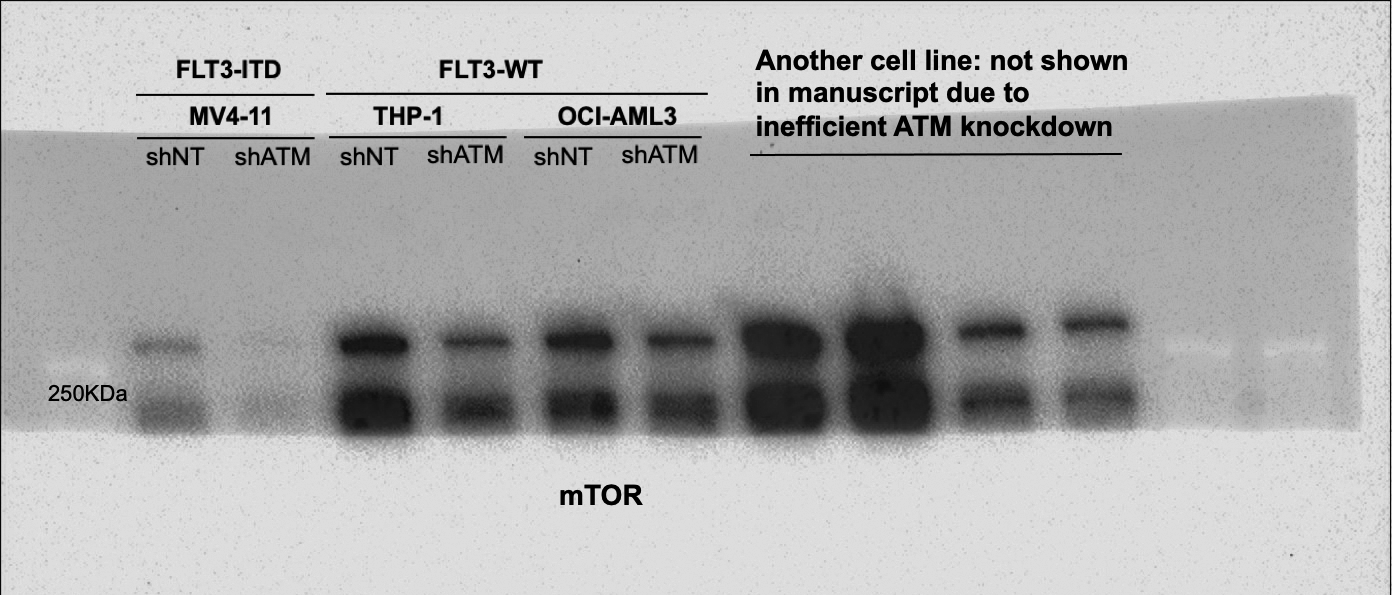

Supplement: Figure 7—figure supplement 6—source data 2. [file elife-79940-fig7-figsupp6-data2.zip › Fig 7- figure supplement 6- mTOR labeled.tiff]

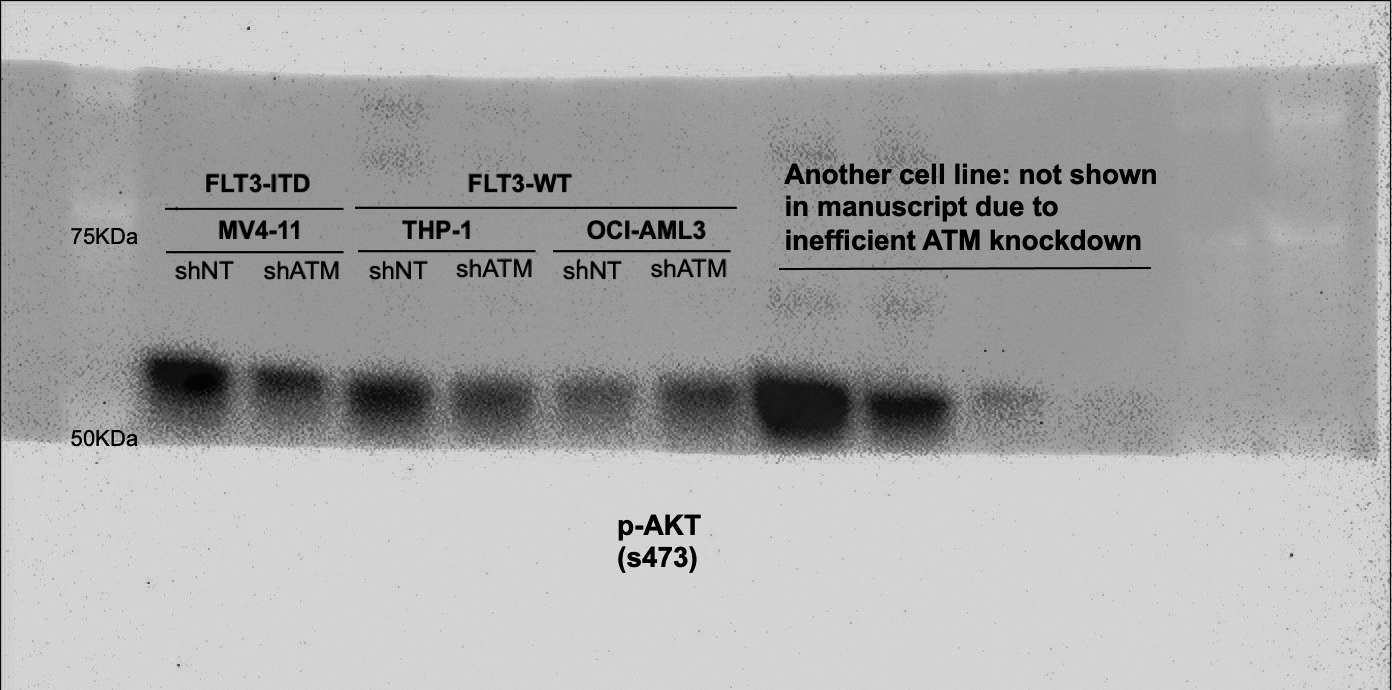

Supplement: Figure 7—figure supplement 6—source data 2. [file elife-79940-fig7-figsupp6-data2.zip › Fig 7- figure supplement 6- pAKT (s473) labeled .tiff]

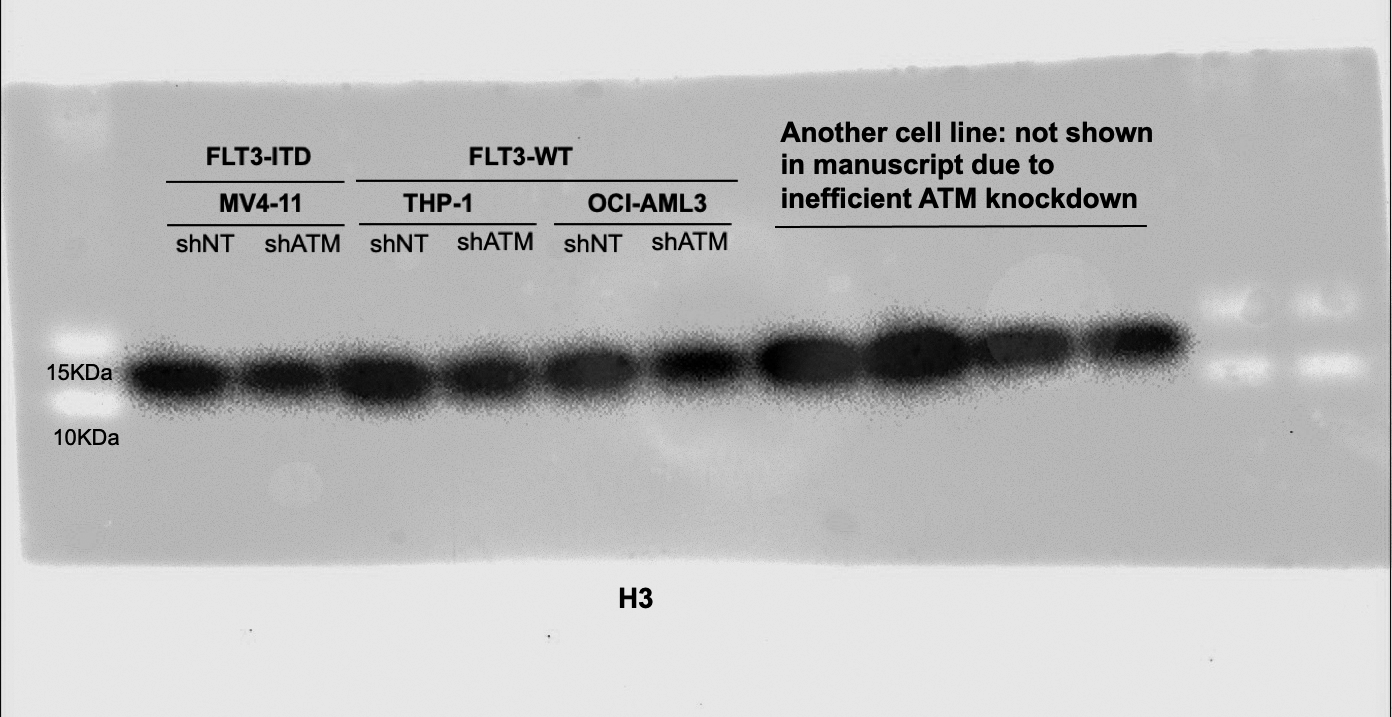

Supplement: Figure 7—figure supplement 6—source data 2. [file elife-79940-fig7-figsupp6-data2.zip › Fig 7- figure supplement 6- H3 labeld.tiff]

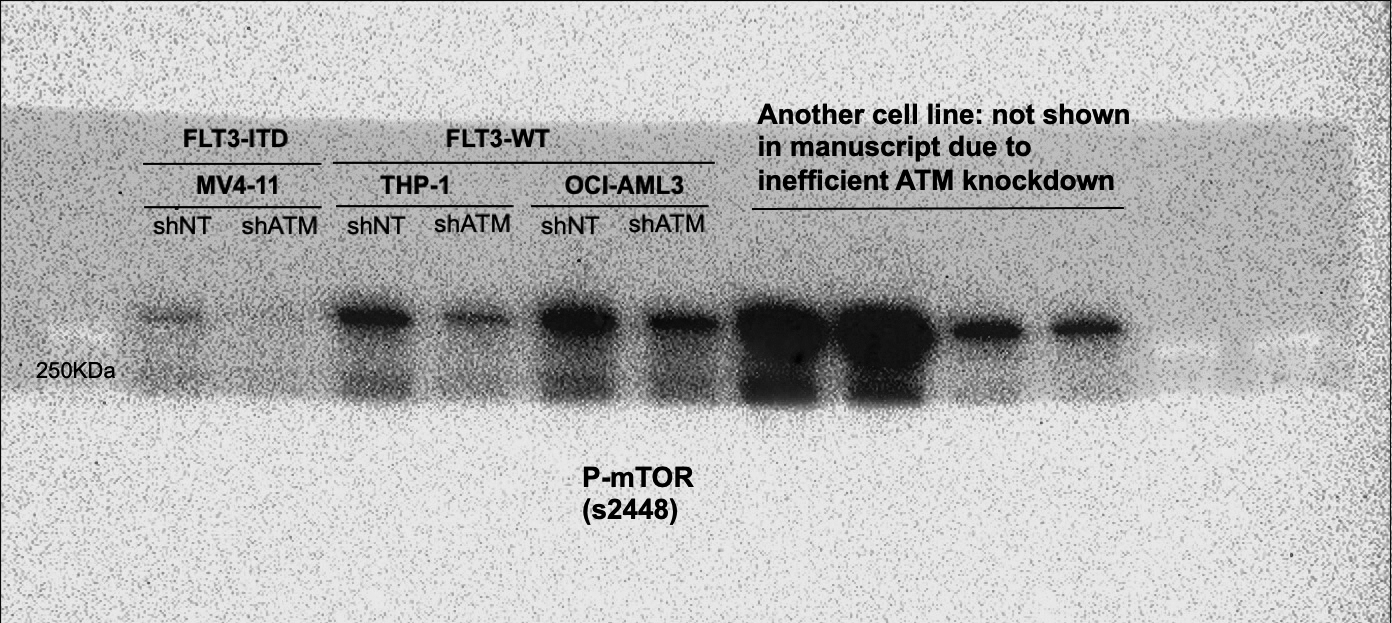

Supplement: Figure 7—figure supplement 6—source data 2. [file elife-79940-fig7-figsupp6-data2.zip › Fig 7- figure supplement 6- pMTOR (s2448) labeled.tiff]

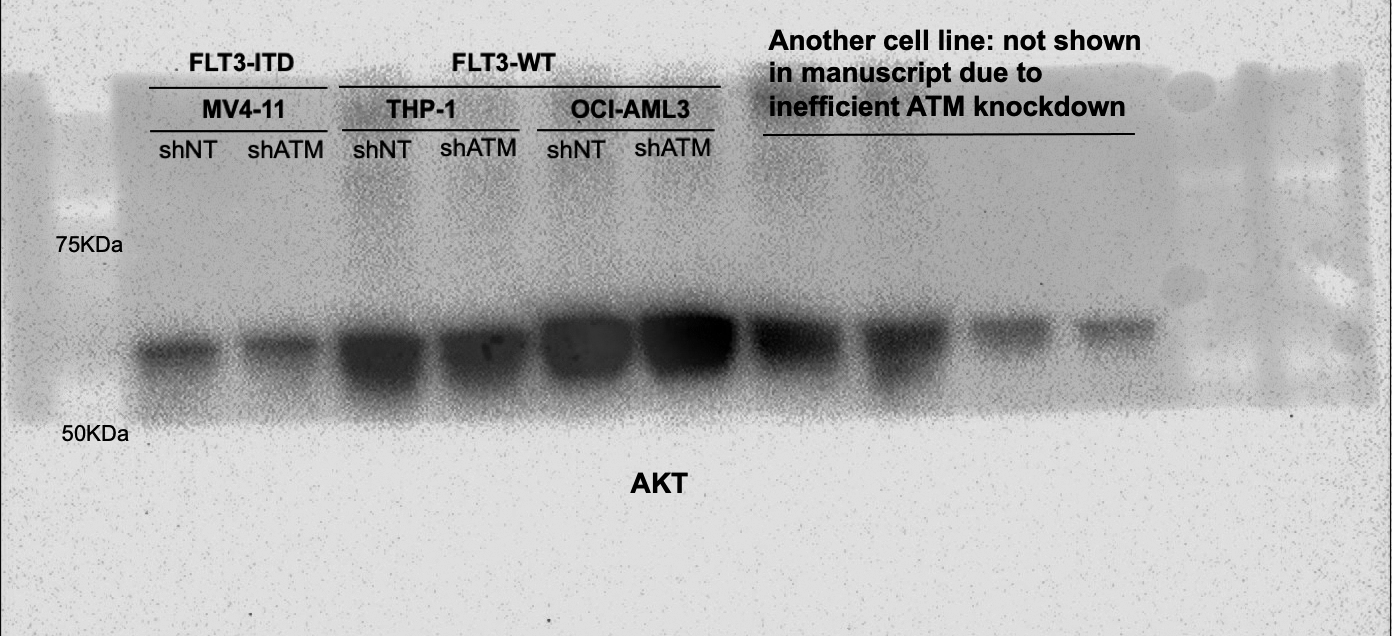

Supplement: Figure 7—figure supplement 6—source data 2. [file elife-79940-fig7-figsupp6-data2.zip › Fig 7- figure supplement 6- AKT labeled.tiff]

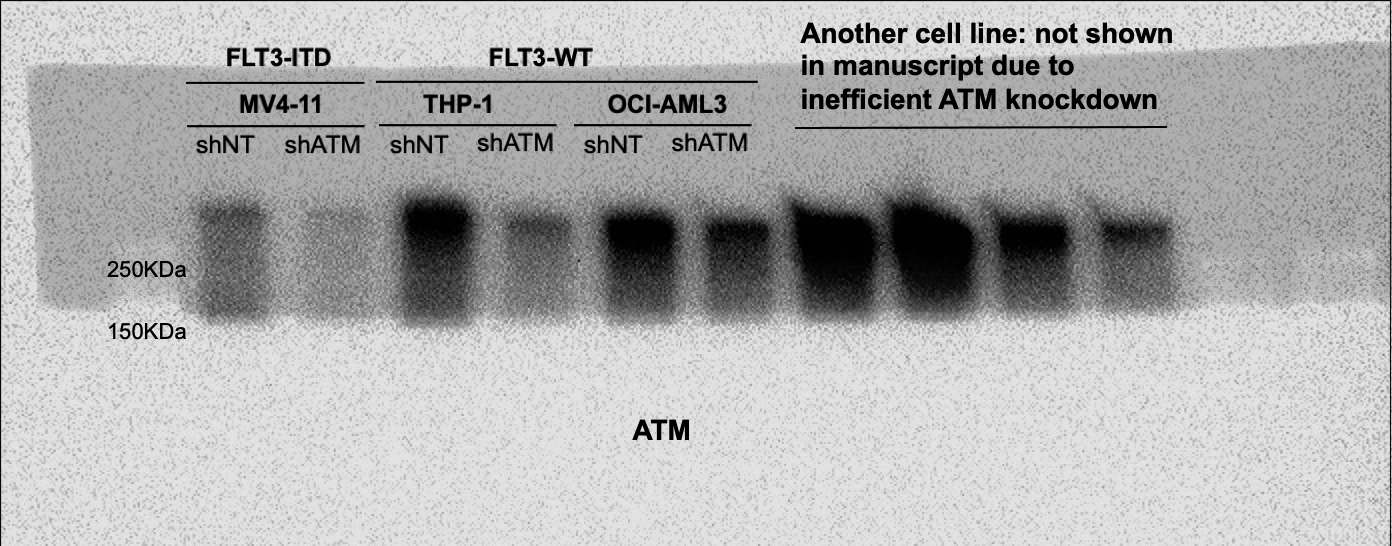

Supplement: Figure 7—figure supplement 6—source data 2. [file elife-79940-fig7-figsupp6-data2.zip › Fig 7- figure supplement 6- ATM labeled.tiff]
